# Development and validation of the predictive aplastic score system (PASS): a simplified tool to diagnose acquired aplastic anemia in adults

**DOI:** 10.1038/s41375-026-02924-3

**Published:** 2026-04-27

**Authors:** Gabriel Aleixo, HeeJin Cheon, Jiayin Zheng, Stephanie Soewito, Jimmy Lee, Eléonore Kaphan, Neha Kalakuntla, Wei-Ying Jen, Sumasri Kotha, Alex Rupsee, Mia Djulbegovic, Jairo A. Matthews, Tapan M. Kadia, Timothy S. Olson, Régis Peffault de Latour, Flore Sicre De Fontbrune, Taha Bat, Courtney D. DiNardo, Daria V. Babushok

**Affiliations:** 1 Division of Hematology-Oncology, Department of Medicine, University of Pennsylvania, Philadelphia, PA USA; 2 Internal Medicine Residency Program, Department of Medicine, University of Pennsylvania, Philadelphia, PA USA; 3 Department of Biostatistics, Epidemiology, and Informatics, Perelman School of Medicine, University of Pennsylvania, Philadelphia, PA USA; 4 Department of Leukemia, Division of Cancer Medicine, The University of Texas MD Anderson Cancer Center, Houston, TX USA; 5 Division of Hematology and Oncology, Department of Internal Medicine, UT Southwestern Medical Center, Dallas, TX USA; 6 APHP, Service D’hématologie Greffe, Hôpital Saint-Louis, Paris, France; 7 School of Medicine, UT Southwestern Medical Center, Dallas, TX USA; 8 Perelman School of Medicine, University of Pennsylvania, Philadelphia, PA USA; 9 Division of Oncology, Department of Pediatrics, Children’s Hospital of Philadelphia, Philadelphia, PA USA; 10 Comprehensive Bone Marrow Failure Center, The Children’s Hospital of Philadelphia, Philadelphia, PA USA; 11 French Reference Center for AA & PNH, Inserm U1342, Saint Louis Research Institute, Team Translational Immunology in Immunotherapy and Hematology, Leukemia Institute Paris Saint Louis, Université Paris Cité, Paris, France

**Keywords:** Anaemia, Translational research, Genetic testing, Immunopathogenesis, Signs and symptoms

## Abstract

Acquired aplastic anemia (AA) can present similarly to inherited bone marrow failure syndromes (IBMFS) but treatment differs. AA diagnosis relies on excluding IBMFS; however, genetic testing is not always available, may delay care or be inconclusive. We developed the Predictive Aplastic Score System (PASS), a clinical tool using readily available data to distinguish AA from IBMFS in adults. The training cohort included 212 adults (162 AA, 50 IBMFS). Compared to IBMFS, AA patients were older and more likely to have acute-onset, severe cytopenias. Using logistic regression with LASSO, we selected seven clinical variables for model inclusion: severity, acuity, age, IBMFS red flags, AA-associated conditions, AA-associated somatic changes, and telomere lengths. The model achieved AUC of 0.990 (95% CI: 0.982–0.999), with 100% positive predictive value (PPV) for AA for scores ≥30. 86.8% of patients with scores <0 had IBMFS. We validated PASS in 716 patients from four external cohorts with AUC of 0.977 (95% CI: 0.968–0.987). Threshold analysis confirmed 100% PPV for scores ≥30, rapidly diagnosing 80% of AA cases. PASS is a practical and accurate clinical tool that can rapidly distinguish AA from IBMFS for most adult patients. To promote clinical adoption, we developed an open-access web calculator (https://pennmedicine.shinyapps.io/passcalc/).

## Introduction

Acquired aplastic anemia (AA) is an immune-mediated bone marrow failure (BMF) disorder caused by an autoreactive T-cell attack on hematopoietic stem and progenitor cells [1]. Most AA patients are previously healthy individuals who present with acute and severe pancytopenia, requiring high-intensity supportive care and urgent bone marrow transplantation (BMT) or immunosuppressive therapy (IST) to restore hematopoiesis [1–4]. Because AA is a diagnosis of exclusion without a definitive test, clinicians must rule out mimicking conditions, including inherited bone marrow failure syndromes (IBMFS), to establish the diagnosis [4–7].

IBMFS are a heterogeneous group of rare genetic diseases that resemble AA but require different treatment. Unlike AA, IBMFS patients do not respond well to standard IST, can have extrahematopoietic manifestations, and can experience severe toxicity with standard BMT conditioning regimens [8, 9]. Due to clinical overlap, limited familiarity, and medicolegal concerns, clinicians increasingly rely on genetic testing “to confirm AA diagnosis” before initiating therapy. However, genetic testing is not always readily available and may be inconclusive [10, 11]. In AA, treatment delays while awaiting testing may worsen cytopenias, increase infection and alloimmunization risk, and reduce IST responsiveness and transplant outcomes [12–17]. Conversely, misdiagnosing IBMFS as AA can lead to inappropriate therapy, increased transplant toxicity, or inadvertent selection of an affected family member as a donor [7, 18–24].

Previous efforts to improve diagnostic precision using machine learning required manual entry of numerous variables, limiting their clinical utility [10]. Universal genetic testing has been proposed [24], and recommendations have been incorporated into guidelines from the NCCN [25], EBMT [26], and the British Society of Hematology [4]. Yet, while increasingly available, genetic testing may be unnecessary for many patients, may leave lingering uncertainties, and may require specialized facilities and long turnaround times for germline tissue analysis. IBMFS genetic testing remains inaccessible in many non-tertiary care and resource-limited settings, contributing to cost and patient anxiety. Extended workups frequently delay definitive therapy while most adult AA patients have no identifiable IBMFS variants. As gene panels expand, rare variants of uncertain significance increase, requiring careful interpretation and counseling [11].

We and others showed that paroxysmal nocturnal hemoglobinuria (PNH) clones and acquired 6p loss of heterozygosity (6pLOH) are strong predictors of AA that effectively discriminate AA from IBMFS [27–31]. However, these are present in only 40–50% of AA patients. Additionally, very small PNH clones can occur in healthy individuals [32–34] and can be difficult to interpret without validated cutoffs [35]. While 6pLOH is highly specific for AA, abnormalities of chromosome arm 6p (including 6pLOH) have been reported in congenital IBMFS, highlighting the limitations of relying solely on these markers [36].

Building on our prior work, we developed the predictive aplastic score system (PASS), a simplified scoring system incorporating readily available clinical and molecular features with high predictive value for distinguishing AA from IBMFS in adults. We validated PASS retrospectively using four independent external datasets and here we report its diagnostic performance in a total of 928 BMF patients (747 AA and 181 IBMFS). PASS offers a practical tool for rapid, accurate diagnosis, minimizing treatment delays and identifying patients most likely to benefit from extended IBMFS testing. Ultimately, PASS aims to reduce diagnostic errors and improve outcomes for patients with AA and IBMFS.

## Materials/Subjects and Methods

See Supplemental Appendix for detailed description of the methods.

### Patients and cohorts

The study was approved by the Institutional Review Boards (IRBs) of the participating institutions. Adults ( ≥ 18 years) evaluated for BMF—defined as cytopenias with a hypocellular marrow after exclusion of secondary causes—were eligible. Only patients diagnosed with AA or IBMFS were included.

The training cohort comprised consecutive adult patients evaluated at the University of Pennsylvania (Penn) between 2010 and 2025, identified through IRB-approved retrospective electronic medical record review and the Penn/CHOP BMF Registry. External validation was performed in four independent adult cohorts: the published NIH/University of São Paulo (NIH/USP) cohort, the French national reference center observatory (RIME), and retrospective cohorts from MD Anderson (MDA) and University of Texas Southwestern (UTSW).

### Diagnostic group assignment

Diagnostic classification followed standardized criteria across cohorts. IBMFS diagnoses were based on genetic testing and syndrome-specific assessments [37, 38], following marrow evaluation and systematic exclusion of alternative etiologies [4, 5, 11, 39]. AA was confirmed by either exclusion of IBMFS on genetic testing and/or sustained response to IST per NIH criteria [40]. Patients lacking confirmatory testing or evaluable IST response were classified as presumed AA in accordance with standard clinical practice [4, 5, 11, 39]. All patients in the NIH/USP cohort had complete IBMFS genetic testing and telomere length (TL) measurements, and all AA patients in the RIME cohort had confirmed AA based on IBMFS genetic testing or evidence of IST.

### Clinical data collection

Clinical, laboratory, and genetic data in the training cohort were obtained by independent manual chart review by two investigators. Collected variables included demographics, cytopenia severity, somatic and cytogenetic findings, presence and size of PNH clones, IBMFS-associated features (IBMFS “red flags”, Supplemental Table S1), AA-associated clinical conditions [41–47] (Supplemental Table S2).Cytopenia severity was classified using the modified Camitta criteria [48, 49].

### PASS score development and validation

PASS was developed in the training cohort using logistic regression with variable selection by least absolute shrinkage and selection operator (LASSO) [50] and 10-fold cross-validation to select the optimal penalization parameter λ that minimized the mean cross-validated binomial deviance (the minimum-deviance criterion). Model coefficients were translated into a simplified integer-based scoring system designed for bedside clinical use. Validation was performed using patient-level data from all cohorts, with missing values scored as absence of abnormal findings.

### Statistical analysis

Discrimination was assessed using ROC AUC, and calibration using calibration plots, Brier scores, and the Hosmer–Lemeshow (HL) test. Predictive values were calculated at prespecified score thresholds. PASS performance was compared with published diagnostic models (the Gutierrez-Rodrigues et al. machine-learning (“NIH-ML”) model [10] and the Kaphan et al. recursive partitioning (RIME) model [51], using ROC AUC, Brier scores, and DeLong’s χ² test for pairwise comparisons. Inter-rater reliability was assessed using Fleiss’ kappa. Analyses were performed using Stata v18.0, and Graphad Prism, with two-sided *p* < 0.05 considered significant.

## Results

### Clinical presentation of AA and IBMFS in adults

The training cohort included 212 patients (median age 51.8 years; range 18.2–86.7). 162 patients (76.4%) had AA, while 50 (23.6%) had IBMFS (Table 1). 51.9% of patients (110 of 212) had no historical normal complete blood count documented prior to presentation. TL testing by flow FISH was available for 116 patients (54.7%), chromosome breakage testing for 90 (42.5%) patients, and genetic testing for IBMFS-associated genes for 65 (30.7%) patients (Supplemental Table S3). 151 AA patients (93.2%) were treated with IST, and 85.7% (120 of 140 evaluable patients) achieved a partial or complete hematologic response at 6 months. Based on our diagnostic adjudication criteria for this study, 80.2% of AA patients (130 of 162) were further classified as having confirmed diagnosis of AA by virtue of hematologic response to IST or negative IBMFS genetic testing, while 32 were categorized as having a presumed diagnosis of AA based on expert evaluation and exclusion of other conditions (Supplemental Table S4).

**Table 1:** Clinical Characteristics of the Training Cohort.

Patient Characteristic		Total Patients (*n* = 212)	AA (*n* = 162)	IBMFS (*n* = 50)
Male, n (%)		114 (53.8%)	80 (49.4%)	34 (68.0%)
Female, n (%)		98 (46.2%)	82 (50.6%)	16 (32.0%)
Age at diagnosis, years, median (range)		51.8 (18.2-86.7)	54.7 (19.3–86.7)	37.4 (18.8–72.4)
	Patients age ≥ 60 years, n (%)	74 (34.9%)	63 (38.9%)	11 (22.0%)
	Patients age < 60 years, n (%)	138 (65.1%)	99 (61.1%)	39 (78.0%)
Race, n (%)	White	161 (75.9%)	123 (75.9%)	38 (76.0%)
	Asian	24 (11.3%)	17 (10.5%)	7 (14.0%)
	Black or African American	22 (10.3%)	19 (11.7%)	3 (6.0%)
	Interracial	2 (0.9%)	2 (1.2%)	0 (0.0%)
	NA	3 (1.4%)	1 (0.6%)	2 (4.0%)
Ethnicity, n (%)	Hispanic or Latino	6 (2.8%)	6 (3.7%)	0 (0.0%)
	Non-Hispanic or Latino	206 (97.2%)	156 (96.3%)	50 (100.0%)
Chronicity of cytopenias	Cytopenia duration, years, median (range)	0.2 (0.0–lifelong)	0.1 (0.0–11.6)	4.0 (0.0–lifelong)
	Patients with chronic cytopenias ( > 1 year), n (%)	61 (28.8%)	19 (11.7%)	42 (84.0%)
	Patients with acute cytopenias ( ≤ 1 year), n (%)	151 (71.2%)	143 (88.3%)	8 (16.0%)
Previously normal CBC	Patients with documented prior normal CBC, n (%)	102 (48.1%)	89 (54.9%)	13 (26.0%)
	No documentation of prior normal CBC, n (%)	110 (51.9%)	73 (45.1%)	37 (74.0%)
Cytopenia severity, n (%)	NSAA	79 (37.3%)	33 (20.4%)	46 (92.0%)
	SAA	113 (53.3%)	109 (67.3%)	4 (8.0%)
	VSAA	20 (9.4%)	20 (12.3%)	0 (0.0%)
Median lymphocyte telomere lengths	Normal, n (% evaluable)	62 (53.4%)	55 (71.4%)	7 (17.9%)
	1-10th, n (% evaluable)	30 (25.9%)	20 (26.0%)	10 (25.6%)
	<1st, n (% evaluable)	24 (20.7%)	2 (2.6%)	22 (56.4%)
	NA, n	96	85	11
Median granulocyte telomere lengths	Normal, n (% evaluable)	22 (23.9%)	18 (32.1%)	4 (11.1%)
	1-10th, n (% evaluable)	20 (21.7%)	15 (26.8%)	5 (13.9%)
	<1st, n (% evaluable)	50 (54.3%)	23 (41.1%)	27 (75.0%)
	NA	120	106	14
PNH clone in granulocytes, patients, n (%)	None (0)	83 (49.7%)	67 (44.4%)	16 (100.0%)
	small <0.5%	19 (11.4%)	19 (12.6%)	0 (0.0%)
	0.5 to <1%	6 (3.6%)	6 (4.0%)	0 (0.0%)
	≥1%	31 (18.6%)	31 (20.5%)	0 (0.0%)
	≥10%	28 (16.8%)	28 (18.5%)	0 (0.0%)
	NA	45	11	34
6pLOH, patients, n (%)	Present	11 (11.3%)	10 (13.7%)	1 (4.2%)
	Absent	86 (88.7%)	63 (86.3%)	23 (95.8%)
	NA	115	89	26
Patients with somatic mutations, n (%)	*BCOR/BCORL1*	18 (16.5%)	18 (17.1%)	0 (0.0%)
	*ASXL1*	14 (12.8%)	14 (13.3%)	0 (0.0%)
	*DNMT3A*	21 (19.3%)	20 (19.0%)	1 (4.2%)
	*TET2*	4 (3.7%)	3 (2.9%)	1 (4.2%)
	*RUNX1*	8 (7.3%)	6 (5.7%)	2 (8.3%)
	*PHF6*	3 (2.8%)	3 (2.9%)	0 (0.0%)
	*SETBP1*	4 (3.7%)	3 (2.9%)	1 (4.2%)
	Other	23 (21.1%)	13 (12.4%)	10 (41.7%)
	None	59 (54.1%)	47 (44.8%)	12 (50.0%)
	NA	83	57	26
Patients with cytogenetic abnormalities^a^, n (%)	Normal	144 (74.6%)	122 (79.2%)	22 (56.4%)
	del(13)(q)	9 (4.7%)	9 (5.8%)	0 (0.0%)
	-Y	6 (3.1%)	6 (3.9%)	0 (0.0%)
	+8	4 (2.1%)	3 (1.9%)	1 (2.6%)
	-7	8 (4.1%)	7 (4.5%)	1 (2.6%)
	del(20)(q)	4 (2.1%)	2 (1.3%)	2 (5.1%)
	Other	14 (7.3%)	8 (5.2%)	6 (15.4%)
	NA	19	8	11
Treated with IST, n (%)		154 (72.6%)	151 (93.2%)	3 (6.0%)
Response to IST at 6 months, n (%)	CR	41 (28%)	41 (29.3%)	0 (0.0%)
	PR	79 (37%)	79 (56.4%)	0 (0.0%)
	No response	23 (11%)	20 (14.3%)	3 (100.0%)
	No IST or not evaluable	66	22	47
Received BMT, n (%)		48 (22.6%)	42 (25.9%)	6 (12.0%)
Progression to myeloid malignancies, n (%)		22 (10.3%)	15 (9.3%)	6 (12.0%)
Conditions historically associated with AA	Eosinophilic fasciitis	1 (0.5%)	1 (0.6%)	0
	Seronegative hepatitis	2 (0.9%)	2 (1.2%)	0
	Hodgkins’s lymphoma	2 (0.9%)	2 (1.2%)	0
	Previously tolerated chemotherapy or radiation	3 (1.4%)	3 (1.9%)	0
	Thymoma	1 (0.5%)	1 (0.6%)	0
	Use of immune checkpoint inhibitor	1 (0.5%)	1 (0.6%)	0
	None	202 (95.3%)	152 (93.8%)	50 (100.0%)
IBMFS red flag conditions	Patients with any red flags, n (%)	51 (24.1%)	6 (3.7%)	45 (90.0%)
	Congenital abnormality, or dysmorphic features	22	4	18
	Interstitial lung disease, avascular necrosis, or unexplained liver cirrhosis	18	0	18
	Mucocutaneous triad, of nail dystrophy, skin hyperpigmentation, oral leukoplakia	3	0	3
	Unexpected hematologic toxicity with failure to recover blood counts after chemotherapy or radiation	2	1	1
	Refractory warts or a history of non-tuberculous mycobacterial infection	3	0	3
	Squamous cell cancer of the head and neck or anogenital region	6	2	4
	First-degree relative with a diagnosis of bone marrow failure or thrombocytopenia, MDS, AML or one of the conditions described above	19	3	16
Duration of follow-up, years, median (range)		3.5 (0.0–20.7)	3.7 (0.1–20.7)	2.4 (0.0–13.5)

^a^Isolated cytogenetic abnormalities. *AA* acquired aplastic anemia. *IBMFS* inherited bone marrow failure syndromes. *CBC* complete blood count, *IST* immunosuppression therapy, *CR* complete response, *PR* partial response, *BMT* bone marrow transplant, *PNH* paroxysmal nocturnal hemoglobinuria, *6pLOH* acquired chromosome arm 6p loss of heterozygosity, *MDS* myelodysplastic neoplasm, *AML* acute myeloid leukemia.

Of the IBMFS cases, 48 of 50 (96.0%) had a confirmed diagnosis (25 (50.0%) had telomere biology disorders (TBD), 8 (16.0%) had Fanconi anemia (FA), 5 (10.0%) had Diamond-Blackfan anemia (DBA), 3 (6.0%) had GATA2 deficiency, and 7 (14%) had other defined congenital syndromes), while 2 (4.0%) carried a clinical diagnosis of IBMFS without a genetic cause identified (Supplemental Tables S5). Of the IBMFS patients, 39 (78.0%) presented with hematologic manifestations, while 11 (22.0%) were diagnosed during evaluation or treatment of extra-hematopoietic features (e.g., pulmonary fibrosis in TBD, failure to recover blood counts after chemotherapy for solid tumor in FA). Three patients with IBMFS initially received IST for treatment of presumed AA without hematologic response before an eventual diagnosis of IBMFS—one based on a newly reported *TUBB* variant identified on re-analysis of prior whole-exome sequencing [36], one due to new clinical manifestations of TBD in the absence of an identified germline mutation, and the third was found to have genetically-confirmed GATA2 deficiency after GATA2 deficiency syndrome was described in the literature.

**Fig. 1:**
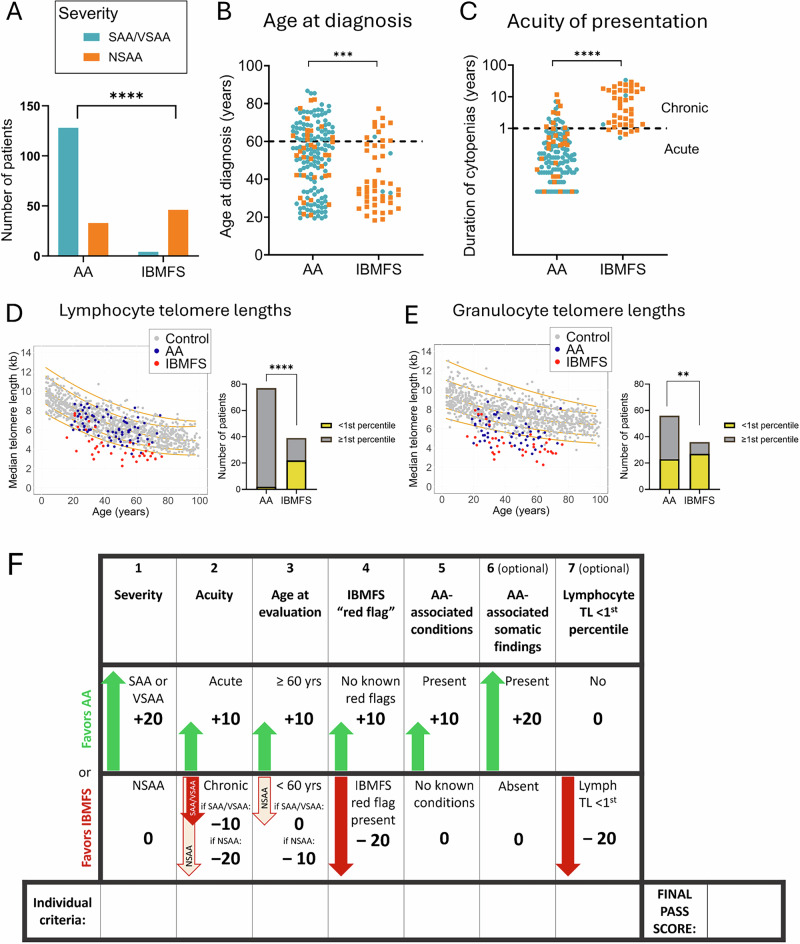
Defining clinical characteristics of AA and IBMFS. **A** Disease severity at presentation. Bar plot showing numbers of patients with severe or very severe aplastic anemia (SAA/VSAA, teal) and non-severe aplastic anemia (NSAA, orange) among those with AA and IBMFS. **B** Shown is the distribution of ages (in years) at the time of bone marrow failure diagnosis, with orange dots showing patients presenting with NSAA and teal for those presenting with SAA/VSAA. The dashed line marks the 60-year age, which was used for subsequent regression analyses. **C** Acuity of presentation, defined by the duration of cytopenias from the first abnormal CBC to diagnosis. Patients with AA typically presented acutely, defined as within 1 year of cytopenia onset, whereas those with IBMFS more often had a chronic course extending beyond 1 year. Disease severity is also indicated by orange (NSAA) and teal (SAA/VSAA) dots. The dashed line marks the 1-year threshold used to define acute versus chronic presentation. **D**,**E** Median telomere lengths in lymphocytes (**D**) and granulocytes (**E**) measured by flow-FISH analysis for 77 AA (blue dots) and 39 IMBFS patients (red dots) with available raw telomere lengths data. Telomere length distributions from healthy controls are shown for reference (gray dots), based on published data from Johns Hopkins (*n* = 192) and Vancouver (*n* = 444), with percentile curves in orange (1st, 10th, 50th, 90th, and 99th percentiles, from bottom to top). To the right of each telomere length scatter plot are the corresponding summary analysis depicted in stacked bar plots showing the number of AA and IBMFS patients with telomeres <1st percentile in lymphocytes (**D**), and granulocytes (**E**). Statistical significance was determined using Fisher’s exact test for categorical variables (**A**,**D**,**E**) and two-sided Student’s t tests for continuous variables (**B**,**C**). p values are indicated as follows: **, *p* < 0.01; ***, *p* < 0.001; **** *p* < 0.0001 (****). **F** Schematic demonstrating the PASS score calculation. The PASS score is calculated by adding individual criteria contributions from seven clinical factors: **(1)** Cytopenia severity, with +20 points assigned for SAA/VSAA (defined as two or more of the following: absolute neutrophil count (ANC) < 0.5·10^3^ cells/μL, anemia with an absolute reticulocyte count <60·10^3^ cells/μL, platelets < 20·10^3^ cells/μL), and 0 points assigned for NSAA (defined as having one or fewer severe cytopenias defined above). (2) Acute presentation, defined as cytopenias of 1 year or less in duration (or having no documented previous abnormal blood count with cytopenias presumed to be acute) are assigned +10 points. Chronic presentation, defined as cytopenias >1 year duration, are assigned either −10 points in patients with SAA/VSAA cytopenias, or -20 points in patients with NSAA cytopenias. (3) Patients being evaluated at 60 years and older are given +10 points, while those under 60 years at the time of evaluation are assigned 0 points in patients with SAA/VSAA cytopenias or −10 points in patients with NSAA cytopenias. (4) IBMFS red flag is assigned –20 points if any of the red flag conditions are present; patients are assigned +10 points if there are no red flags. IBMFS red flag is defined as having any of the following i) congenital abnormality, including abnormal thumb, or dysmorphic features, ii) Interstitial lung disease, avascular necrosis, or unexplained liver cirrhosis, iii) Mucocutaneous triad of nail dystrophy, skin hyperpigmentation, oral leukoplakia, iv) Unexpected hematologic toxicity with failure to recover blood counts after chemotherapy or radiation, v) Refractory warts or a history of non-TB mycobacterial infection, vi) Squamous cell cancer of the head and neck or anogenital region, vii) First-degree relative with a diagnosis of bone marrow failure or thrombocytopenia, MDS, AML, or one of the red flag conditions. (5) Presence of rare conditions etiologically related to AA or incompatible with IBMFS (“AA-associated conditions”) is assigned +10 points. Absence of these conditions is given 0 points. AA-associated conditions are defined as: i) Seronegative autoimmune hepatitis, ii) Treatment with immune checkpoint inhibitors, iii) Known diagnosis of immune dysregulation syndrome (e.g., CTLA4 haploinsufficiency), iv) History of eosinophilic fasciitis, v) History of thymoma, vi) History of Hodgkin’s lymphoma, vii) Patient previously tolerated cytotoxic chemotherapy without unexpected hematologic toxicity (e.g., historical treatment for breast cancer or lymphoma) non-contiguous to current episode of BMF. (6) Presence of clonal hematopoiesis with any one of the following AA-associated acquired somatic changes is assigned +20 points: PNH granulocyte clone ≥0.5%, acquired chromosome arm 6p loss of heterozygosity (6pLOH), acquired del(13)(q) as an isolated abnormality, and somatic mutation in *BCOR* or *BCORL1* genes. Absence of these is given 0 points. (7) Median lymphocyte telomere lengths <1st percentile are given −20 points, while longer telomere lengths of 1st percentile and higher are given 0 points. Note: Of the seven individual score criteria, factors 1–5 are required to calculate the PASS score. Factors 6 and 7 are not required, but recommended when available and can be added later as they become available for greater diagnostic accuracy. Missing values are assigned 0.

Clinical and laboratory features diverged sharply between AA and IBMFS groups (Tables 1–2, Fig. 1). The most striking differences between groups were in the acuity and severity of cytopenias (Fig. 1A–C): 118/162 (72.8%) of AA patients had acute-onset ( < 1 year) severe or very severe AA (SAA/VSAA), compared to only 1/50 (2.0%) IBMFS cases (*p* < 0.001). Conversely, chronic ( > 1 year) non-severe cytopenias (NSAA) predominated in IBMFS (39/50 [78.0%]) compared to AA (8/162 [4.9%]; *p* < 0.001).

**Table 2:** Logistic Regression and LASSO Analysis Identifying Seven Clinical Variables for Inclusion in the PASS Model.

				Univariate logistic regression			Multivariate logistic regression				LASSO Logistic Regression
Clinical Variable		AA n (%)	IBMFS n (%)	OR	95% CI	*p*-value	OR	95% CI		*p*-value	Coefficient (β)
**Age** ≥ **60 years**	≥ 60 years	63 (38.9%)	11 (22.0%)	2.256	1.077–4.729	**0.031**	21.082	0.387-1147.5		0.135	0.537
	< 60 years	99 (61.1%)	39 (78.0%)								
**Cytopenia severity**	SAA/VSAA	129 (79.6%)	4 (8.0%)	44.955	15.101 -133.830	**< 0.001**	94.040	2.573-3436.7		**0.013**	2.475
	NSAA	33 (20.4%)	46 (92.0%)								
**Acuity of presentation**	Acute ( ≤ 1 yr)	143 (88.3%)	8 (16.0%)	39.513	16.148 -96.686	**< 0.001**	7.175	1.209-42.559		**0.030**	1.515
	Chronic ( > 1 yr)	19 (11.7%)	42 (84.0%)								
**AA-associated somatic changes**^a^	Present	85 (52.5%)	1 (2.0%)	54.091	7.293–401.190	**< 0.001**	4.999	0.359-69.652		0.231	1.217
	Absent	77 (47.5%)	49 (98.0%)								
**AA-associated conditions**^**b**^	Present	10 (6.2%)	0 (0.0%)	6.954	0.400–120.809	0.183	n/a				0.656
	Absent	152 (93.8%)	50 (100%)								
**IBMFS red flags**^**c**^	Present	6 (3.7%)	45 (90%)	0.004	0.001–0.014	**< 0.001**	0.002	0.000–0.109		**0.002**	–3.933
	Absent	156 (96.3%)	5 (10.0%)								
**Lymphocyte telomere lengths <1st** **percentile**	<1st percentile	2 (2.6%)	22 (56.4%)	0.021	0.004–0.096	**< 0.001**	0.042	0.001–1.284		0.069	–1.575
	≥1st percentile	75 (97.4%)	17 (43.6%)								
**Diagnostic Performance of the PASS Model in Training and Validation Cohorts**											
**Cohort**	**Patients, n**	**AA, n**	**IBMFS, n**	**ROC AUC (95% CI)**		**Brier Score**	**PPV for AAPASS** ≥ **30**		**PPV for IBMFS PASS** < **0**		**PPV for AA PASS 0 ‒ 20**
**Training (Penn)**	212	162	50	0.990 (0.982– 0.999)		0.035	100% (136/136)		86.8% (46/53)		82.6% (19/23)
**Combined Validation**	716	585	131	0.977 (0.968–0.987)		0.044	100% (463/463)		81.6% (115/141)		85.7% (96/112)
**RIME**	270	241	29	0.985 (0.974– 0.997)		0.031	100% (206/206)		77.4% (24/31)		84.8% (28/33)
**NIH/USP**	247	203	44	0.969 (0.948– 0.990)		0.048	100% (147/147)		75.5% (40/53)		91.5% (43/47)
**MDA**	121	69	52	0.979 (0.957– 1.000)		0.048	100% (56/56)		94.0% (47/50)		66.7% (10/15)
**UTSW**	78	72	6	0.955 (0.898– 1.000)		0.037	100% (54/54)		57.1% (4/7)		88.2% (15/17)
**Training and Validation Combined**	928	747	181	0.981 (0.974– 0.988)		0.042	100% (599/599)		83.0% (161/194)		85.2% (115/135)

^a^Presence of one or more PNH granulocyte clone ≥0.5%, somatic *BCOR* or *BCORL1* mutations, isolated del(13)(q) cytogenetic abnormality, acquired 6pLOH involving the HLA gene region on cytogenomic array.

^b^see Supplemental Table S2.

^c^see Supplemental Table S1. P-value in bold indicates statistical significance. *AA* aplastic anemia, *IBMFS* inherited bone marrow failure syndromes, *OR* odds ratio, *CI* confidence interval.

AA patients were older at presentation (median 54.7 years; range 19.3–86.7) compared to IBMFS patients (median 37.4 years; range 18.8–72.4; *p* = 0.001) (Fig. 1B). Both groups had patients presenting at age ≥60 years, but presentation in the older age range was more common for AA (AA: 63/162 [38.9%] vs. IBMFS: 11/50 [22.0%]; *p* = 0.031).

Clinical stigmata or family history suggestive of IBMFS were present in 45 of 50 IBMFS patients (90.0%) versus 6 of 162 (3.7%) AA patients (*p* < 0.001, Table 2). A subset of AA patients (10 of 162, 6.2%) had conditions associated with AA (e.g., checkpoint inhibitor use, seronegative hepatitis) [41–44, 47]; these were absent in IBMFS patients.

Clonal hematopoiesis was identified by clinical testing in 142 patients (67.0% of the cohort). Somatic changes characteristic of AA were present in 52.5% of AA patients (85/162) (Tables 1–2) and included granulocyte PNH clones ≥0.5% in 43.0% (65/151) of evaluable patients, acquired 6pLOH in 13.7% (10/73) of evaluable patients, somatic mutations in *BCOR* or *BCORL1* in 17.1% (18/105), and del(13)(q) as an isolated abnormality in 5.8% (9/154). These AA-associated somatic findings were rare in IBMFS (1 of 50 [2.0%]; *p* < 0.001).

Among patients with TL measurements, fewer AA patients had lymphocyte TLs <1st percentile for age (2/77 [2.6%]) compared to IBMFS (22/39 [56.4%], *p* < 0.001), of whom 17 had TBD and five had other IBMFS (Fig. 1D, Table 2). The two AA patients with TLs <1st percentile lacked TBD-associated germline variants or clinical features beyond BMF; one responded to IST, while the other had refractory AA and underwent BMT. Lymphocyte TLs above 10th percentile were found in 71.4% (55/77) of AA vs 17.9% of IBMFS patients (*p* < 0.001); of the seven IBMFS patients with lymphocyte TLs>10th percentile, two had FA, 1—DBA, 1—GATA2 deficiency and three had various other IBMFS. Granulocyte TLs were less discriminatory between AA and IBMFS, with 41.1% of evaluable AA patients (23 of 56) having granulocyte TLs <1st percentile compared to 75.0% (27 of 36) for IBMFS (*p* = 0.002) (Fig. 1E). Only 32.1% (18 of 56) AA patients had granulocyte TLs over 10th percentile.

### Development and performance of the PASS model in the training cohort

We next performed the LASSO logistic regression on the seven candidate variables identified in our univariate analysis (Table 2). LASSO is a logistic regression technique that optimizes variable selection by adding a penalty for inclusion of too many variables that could lead to overfitting. LASSO retained all seven variables with the optimal penalty (λ 0.007). We assigned points ( ± 10 or ±20) based on LASSO coefficient direction, with positive values for factors associated with AA and negative values for those linked to IBMFS (Fig. 1F).

**Fig. 2:**
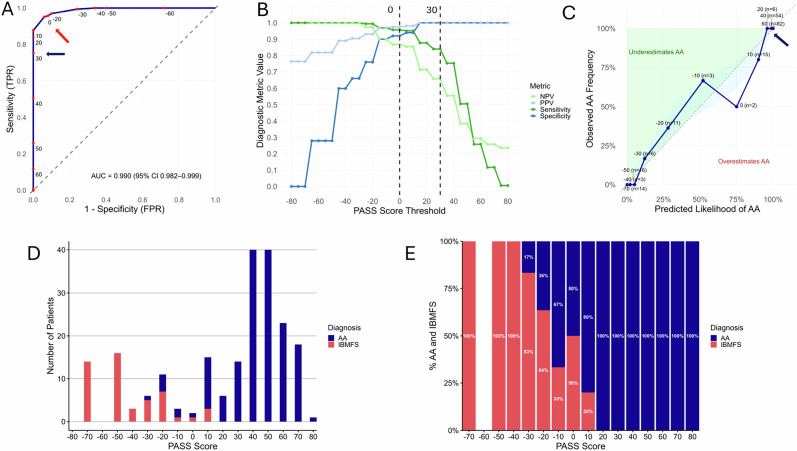
The PASS model performance in the training cohort. **A** The ROC curve, showing the sensitivity (true positive rate) on the Y-axis against the false positive rate (1 − specificity) on the X-axis for scores generated with the PASS model in the training cohort. The red dots correspond to labeled scores in the training cohort. Ideal discrimination allows perfect sensitivity without false positives (upper left quadrant). The dashed diagonal line indicates no discrimination between true and false positives (random). The PASS score shows an excellent area under the curve (AUC), indicating near-perfect diagnostic performance. Blue arrow points to the score of 30, and red arrow to the score of 0, which were chosen as score thresholds for PASS. **B** A plot demonstrating the PASS score performance characteristics (plotted on the Y-axis) across a range of score thresholds (on the X-axis). Plotted are the PPV (light blue), specificity (dark blue), NPV (light green), and sensitivity (dark green). The dashed vertical lines are shown at a score of 30 (demarcating scores with 100% PPV and specificity for AA), and at a score of 0 (demarcating a threshold below which the probability of an AA diagnosis starts to sharply fall and IBMFS diagnosis is more likely). **C** This panel shows a calibration plot comparing predicted probability of AA derived from a logistic regression model (PASS score as predictor) on x-axis with observed frequency of AA on the y-axis. Each point represents a bin of rounded predicted probabilities, annotated with average PASS score and sample size. The dashed diagonal line indicates perfect calibration. The shaded blue region reflects a LOESS-smoothed fit with 95% confidence interval. Green and red annotations highlight regions of systematic underestimation and overestimation, respectively. The model performs well at the extremes, where very low and very high predicted probabilities align with observed outcomes. Arrow points to the region of the plot corresponding to the threshold of 30. **D** Distribution of AA and IBMFS diagnoses across the range of PASS scores (on X-axis), demonstrating excellent separation between the two diagnostic categories, with high specificity for AA for positive PASS scores of 30 and higher. **E** Proportion of AA and IBMFS diagnoses across the range of PASS scores, showing that lower scores are strongly enriched for IBMFS while higher scores are enriched for AA, with near-complete separation at thresholds above 20.

To evaluate model performance, we next applied the PASS score to our training cohort of 212 patients (Fig. 2A-E, Table 2). The score demonstrated excellent diagnostic discrimination, with an area under the ROC curve AUC of 0.990 (95% confidence interval [CI]: 0.982–0.999) (Fig. 2A). Calibration analysis showed strong agreement between predicted and observed probabilities (Fig. 2C) and the Brier score showed good calibration and high predictive performance at 0.035.

The positive predictive value (PPV) and specificity for AA increased with higher PASS scores (Fig. 2B). In the training cohort, scores ≥30 were associated with a 100% PPV for AA and predicted response to IST, with 88.0% (103 of 117) of evaluable patients responding at 6 months, compared to 65.3% (17 of 26) for scores <30 (*p* = 0.004). PPV for IBMFS increased with lower scores (Fig. 2B, D, E), with 86.8% of patients (46 of 53) with scores <0 having IBMFS. Among patients with intermediate scores (0 to 20), the majority (82.6%, 19 of 23) had AA; of those treated with IST, 72.2% (13 of 18) responded at 6 months.

Incorporating the most sensitive threshold for PNH clone detection including clones <0.5% and recalculating the score as new laboratory findings (e.g., AA-associated somatic changes) emerged later in the disease course resulted in an AUC 0.992 (95% CI 0.985-0.999), slightly improving sensitivity from 0.840 to 0.877 while retaining 100% PPV for AA for scores ≥ 30 (Supplemental Fig. S2). Because this improvement was small, we retained the more conservative PNH clone threshold of ≥0.5% to maximize diagnostic specificity, given prior reports of very small PNH clones in rare IBMFS patients [35].

### PASS Score validation in external BMF cohorts

We next assessed the performance of the PASS score in four independent external validation cohorts of adult BMF patients: (1) 270 patients from the French national reference database (RIME) [51], (2) 247 patients from a published dataset from the NIH/USP [10], (3) 121 patients from MDA, and (4) 78 patients from UTSW. The four cohorts had different prevalences of IBMFS patients, reflecting variations in referral practices and patient recruitment: IBMFS comprised 10.7% (29 of 270) in the RIME cohort, 17.8% (44 of 247) in the NIH/USP cohort, 7.7% (six of 78) in the UTSW cohort, and 42.9% (52 of 121) in the MDA cohort. Similar to the training dataset, the most common IBMFS across all validation cohorts were TBDs, comprising a median of 65.5% (range 50–100%) of IBMFS diagnoses (Supplemental Table S6A-D), reflecting TBDs being the most common IBMFS in adults.

Across these cohorts, clinical presentations of AA and IBMFS were consistent with the training dataset (Supplemental Tables S7–S10). Most IBMFS patients presented with NSAA: 69.0% in RIME, 90.9% in NIH/USP, 83.3% in UTSW, and 94.2% in MDA. The majority of AA patients presented with acute-onset cytopenias—97.9% in RIME, 98.0% in NIH/USP, 88.9% in UTSW, and 91.3% in MDA. Most AA patients had severe or very severe cytopenias (SAA/VSAA), observed in 84.2% in RIME, 69.5% of NIH/USP, 61.1% of UTSW, and 84.1% of MDA patients. In contrast, IBMFS patients were more likely to have lymphocyte TL <1st percentile, and few or no IBMFS patients exhibited AA-associated somatic alterations. IBMFS “red flags” were present in 82.8% in RIME, 52.3% of NIH/USP, 83.3% of UTSW, and 80.8% of MDA patients with IBMFS.

**Fig. 3:**
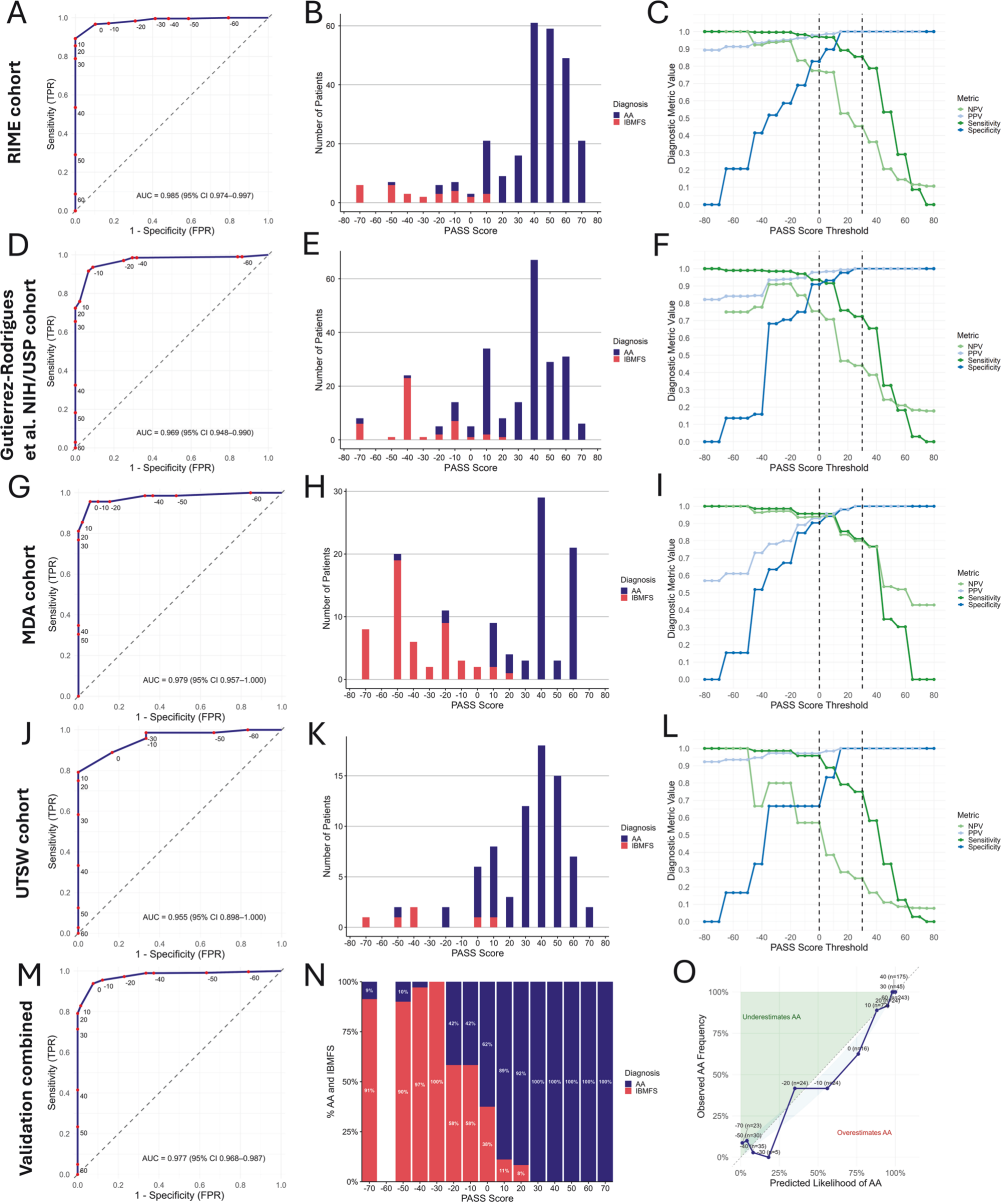
The PASS model performance in four external validation cohorts. Panels are grouped by cohort: **A–C:** RIME, **D–F:** NIH/USP**, G–I:** MDA, **J–L:** UTSW, and **K-O:** combined cohort. **A**,**D**,**G**,**J**,**K** ROC curves showing sensitivity (Y-axis) versus false positive rate (X-axis) for PASS scores in each validation cohort. Red dots indicate labeled scores from the training cohort. The dashed diagonal line represents random classification; ideal discrimination lies in the upper left quadrant. Across all cohorts, the PASS model demonstrates excellent diagnostic performance, with high area under the curve (AUC), reflecting strong separation between AA and IBMFS**. B**,**E**,**H**,**K** Distribution of AA and IBMFS diagnoses across PASS scores. AA diagnoses cluster at higher scores, with only AA diagnoses scoring at 30 and higher, and IBMFS clustering at lower scores. **C**,**F**,**I**,**L** Diagnostic performance metrics across score thresholds, including PPV (light blue), specificity (dark blue), NPV (light green), and sensitivity (dark green). Vertical dashed lines mark thresholds of 0 and 30, which delineate low and high likelihood of AA, respectively. Scores ≥30 yield 100% PPV and specificity for AA, while scores <0 are associated with a sharp decline in AA probability and increased likelihood of IBMFS. These patterns were consistent across all three validation cohorts. **N** Proportion of AA and IBMFS diagnoses across the range of PASS in the combined validation cohort. Lower PASS scores were predominantly associated with IBMFS, while higher scores were enriched for AA, with near-complete separation achieved at thresholds above 20. **O** Calibration plot for the combined validation cohorts, comparing predicted probability of AA (X-axis) with observed frequency (Y-axis). The dashed diagonal line indicates perfect calibration. A LOESS-smoothed fit with 95% confidence interval is shown within the blue shaded region. The model demonstrates good calibration across the full range of predicted probabilities, with close alignment between predicted and observed frequencies, particularly at the high scores where discrimination is strongest.

Application of the PASS score to the four datasets demonstrated excellent discriminatory performance (Fig. 3 A-L, Table 2), with ROC AUCs of 0.977 (95% CI: 0.968–0.987) in all combined cohorts (Fig. 3M), and ROC AUC of 0.985 (95% CI: 0.974-0.997 RIME), ROC AUC of 0.969 (95% CI: 0.948–0.990 NIH/USP), ROC AUC of 0.979 (95% CI: 0.957–1.0 MDA), and 0.955 (95% CI: 0.898–1.0 UTSW) in the cohorts analyzed separately (Fig. 3A, D, G, J). The combined calibration plot showed strong agreement between predicted and observed probabilities (Fig. 3O) and Brier scores showed strong predictive performance at 0.044.

Threshold-based analysis revealed that scores ≥30 had a positive predictive value (PPV) of 100% for AA across all validation cohorts (Fig. 3C, F, I, L). In contrast, scores below 0 were predominantly associated with IBMFS patients, yielding a PPV of 81.6% for IBMFS across all datasets. Analyzed separately, PPVs for IBMFS with PASS < 0 were 77.4% in RIME, 75.5% in NIH/USP, 94.0% in MDA, and 57.1% in UTSW, delineating a subset of individuals warranting further evaluation for IBMFS. Most, but not all, patients with intermediate scores (0–20) had AA: 85.7% across all cohorts, and individually—84.8% in RIME, 91.5% in NIH/USP, 66.7% in MDA, and 88.2% in UTSW (Fig. 3B, E, H, K, N). These findings may help identify patients who could benefit from IBMFS evaluation or re-evaluation, particularly in patients going to BMT and in cases of IST-refractory disease.

### The effect of TL and somatic alteration components on PASS performance

Because TL measurement by flow-FISH and somatic genetic testing results may be delayed or unavailable in some settings, we examined the effect of availability of these score components on PASS performance.

Among 928 patients, 457 (49.2%) had available TL measurements (Supplemental Table S11). In PASS, TL <1st percentile lowers the score, whereas TL ≥1st percentile or unavailable testing has no effect. Comparing within the subset of patients with available TL data, omission of TL shifted scores upward in patients with very short TL, increasing sensitivity for AA at PASS ≥ 30 but reducing sensitivity for IBMFS at PASS < 0 (Supplemental Figure S3). Despite this shift, discrimination remained strong without TL (ROC AUC 0.955) compared with PASS including TL (ROC AUC 0.974), with preserved calibration. PPV for AA at PASS ≥ 30 remained high without TL (98.8% vs. 100% with TL), while PPV for IBMFS at PASS < 0 was modestly increased (88.2% vs 83.8% with TL). In the full 928-patient cohort, PASS omitting TL had ROC AUC 0.9703, with 99.5% PPV for AA at PASS ≥ 30.

When TL was excluded, three of 181 IBMFS patients (1.7%) (all with TBD) had PASS ≥ 30 and were classified as AA (MDA074, NIH316, and USP022) (Supplemental Table S12). Detailed review demonstrated that in one patient NIH316, a notation of “abnormal cutaneous findings” was not included as IBMFS red flag due to insufficient descriptive detail in the published NIH/USP dataset. Prospective classification of this feature would have revised the no-TL PASS to the intermediate range (PASS = 20). Another patient (USP022) likely had concurrent AA in the setting of a germline TERT variant of uncertain significance. This patient presented with acute-onset NSAA, a 6% PNH clone, absence of IBMFS red flags, TL <1st percentile, and a TERT c.2154 C > A (p.Asp718Glu) variant of uncertain significance. The no-TL PASS was 30, whereas incorporation of TL appropriately reclassified the patient into intermediate category (PASS = 10), reflecting diagnostic uncertainty.

Somatic testing was available in 90.6% of patients (841/928) (Supplemental Table S13). Inclusion of the somatic component increased sensitivity for AA at PASS ≥ 30, while discrimination remained excellent whether the somatic component was included or omitted (ROC AUC 0.981 vs. 0.977, respectively)(Supplemental Figure S4). Omission of the somatic component led to modest redistribution of AA patients into lower and intermediate score ranges. PPV for AA at PASS ≥ 30 remained 100% regardless of somatic inclusion, whereas PPV for IBMFS at PASS < 0 was modestly reduced when somatic data were omitted.

A total of 419 patients (45.1% of 928) had evaluable TL and somatic data (Supplemental Table S14). Omission of both components resulted in a greater decline in discrimination than omission of either alone (ROC AUC 0.943 vs. 0.973 when both were included), though calibration remained good. Importantly, PASS retained high PPV for AA at PASS ≥ 30 (99.1%) even when both components were unavailable (Supplemental Fig. S5).

Together, these results demonstrate that TL and somatic components improve PASS precision and help resolve diagnostically ambiguous phenotypes, while PASS maintains robust discriminatory performance and excellent predictive value for AA when these data are unavailable.

### Comparison of PASS to prior diagnostic models

We next compared the diagnostic performance of PASS to NIH-ML [10] and RIME recursive partitioning models [51] (Table 3). We limited this analysis to patients with available PNH flow cytometry to ensure that patients could be analyzed using the RIME model. The NIH-ML model could be compared only for the NIH/USP cohort using published predictions from the NIH-ML manuscript [10], because the NIH-ML algorithm is not publicly available.

**Table 3:** Comparison of PASS to Prior Predictive Models.

	NIH-ML	RIME	PASS
	Gutierrez-Rodrigues et al.	Kaphan et al.	This publication (Aleixo et al.)
Model type	Machine learning (supervised classification)	Recursive partitioning decision tree	Point-based scoring system
Patient population	Combined pediatric and adult patients with BMF	Combined pediatric and adult patients with BMF	Adult patients with BMF
Variable number	26	3	7
Variables needed	(1) Age (2) Sex (3) Red Blood Cell Count (4) Hemoglobin Level (5) MCV (6) Platelet Count (7) Neutrophil Count (8) RDW (9) Lymphocyte Count (10) Monocyte Count (11) Eosinophil Count (12) Basophil Count (13) Reticulocyte Count (14) Telomere Length (Flow-FISH or Southern blot) (15) Bone Marrow Cellularity (16) Dysplasia or Increased Blasts (WHO MDS Criteria) (17) DC Mucocutaneous Clinical Triad (18) Isolated Mucocutaneous or Skin Findings (19) Physical Anomalies (20) Multiorgan Disease (21) History of Longstanding Cytopenia or Macrocytosis (22) History of Longstanding Bleeding or Recurrent Infections (23) Immunodeficiency (24) Immediate Family History of IBMFS (25) Extended Family History of IBMFS (26) Early Hair Graying	(1) Morphological abnormalities (2) PNH clone ( ≥ 0.1% on granulocytes) (3) Acute onset of BMF (cytopenia < 1 year)	(1) Cytopenia Severity (2) Acuity of Onset of Cytopenia (3) Age ( < 60 vs =60 years) (4) IBMFS Red Flag (5) Special Conditions Associated with Acquired Aplastic Anemia (6) Somatic Genetic Alterations (7) Lymphocyte Telomere Length (Flow-FISH)
Use of somatic findings	Not used	Required (PNH)	Included, but not required for model performance
Use of telomere length	Required for model performance	Not used	Included, but not required for model performance
Applicability/scoring coverage	Some cases are not issued a prediction (incomplete coverage)	Prediction for all cases	Prediction for all cases
Model output	Binary: Acquired or Inherited; some not able to be predicted	Binary: AA or IBMFS	Probability-based scoring: 1) Score ≥ 30: High probability AA 2) Scores 0-20: Probable, but not certain, AA. Consider further evaluation for IBMFS. 3) Score < 0: IBMFS is likely, further evaluation for IBMFS is required.
Algorithm transparency	Black-box model (coefficients and feature contributions are not known, requires software)	Yes (rule-based tree)	Yes (weighted score)
Bedside use	No. Requires use of online software.	Yes, 4 rules for classification based on 3 inputs.	Yes, point-based scoring.
Online tool availability	https://dir.nhlbi.nih.gov/DDxAA/	No calculator	https://pennmedicine.shinyapps.io/passcalc/
Calculator entries required	13 numeric entries, 77 selectable sub-inputs including detailed dysmorphology assessments	N/A	7 radio button inputs
**Direct comparison of score performance across cohorts (adult patients evaluable by all models)**			
PENN *n* = 185 (160 AA, 25 IBMFS)	N/A	ROC AUC 0.860 (0.783– 0.938) PPV for AA: 97.2% (141/145) PPV for IBMFS: 52.5% (21/40) Brier score = 0.080	ROC AUC 0.987 (0.975–0.999) PPV for AA ≥ 30: 100% (136/136) PPV for IBMFS < 0: 76.7% (23/30) PPV for AA 0–20: 89.5% (17/19) Brier score = 0.031
NIH/USP *n* = 238 (201 AA, 37 IBMFS)	ROC AUC 0.941 (0.889–0.992) PPV for AA: 98% 199/203 PPV for IBMFS: 94.3% (33/35) Brier score = 0.035 (4 IBMFS patients excluded as unclassifiable)	ROC AUC 0.864 (0.800– 0.928) PPV for AA: 96.8% (179/185) PPV for IBMFS: 58.5% (31/53) Brier score = 0.081	ROC AUC 0.970 (0.948 – 0.992) PPV for AA ≥ 30: 100% (147/147) PPV for IBMFS < 0: 75.0% (33/44) PPV for AA 0–20: 91.5% (43/47) Brier score = 0.043
RIME *n* = 256 (240 AA, 16 IBMFS)	N/A	ROC AUC 0.965 (0.948– 0.981) PPV for AA: 100.0% (223/223) PPV for IBMFS: 48.5% (16/33) Brier score = 0.037	ROC AUC 0.985 (0.972–0.998) PPV for AA ≥ 30: 100% (206/206) PPV for IBMFS < 0: 66.7% (14/21) PPV for AA 0–20: 93.1% (27/29) Brier score = 0.024
UTSW *n* = 73 (68 AA, 5 IBMFS)	N/A	ROC AUC 0.948 (0.912 – 0.984) PPV for AA: 100% (61/61) PPV for IBMFS: 41.7% (5/12) Brier score = 0.050	ROC AUC 0.956 (0.891–1.000) PPV for AA ≥ 30: 100% (53/53) PPV for IBMFS < 0: 60.0% (3/5) PPV for AA 0–20: 86.7% (13/15) Brier score = 0.030
MDA *n* = 113 (69 AA, 44 IBMFS)	N/A	ROC AUC 0.820 (0.745– 0.894) PPV for AA: 84.0% (63/75) PPV for IBMFS: 84.2% (32/38) Brier score = 0.160	ROC AUC 0.977 (0.952–1.000) PPV for AA ≥ 30: 100% (56/56) PPV for IBMFS < 0: 92.9% (39/42) PPV for AA 0–20: 66.7% (10/15) Brier score = 0.052
All Combined *n* = 865 (738 AA, 127 IBMFS)	N/A	ROC AUC 0.865 (0.830– 0.899) PPV for AA: 96.8% (667/689) PPV for IBMFS: 59.7% (105/176) Brier score = 0.075	ROC AUC 0.980 (0.972–0.988) PPV for AA ≥ 30: 100% (598/598) PPV for IBMFS < 0: 78.9% (112/142) PPV for AA 0–20: 88.0% (110/125) Brier score = 0.037
Model Comparison (DeLong’s χ² test)	PASS vs NIH-ML (NIH/USP) χ² = 1.07, *p* = 0.30	PASS vs RIME (all patients) χ² = 47.03, *p* < 0.001	

Abbreviations: *AA* aplastic anemia, *IBMFS* inherited bone marrow failure syndromes, *MDS* myelodysplastic neoplasm, *PNH* paroxysmal nocturnal hemoglobinuria, *MCV* mean corpuscular volume, *RDW* red cell distribution width, *Flow-FISH* flow cytometry–fluorescence in situ hybridization, *ROC AUC* area under the receiver operating characteristic curve, *PPV* positive predictive value, *WHO* World Health Organization, *DC* dyskeratosis congenita, *PENN* University of Pennsylvania cohort, *NIH/USP* National Institutes of Health/University of São Paulo cohort, *UTSW* University of Texas Southwestern cohort, *MDA* MD Anderson Cancer Center cohort *Brier score* measure of probabilistic prediction accuracy.

Comparing the three models in the NIH/USP cohort, both PASS and NIH-ML showed similarly excellent discriminatory performance (PASS 0.970 [95% CI 0.948–0.992], NIH-ML ROC of 0.941 [95% CI 0.889–0.992]. However, PASS had stronger PPV for AA: PASS PPV for AA was 100% (147/147) for PASS ≥ 30, whereas the NIH-HL model failed to issue a prediction for 4 IBMFS patients (9.1% of 44), and misclassified another 4 (9.1%) of IBMFS patients as AA (Supplemental Table S15).

PASS outperformed the RIME model in all except the RIME derivation cohort, where performance was comparable (Table 3). In all five cohorts combined (*N* = 865), PASS demonstrated ROC 0.980 (95% CI 0.972–0.988), PPV for AA ≥ 30 = 100% 598/598), and Brier 0.037, compared with ROC AUC 0.865 (0.830–0.899), PPV for AA 96.8% (667/689), and Brier 0.075. 22 of 127 (17.3%) of IBMFS patients were misclassified as AA by the RIME model (Supplemental Table S16), compared to 0 for patients with PASS ≥ 30.

### Inter-operator reproducibility and the development of the clinical calculator tool

To assess inter-operator reproducibility, three hematology-oncology clinicians independently applied the PASS to 20 randomly selected patients from the training cohort. Inter-observer agreement, measured using Fleiss’ κ (a statistic accounting for agreement expected by chance), was 0.875, indicating near-perfect agreement and good reproducibility across independent raters. Minor variability was observed in exact point totals, reflecting inherent limitations of retrospective clinical data abstraction; however, agreement on categorical diagnostic classification remained uniformly high, demonstrating the real-world robustness and flexibility of PASS scoring, with consistent assignment to AA, IBMFS, or intermediate categories across raters.

**Fig. 4:**
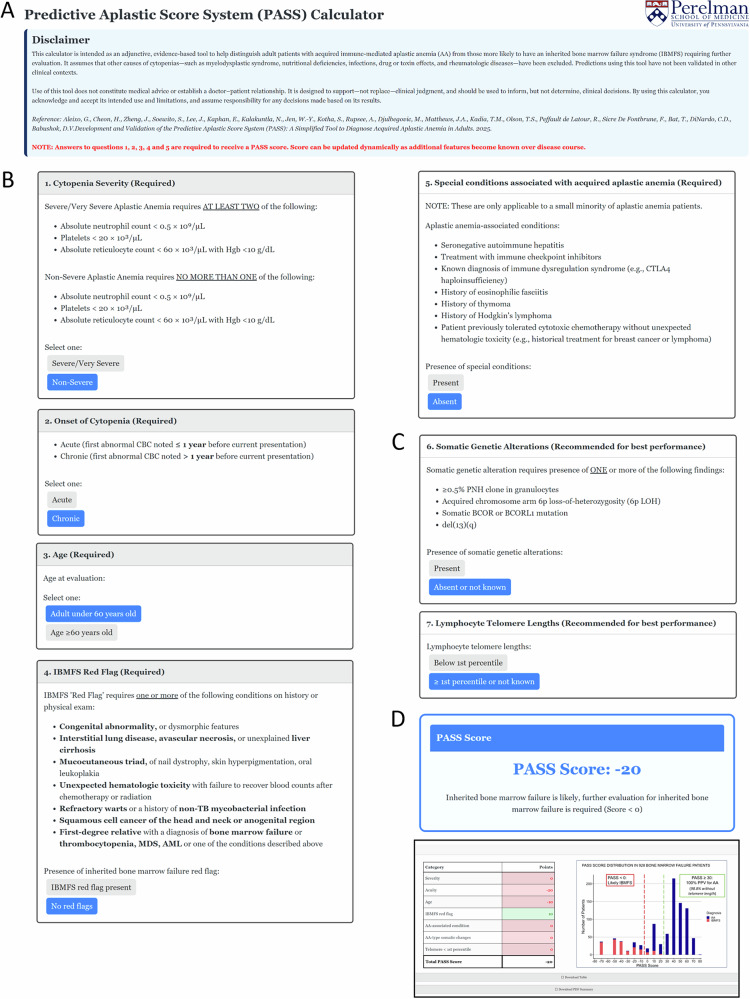
PASS score calculator. Shown are screenshots of the web-based PASS calculator, showing its use for a hypothetical patient case. **A** Calculator disclaimer**. B** Required clinical inputs that are typically available at the time of initial evaluation. **C** Score components for somatic genetic testing and telomere length assessment, which may not be immediately available and are not required for calculator use; however, inclusion of these components is recommended once available to optimize diagnostic performance (see text). **D** Calculator output displaying the total PASS score and corresponding diagnostic interpretation, along with a summary table of entered variables and assigned point values, and the distribution of PASS scores among 928 patients with bone marrow failure included in this study. The calculator is available at https://pennmedicine.shinyapps.io/passcalc/.

To support clinical implementation, we developed an open-access PASS clinical calculator (Fig. 4, available at https://pennmedicine.shinyapps.io/passcalc/). This tool allows users to input clinical variables and returns the calculated score along with its interpretation for three patient subgroups based on score thresholds ( ≥ 30, 0–20, and <0). The score can be recalculated as additional variables emerge during the disease course. To assist with interpretation, the tool also displays the corresponding score distributions for AA and IBMFS patients in the combined training and validation cohorts.

## Discussion

In this study, we employed a structured statistical approach in a training cohort of 212 and a validation cohort of 716 adult BMF patients to develop a clinical score that accurately identifies patients with classical features of AA and those at increased risk of harboring IBMFS. Although genetic testing has become more accessible and is increasingly incorporated into AA diagnostic workflows, evidence supporting the benefits of universal genetic testing in all adult BMF patients is lacking. Our results identify clinical factors that can distinguish AA and IBMFS in adults. We present a systematic framework that can be applied reliably to enable rapid diagnosis of 80% of AA patients with classical AA presentation and identification of individuals most likely to benefit from IBMFS testing. Validated across four independent cohorts, the PASS is a robust model that is accessible, efficient, and high yield, and particularly beneficial in settings without readily available genetic testing. To facilitate clinical implementation, we translated our PASS model into an open-access, web-based calculator.

Various features favoring the diagnosis of AA or IBMFS have been previously described [3–9, 11, 31, 52–55], but when considered in isolation they have only moderate discriminative value. Our findings confirm that IBMFS can present even in adults ≥60 years old, and that AA can sometimes follow a chronic or indolent course. Among patients with chronic non-severe cytopenias, 17% had AA, rising to 64% in the absence of IBMFS “red flags”. Conversely, IBMFS-associated features lacked sensitivity for IBMFS: 34% of IBMFS patients had normal TL, and 10% had no IBMFS “red flags”. Across cohorts, IBMFS accounted for up to ~20% of adult BMF cases, approximately two-thirds of which were telomere biology disorders (TBDs). These findings underscore the need for a validated diagnostic framework that can be readily applied in routine hematology practice.

PASS outperformed two previously published diagnostic models, the machine-learning (“NIH-ML”) model [10] and the Kaphan et al. recursive partitioning (RIME) model [51], in specificity for diagnosing AA (Table 3). A key distinction is that prior models were trained in mixed pediatric and adult cohorts, with IBMFS cases predominantly drawn from pediatric populations, where patients have a different spectrum of IBMFS. Conversely, PASS was developed specifically for adult BMF, a population that often lack overt dysmorphology and may instead present with adult-onset manifestations, such as pulmonary fibrosis in TBDs. NIH-ML [10] model demonstrated strong performance but misclassified or failed to generate predictions for 18.2% of adult IBMFS patients in the NIH/USP cohort. In addition, NIH-ML algorithm is not publicly available and its performance outside of NIH/USP cohort is not known. The model requires entry of >25 variables—including detailed dysmorphology assessments that are difficult to perform reliably outside pediatric subspecialty settings—and relies on TL for performance. The RIME model has a key strength in its simplicity and applicability to the real-world setting, requiring only three clinical variables for scoring. However, performance depends on correct implementation of four recursive decision-tree rules, without a validated bedside tool and showed reduced specificity outside its derivation cohort. When applied to our adult cohorts, it misclassified 17.3% of IBMFS patients as AA, particularly those with acute presentations lacking dysmorphology. PASS advances beyond prior models by combining superior discriminatory performance in adult cohorts with a transparent, bedside-applicable scoring algorithm that does not require TL testing and achieves near-perfect (100%) PPV for AA.

**Fig. 5:**
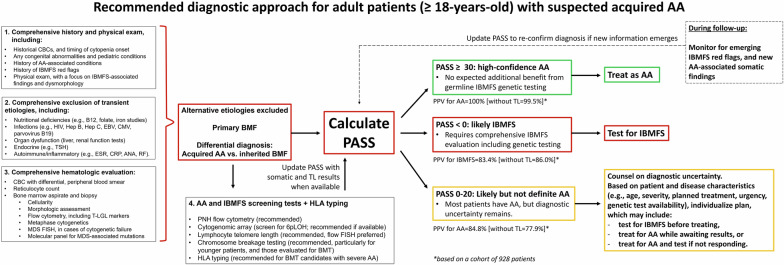
Schematic showing the recommended use of PASS in the diagnostic evaluation of suspected acquired aplastic anemia. Patients suspected of having aplastic anemia should undergo a comprehensive evaluation, as per standard guidelines. This should include evaluation of transient causes of cytopenias, and a bone marrow biopsy, with cytogenetics and molecular testing for acquired genetic alterations, and exclusion of MDS-defining abnormalities as per WHO classification criteria. We recommend PNH flow cytometry testing in all patients and find it helpful to screen for 6p LOH using cytogenomic arrays. We recommend screening for short telomeres by flow-FISH (where available), and Fanconi anemia with chromosome breakage studies (particularly, in younger adults and those anticipated to undergo bone marrow transplant). Although results of specialized testing may not be immediately available, patients can already be rapidly stratified by PASS using clinical criteria into one of three diagnostic categories: (1) high-confidence AA (PASS ≥ 30); (2) likely IBMFS (PASS < 0); and (3) an intermediate group (PASS 0–20) in whom AA is likely but not certain. Patients with PASS ≥ 30 have ~100% predictive accuracy for AA and can proceed with AA-directed therapy without delay with no added benefit to IBMFS genetic testing; the score should be recalculated over time as additional results are received and if any new IBMFS- or AA-associated somatic features emerge. In contrast, patients with scores <0 should undergo comprehensive IBMFS evaluation, including genetic testing. Patients with intermediate scores have greater diagnostic uncertainty, and management should be individualized; further IBMFS testing is appropriate (particularly if transplant is planned) but in some cases, depending on disease severity, planned therapy, and access to specialized genetic diagnostics, AA-directed treatment may be initiated with appropriate patient counseling either while awaiting results or with a plan to re-evaluate in case of non-response. CBC complete blood count, AA aplastic anemia, IBMFS inherited bone-marrow failure syndromes, HIV human immunodeficiency virus, Hep B hepatitis B virus, Hep C hepatitis C virus, EBV Epstein–Barr virus, CMV cytomegalovirus, TSH thyroid-stimulating hormone, ESR erythrocyte sedimentation rate, CRP C-reactive protein, ANA antinuclear antibody, RF rheumatoid factor, BM bone marrow, T-LGL large granular lymphocyte leukemia, FISH fluorescence in situ hybridization, MDS myelodysplastic neoplasm, PNH paroxysmal nocturnal hemoglobinuria, TL telomere length, BMT bone-marrow transplantation, 6pLOH copy-neutral loss of heterozygosity of chromosome arm 6p, PPV positive predictive value, HLA human leukocyte antigen.

We envision PASS as an adjunct to standard diagnostic evaluation for suspected AA (Fig. 5). Following exclusion of secondary causes and marrow assessment, patients can be rapidly stratified into three categories: high-confidence AA (PASS ≥ 30), likely IBMFS (PASS < 0), and an intermediate group (PASS 0–20). Patients with PASS ≥ 30 can proceed with AA-directed therapy without delay, whereas those with PASS < 0 should undergo comprehensive IBMFS evaluation, including genetic testing. Patients with intermediate scores have greater diagnostic uncertainty, and management should be individualized; further IBMFS testing is appropriate—particularly when transplantation is planned—but AA-directed therapy may be initiated in selected cases based on disease severity, treatment urgency, and access to genetic testing, with appropriate patient counseling and reassessment if response is not observed. Thus, PASS is intended to provide a systematic diagnostic framework for evaluation of adult AA patients; it is not intended to supplant clinical decision-making, as we recognize the nuances of clinical practice and the importance of clinical judgment.

This study has limitations. As a retrospective analysis, documentation of subtle IBMFS physical findings or family history may have been incomplete. However, independent manual chart review by two investigators and consistent performance across four external validation cohorts totaling 716 patients support real-world generalizability. The score’s reliance on multiple complementary features confers robustness to missing data, as demonstrated in sensitivity analyses excluding telomere and somatic components. Inter-rater agreement was high despite minor variability in component-level scoring. Our analysis focused on adults with BMF; telomere measurements were done by flow-FISH; future studies are needed to validate PASS with other telomere assays, in pediatric populations, and in prospective and community-based settings.

In conclusion, PASS is a practical and accurate clinical tool for distinguishing AA from IBMFS in adults, validated in 928 adult BMF patients across five independent international cohorts. By integrating routinely available clinical and laboratory data, PASS provides early diagnostic clarity, helps prioritize genetic testing, and offers a systematic framework to guide initial clinical management.

### Data sharing

All data tables supporting this study are provided in Supplemental Appendix.

## Supplementary information

Supplemental Appendix

## Data Availability

All data supporting the findings of this study are provided in the Supplemental Appendix. This includes de-identified datasets and PASS calculations for patients in the training cohort and the MDA and UTSW validation cohorts. Please see the original publications for primary data and model development of the NIH machine learning model [10] and RIME recursive partitioning model [51].
