# Supplementary material for: Development and validation of the predictive aplastic score system (PASS): a simplified tool to diagnose acquired aplastic anemia in adults
Source: Leukemia. 2026 Apr 27;40(7):1411–26. doi: 10.1038/s41375-026-02924-3 (PMC13323085; doi:10.1038/s41375-026-02924-3)
Supplement: Supplementary file 1 — Supplemental Appendix [file 41375_2026_2924_MOESM1_ESM.pdf]

Supplemental Appendix for

Manuscript Title: Development and Validation of the Predictive Aplastic Score System (PASS):

A Simplified Tool to Diagnose Acquired Aplastic Anemia in Adults

Authors: Gabriel Aleixo[1], HeeJin Cheon[2], Jiayin Zheng[3], Stephanie Soewito[4], Jimmy Lee[5], Eléonore Kaphan[6], Neha Kalakuntla[6], Wei-Ying Jen[4], Sumasri Kotha[8], Alex Rupsee[8], Mia Djulbegovic[1,8], Jairo A Matthews[4], Tapan M. Kadia[4], Timothy S. Olson[9, 10], Régis Peffault de Latour[6,11], Flore Sicre De Fontbrune[6], Taha Bat[5], Courtney D. DiNardo[4], Daria V. Babushok[1,10]

Affiliations:
[1] Division of Hematology-Oncology, Department of Medicine, University of Pennsylvania, Philadelphia, Pennsylvania
[2] Internal Medicine Residency Program, Department of Medicine, University of Pennsylvania, Philadelphia, Pennsylvania
[3] Department of Biostatistics, Epidemiology, and Informatics, Perelman School of Medicine, University of Pennsylvania, Philadelphia, Pennsylvania
[4] Department of Leukemia, Division of Cancer Medicine, The University of Texas MD Anderson Cancer Center, Houston, Texas
[5] Division of Hematology and Oncology, Department of Internal Medicine, UT Southwestern Medical Center, Dallas, Texas
[6]APHP, Service D'hématologie Greffe, Hôpital Saint-Louis, Paris, France
[7] School of Medicine, UT Southwestern Medical Center, Dallas, TX.
[8] Perelman School of Medicine, University of Pennsylvania, Philadelphia, Pennsylvania
[9] Division of Oncology, Department of Pediatrics, Children's Hospital of Philadelphia, Philadelphia, Pennsylvania
[10] Comprehensive Bone Marrow Failure Center, The Children's Hospital of Philadelphia, Philadelphia, Pennsylvania
[11] French Reference Center for AA & PNH, Inserm U1342, Saint Louis Research Institute, Team Translational Immunology in Immunotherapy and Hematology, Leukemia Institute Paris Saint Louis, Université Paris Cité, Paris, France

Corresponding Author:
Daria Babushok, M.D. Ph.D.
Division of Hematology-Oncology, Department of Medicine, Hospital of the University of Pennsylvania, Philadelphia, Pennsylvania
Room 808 BRB II/III, 421 Curie Blvd, Philadelphia, PA 19104
Email: daria.babushok@pennmedicine.upenn.edu
Phone: 215-614-1847, Fax: 215-615-5888

Table of Contents

Supplemental Methods 4
PNH Flow Cytometry 8
Acquired 6p CN-LOH Detection 8
Telomere length measurement 9
Online calculator 9
Supplemental Tables 10
Supplemental Table S1: Inherited Bone Marrow Failure Syndrome "Red Flags" 10
Supplemental Table S2. Acquired Aplastic Anemia-Associated Conditions 11
Supplemental Table S3. Functional and genetic testing performed for exclusion of IBMFS in the training cohort 12
Supplemental Table S4. Adjudication of acquired aplastic anemia diagnosis in the training cohort (n=162) 12
Supplemental Table S5: Characteristics of the 50 IBMFS patients in the training cohort. 13
Supplemental Table S6A: Characteristics of 44 IBMFS patients in the NIH/USP validation cohort 14
Supplemental Table S6B: Characteristics of 6 IBMFS patients in the UTSW validation cohort 14
Table S6C: Characteristics of 52 IBMFS patients in the MDA validation cohort 14
Table S6D: Characteristics of 29 IBMFS patients in the RIME validation cohort 15
Supplemental Table S7. Clinical Characteristics of the NIH/USP BMF Cohort 16
Supplemental Table S8. Clinical Characteristics of the UTSW BMF Cohort 17
Supplemental Table S9. Clinical Characteristics of the MDA BMF Cohort 18
Supplemental Table S10. Clinical Characteristics of the RIME BMF Cohort 19
Supplemental Table S11. The Effect of TL Availability on The Diagnostic Performance Of PASS 20
Supplemental Table S12. 3 IBMFS patients with PASS ≥ 30 when TL score component is omitted 20
Supplemental Table S13. The Effect of Somatic Genetic Testing Availability on the Diagnostic Performance of PASS 21
Supplemental Table S14. The Effect of the Availability of Both Telomere Lengths and Somatic Genetic Testing on the Diagnostic Performance of PASS 22
Supplemental Table S15: 8 NIH-USP IBMFS Patients Diagnosed as AA or Unable to be Classified by NIH-ML Model 23
Supplemental Table S16: 22 IBMFS Patients Predicted to Have AA by the RIME Model 24
Supplemental Figures 26

Supplemental Figure S1. Selection of NIH/USP cohort patient inclusion/exclusion 26
Supplemental Figure S2 The PASS model performance in the training cohort, calculated using the most sensitive PNH threshold and using all AA-associated somatic findings detected over the course of the disease. 28
Supplemental Figure S3 The effect of telomere length availability on PASS performance. 29
Supplemental Figure S4 The effect of somatic genetic test availability on PASS performance 30
Supplemental Figure S5 The effect of availability of both telomere length and somatic test components on PASS performance 31
Supplemental References 32
Supplemental Datasets 34
Supplemental Dataset S1. Training cohort individual-level clinical data 34
Supplemental Dataset S2. Training cohort individual-level PASS calculation. 44
Supplemental Dataset S3. Individual-level data and scoring for the NIH/USP cohort 51
Supplemental Dataset S4. UTSW cohort validation individual-level data and scoring ... 58
Supplemental Dataset S5. MD Anderson cohort validation individual-level data and scoring 62

Supplemental Methods

Patients and cohorts

The study was approved by the Institutional Review Boards (IRBs) of the participating institutions. Eligible subjects were adults (≥18 years) undergoing evaluation for BMF. BMF was defined as cytopenias with a hypocellular bone marrow in the absence of malignancy. All patients underwent comprehensive exclusion of secondary causes of cytopenias, including infections, nutritional deficiencies, rheumatologic conditions, medications, metabolic disease, and organ dysfunction. In this study, we included only those ultimately diagnosed with either AA or IBMFS. Most patients were initially evaluated in adulthood; however, adults with a history of chronic cytopenias who re-presented for BMF evaluation as adults were also eligible.

We identified the training cohort through an IRB-approved retrospective electronic medical record search of consecutive adult patients treated at the University of Pennsylvania (Penn) between 2010 and 2025 using ICD-9 and ICD-10 codes associated with AA, pancytopenia, and BMF. Additional adult patients cared for at Penn were included from the IRB-approved bi-institutional Penn and the Children's Hospital of Philadelphia (CHOP) BMF Registry cohort.

We performed external validation in four independent validation cohorts: adult patients from the published cohort from National Institutes of Health and the University of São Paulo (NIH/USP)[1] meeting inclusion criteria (Supplemental Figure S1), adult patients from the French centers participating in the French national reference center observatory nationwide database (RIME), and retrospective cohorts from MD Anderson (MDA) and University of Texas Southwestern (UTSW).

Diagnostic group assignment

Diagnostic adjudication was done in a standardized fashion across cohorts. IBMFS were diagnosed based on a combination of genetic testing and syndrome-specific assessments

following standard criteria[2,3]. AA was diagnosed using standard criteria, including evaluation of bone marrow cellularity, morphology, cytogenetics, followed by systematic exclusion of other disorders and etiologies that mimic AA[4-7]. Confirmation of AA diagnosis was sought via either 1) genetic and functional testing that excluded IBMFS, and/or 2) evidence of sustained response to IST at 6 months using NIH criteria[8], indicating immune-mediated bone marrow failure (i.e. AA). For patients who did not respond to IST or could not be evaluated for IST response (e.g., due to death or upfront transplantation) and who did not undergo IBMFS genetic testing, a diagnosis of presumed AA was established in accordance with standard AA diagnostic guidelines by each participating center, requiring exclusion of alternative etiologies by comprehensive clinical, pathologic, and laboratory evaluation and patient being managed clinically as having AA. The training cohort and the MDA and UTSW validation cohorts included both confirmed and presumed AA. All patients in the NIH/USP cohort had complete IBMFS genetic testing and telomere length (TL) measurements, and all AA patients in the RIME cohort had confirmed AA based on IBMFS genetic testing or evidence of IST.

Clinical data collection and scoring

Clinical and laboratory data were obtained through manual chart review of all patients, conducted independently by two study investigators (GA and DVB). Collected data included demographics; somatic genetic findings, including cytogenetics, next-generation sequencing, and cytogenomic array results; severity of cytopenias at diagnosis; presence and size of PNH clone; physical examination findings (e.g., congenital anomalies) and personal or family history of IBMFS-associated conditions (IBMFS “red flags”; Supplemental Table S1). Additional data included clinical conditions associated with AA (Supplemental Table S2)[9-15], and treatment history, such as IST (defined as anti-thymocyte globulin or cyclosporine) or BMT. Cytopenia severity was classified as severe/very severe (SAA/VSAA) or non-severe (NSAA) using the modified Camitta criteria[16,17]. Response was assessed according to NIH criteria[8].

PASS score development and statistical analysis

Clinically relevant variables in the training cohort were evaluated using univariate and multivariable logistic regression. We selected variables that were statistically significant in univariate analysis or those with perfect discrimination for inclusion in the PASS model using least absolute shrinkage and selection operator (LASSO) logistic regression[18]. We applied LASSO to the training cohort using 10-fold cross-validation to select the optimal penalization parameter λ that minimized the mean cross-validated binomial deviance (the minimum-deviance criterion). Variables with non-zero coefficients at the selected λ were retained in the final model.

The scoring scheme was derived from the direction and relative magnitude of the LASSO regression coefficients, with predictors grouped into discrete weight categories to reflect their proportional influence while preserving clinical interpretability and practical considerations for use at the bedside. Rather than applying raw coefficient scaling, which would yield non-integer and non-round values and require computational support, we employed simplified stepwise weighting (±10 and ±20) to maintain the relative contribution of each predictor in a format suitable for bedside clinical use. Variables that greatly influenced the score were given ±20 points, while those with lower impact on the score in the model (e.g., age, AA-associated clinical findings), were ±10 points. For patients with NSAA, we modified point values for chronic presentation and age <60 years to reflect higher baseline probability of IBMFS in this subgroup.

Validation was performed using patient-level data for all cohorts. Missing values were scored as 0 (i.e., imputed as an absence of an abnormal finding). This approach was primarily applied to missing somatic testing and TL measurements, whereas clinical data were available for all patients, with the exception of AA-associated clinical conditions in the published NIH/USP dataset. The timing of onset of cytopenias was assessed pragmatically: patients with historical blood counts dated >1 year prior to current presentation demonstrating one or more unexplained cytopenias without subsequent normalization were classified as chronic, while all

other cases—including those with normal blood counts within 1 year of presentation, patients without previous blood counts, and patients with transient and explained prior cytopenias (e.g. iron deficiency)—were classified as acute. We designed the PASS score to be dynamically updated as new clinical information emerges. For example, if TL or AA-associated somatic abnormalities are resulted later, the score can be recalculated.

PASS score performance and statistical analysis

The ability of the score to distinguish between AA and IBMFS was assessed using the area under the receiver operating characteristic curve (AUC-ROC)[19] and calibration plots comparing observed and predicted probabilities across deciles of risk. Additionally, the Brier score[20], which measures prediction accuracy as the average squared difference between predicted probabilities and actual outcomes, was calculated to quantitatively assess model performance. The positive predictive value (PPV), negative predictive value (NPV), sensitivity and specificity were calculated at different score thresholds. The association between PASS score and response to IST was tested using Fisher's exact test, with a two-tailed $p<0.05$ considered significant. Calibration goodness-of-fit was evaluated using the Hosmer–Lemeshow (HL) chi-square test, comparing observed and expected outcome frequencies across risk deciles.

To compare the predictive performance of PASS to other models, we calculated performance metrics for the Gutierrez-Rodrigues et al. machine-learning ("NIH-ML") model[1] using the NIH-ML predictions for the NIH/USP cohort published with the model. For the Kaphan et al. recursive partitioning (RIME) model, we applied four hierarchical decision rules to patients with the three required predictors as described[21]: (1) no morphological abnormalities and acute onset of BMF were classified as AA; (2) presence of morphological abnormalities with a PNH clone (≥0.1%) were classified as AA; (3) morphological abnormalities without a PNH clone were classified as IBMFS; and (4) no morphological abnormalities without acute BMF were classified

as IBMFS. Predictive performance was compared for same patients evaluated by the three models using ROC AUC, Brier scores, PPV for AA and IBMFS.

To evaluate the clinical utility and reproducibility of PASS, three hematology-oncology clinicians independently reviewed the medical records of 20 randomly selected patients and calculated PASS scores. To assess inter-rater reliability in diagnostic classification, we used Fleiss' kappa (κ), a standardized statistical measure of agreement among multiple raters, which accounts for the possibility of agreement on a given diagnosis occurring by chance.

All statistical analyses were performed using Stata version 18.0[22]. $p<0.05$ was considered statistically significant.

PNH Flow Cytometry

The presence of PNH clones was established by detection of GPI-anchored protein deficient granulocytes, monocytes, and erythrocytes using multicolor flow cytometry, as previously described[23,24]. PNH testing was performed as part of the patients' clinical evaluation by CLIA-certified flow cytometry laboratories at CHOP, the Hospital of the University of Pennsylvania (HUP), or sendout reference facilities in the course of routine clinical care. PNH clone size was determined as the percentage of GPI-anchor deficient granulocytes[24]. For PASS score calculation, we used a threshold of ≥0.5% granulocyte PNH clone unless otherwise specified.

Acquired 6p CN-LOH Detection

The presence of acquired 6p CN-LOH was ascertained by single nucleotide polymorphism array (SNP-A) analysis of DNA extracted from bone marrow or peripheral blood samples[25], as a part of the standard clinical evaluation by CLIA-certified cytogenomic testing facilities at the CHOP Division of Genomic Diagnostics or ARUP Laboratories. Internal regions of homozygosity, with B allele frequency of 1 or 0, were assumed to be constitutional, and were excluded from the determination of acquired 6p CN-LOH[26].

Telomere length measurement

Telomere lengths were measured as a part of clinical evaluation by flow fluorescence in situ hybridization (flow FISH) by one of two CLIA TL testing centers (Johns Hopkins University, Baltimore, MD or Repeat Diagnostics, Inc., North Vancouver, Canada). Telomere length plots were generated as previously described [27]. Briefly, we used R to plot telomere lengths in granulocytes and lymphocytes for patients with available telomere length numerical data against the telomere lengths of healthy individuals from the previously published studies[28,29]. The percentile curves for the 1st, 10th, 50th, 90th, and 99th percentiles of the healthy individuals were generated based on published data[28,29].

Online calculator

The online calculator was developed in R studio, as a Shiny application, similar to previously described clinical calculators[30]. Additional features added for our calculator were the downloadable, dated, PDF document detailing the score calculation and a downloadable score table. The calculator was deployed to the Shinyapps.io and is freely available online at https://pennmedicine.shinyapps.io/passcalc/.

Supplemental Tables

Supplemental Table S1: Inherited Bone Marrow Failure Syndrome "Red Flags"

	One or more of the following red flag conditions on history or physical exam:	Rationale:
**Inherited bone marrow failure "Red Flags"**	• Congenital abnormality, or dysmorphic features	Syndromic features suggestive of inherited condition
	• Interstitial lung disease, avascular necrosis, or unexplained liver cirrhosis	Clinical features of telomere biology disorder
	• Mucocutaneous triad, of nail dystrophy, skin hyperpigmentation, oral leukoplakia	
	• Unexpected hematologic toxicity with failure to recover blood counts after chemotherapy or radiation	Suggestive of underlying bone marrow dysfunction, typical of classical IBMFS
	• Refractory warts or a history of non-TB mycobacterial infection	Suggestive of GATA2 deficiency
	• Squamous cell cancer of the head and neck or anogenital region	Suggestive of Fanconi anemia or telomere biology disorder
	• First-degree relative with a diagnosis of bone marrow failure or thrombocytopenia, MDS, AML or one of the conditions described above	Suggestive of inherited BMF or MDS predisposition syndrome

Supplemental Table S2. Acquired Aplastic Anemia-Associated Conditions

Acquired Aplastic Anemia Associated Condition	Selected References
Seronegative autoimmune hepatitis	• Oriol, A., J. M. Ribera, A. Hernandez, V. Soriano, F. Milla and E. Feliu (1994). "Aplastic anemia after non-A, non-B, and non-C hepatitis." Haematologica 79(2): 168-169.<br>• Levy, R. N., A. Sawitsky, A. L. Florman and E. Rubin (1965). "Fatal aplastic anemia after hepatitis. Report of five cases." N Engl J Med 273(21): 1118-1123.
Treatment with immune checkpoint inhibitors	• Dasari, S., W. Tse and J. Wang (2023). "Real-world evidence of incidence and outcomes of aplastic anaemia following administration of immune checkpoint inhibitors." Br J Haematol 202(6): 1205-1208.
Known diagnosis of immune dysregulation syndrome (e.g., CTLA4 haploinsufficiency)	• Solhaug, T. S., G. E. Tjonnfjord, K. Bjorgo and O. Kildahl-Andersen (2022). "A family with cytotoxic T-lymphocyte-associated protein 4 haploinsufficiency presenting with aplastic anaemia." BMJ Case Rep 15(2).
History of eosinophilic fasciitis	• de Masson, A., J. D. Bouaziz, R. P. de Latour, Y. Benhamou, C. Molucon-Chabrot, J. O. Bay, A. Laquerriere, J. M. Picquenot, D. Michonneau, V. Leguy-Seguin, M. Rybojad, B. Bonnotte, F. Jardin, H. Levesque, M. Bagot and G. Socie (2013). "Severe aplastic anemia associated with eosinophilic fasciitis: report of 4 cases and review of the literature." Medicine (Baltimore) 92(2): 69-81.
History of thymoma	• Gendron, N., F. S. de Fontbrune, A. Guyard, J. Fadlallah, S. Chantepie, M. D'Aveni, R. Le Calloch, A. Garnier, M. A. Couturier, V. Morel, C. Bernard, L. Terriou, E. Lazaro, G. Socie and R. P. de Latour (2020). "Aplastic anemia related to thymoma: a survey on behalf of the French reference center of aplastic anemia and a review of the literature." Haematologica 105(7): e333-e336.
History of Hodgkin's lymphoma	• Linaburg, T., A. R. Davis, N. V. Frey, M. R. Khawaja, D. J. Landsburg, S. J. Schuster, J. Svoboda, Y. Li, Y. Borovskiy, T. S. Olson, A. Bagg, E. O. Hexner and D. V. Babushok (2019). "Hodgkin lymphoma patients have an increased incidence of idiopathic acquired aplastic anemia."<br>• Rovo, A., A. Kulasekararaj, M. Medinger, P. Chevallier, J. M. Ribera, R. Peffault de Latour, C. Knol, S. Iacobelli, E. Kanfer, B. Bruno, S. Maury, P. Quarello, M. B. C. Koh, H. Schouten, I. W. Blau, A. Tichelli, A. Hill, A. Risitano, J. Passweg, J. Marsh, P. Dreger, C. Dufour and E. Severe Aplastic Anaemia Working Party of the (2019). "Association of aplastic anaemia and lymphoma: a report from the severe aplastic anaemia working party of the European Society of Blood and Bone Marrow Transplantation." Br J Haematol 184(2): 294-298. PLoS One 14(4): e0215021
Patient previously tolerated cytotoxic chemotherapy without unexpected hematologic toxicity (e.g., historical treatment for breast cancer or lymphoma)	Absence of unexpected hematologic toxicity argues against an underlying IBMFS and favors a newly developed acquired bone marrow failure (AA).<br><br>***Note that therapy-related MDS and other malignancies were excluded.***

Supplemental Table S3. Functional and genetic testing performed for exclusion of IBMFS in the training cohort

Type of IBMFS Testing		Total Patients (n=212)	Aplastic Anemia (n=162)	Inherited BMF (n=50)
Telomere length by flow-FISH		116 (54.7%)	77 (47.5%)	39 (78.0%)
Chromosome breakage testing for FA		90 (42.5%)	67 (41.4%)	23 (46.0%)
IBMFS genetic testing	Performed	65 (30.7%)	17 (10.5%)	48 (96.0%)
	Not performed	147 (69.3%)	145 (89.5%)	2 (4.0%)

Supplemental Table S4. Adjudication of acquired aplastic anemia diagnosis in the training cohort (n=162)

IBMFS Evaluation	Characteristic	N (% evaluable)		
Chromosome Breakage Studies	Normal	67 (100.0%)		
	Abnormal	0 (0.0%)		
	NA	95		
Lymphocyte Telomere Lengths	Normal (>10th percentile)	55 (71.4%)		
	1-10th percentile	20 (26.0%)		
	<1st percentile	2 (2.6%)		
	NA	85		
Genetic testing for IBMFS	Negative genetic testing for IBMFS	17 (10.5%)		
	No genetic testing	145 (89.5%)		
Response to IST	PR or CR at 6 months	120 (85.7%)		
	Refractory	20 (14.3%)	- 4 had negative IBMFS genetic test	
			- 16 had no IBMFS genetic testing	- 8 Responded to IST after 6 months
				- 4 Refractory (died)
				- 4 Refractory – received BMT
	Not Evaluable	22	- 11 Did not receive IST	- 3 had negative IBMFS genetic test
				- 3 had NSAA with PNH clone
				- 5 went to upfront BMT
			- 11 Received IST, but died, was transplanted or was lost to follow-up before 6 months	- 3 had negative IBMFS genetic testing
				- 1 Responded to IST after 6 months
				- 5 died after IST
				- 2 received BMT

Dark green shading indicates patients with a confirmed diagnosis of AA, based on hematologic response to IST at 6 months and/or negative IBMFS genetic testing. Light green shading denotes patients with a presumed diagnosis of AA, established through expert clinical assessment.

Supplemental Table S5: Characteristics of the 50 IBMFS patients in the training cohort.

**Clinical Characteristic**		**Patients (%)**
**Type of IBMFS**	Telomere Biology Disorder	25 (50.0%)
	Fanconi Anemia	8 (16.0%)
	Diamond Blackfan Anemia	5 (10.0%)
	GATA2 Deficiency	3 (6.0%)
	Other/BMF NOS*	9 (18.0%)
**Telomere Length Testing**	Normal (>10th percentile)	7 (17.9%)
	1-10th percentile	10 (25.6%)
	<1st percentile	22 (56.4%)
	NA	11
**Clinical history of IBMFS "red flag" findings (see Table S1)**	Yes	44 (88.0%)
	No	6 (12.0%)
	Total	50

* Others including Kabuki Syndrome (n=1), Ghosal hematodiaphyseal dysplasia (n=1), TUBB-mutated Complex cortical dysplasia with other brain malformations (n=1), GATA1-thrombocytopenia (n=1), SDS (n=1), SON (n=1), WAS (n=1), and IBMFS-NOS (clinical diagnosis of IBMFS not otherwise specified without an established genetic diagnosis), n=2.

Supplemental Table S6A: Characteristics of 44 IBMFS patients in the NIH/USP validation cohort

Type of IBMFS	Total (n=44)	TBD (n=41)	FA (n=2)	SDS (n=1)
Age, median (range)	29 (18-59)	29 (18-59)	42.5 (26-59)	24
Lymphocyte telomere lengths				
Normal	2 (4.5%)	0 (0.0%)	1 (50.0%)	1 (100.0%)
1-10th	2 (4.5%)	2 (4.9%)	0 (0.0%)	0
<1st	40 (90.9%)	39 (95.1%)	1 (50.0%)	0
Genetic cause known	43 (97.7%)	40 (97.6%)	2 (100.0%)	1 (100.0%)
Genetic cause not identified	1 (2.3%)	1 (2.4%)	0	0

Supplemental Table S6B: Characteristics of 6 IBMFS patients in the UTSW validation cohort

Type of IBMFS	TBD (n=6)
Age, median (range)	41 (31-65)
Lymphocyte telomere lengths	
Normal	0
1-10th	3 (50%)
<1st	3 (50%)
Genetic cause known	6 (100%)
Genetic cause not identified	0

Table S6C: Characteristics of 52 IBMFS patients in the MDA validation cohort

Type of IBMFS	Total (n=52)	TBD (n=26)	GATA2 deficiency (n=7)	FA (n=6)
Age, median (range)	37 (1-78)	46 (5-78)		29.5 (1-41)
Lymphocyte telomere lengths				
Normal	0	0	0	0
1-10th	0	0	0	0
<1st	25 (48.1%)	25 (96.1%)	0	0
NA	14	1 (3.9%)	7 (100%)	6 (100%)
Genetic cause known	52 (100%)	26 (100%)	7 (100%)	2 (33.3%)
Genetic cause not identified	0	0	0	4 (chromosome breakage positive)

Other IBMFS, not individually tabulated were: DBA (n=4), Bloom syndrome (n=1), Li Fraumeni Syndrome (n=1), ANKRD26 (n=1), and Noonan (CBL, n=1).

Table S6D: Characteristics of 29 IBMFS patients in the RIME validation cohort

Type of IBMFS	Total (n=29)	TBD (n=19)	GATA2 deficiency (n=1)	FA (n=5)	other (n=5)
**Age, median (range)**	36.6 (18.0–71.9)	40.7 (21.3–71.9)	46.6 (46.6–46.6)	32.1 (18.0–41.8)	38.3 (21.0–66.9)
**Lymphocyte telomere lengths**					
**Normal**	3 (17.6%)	1 (7.7%)	1 (100.0%)	1 (50.0%)	0 (0.0%)
**1-10th**	2 (11.8%)	1 (7.7%)	0 (0.0%)	1 (50.0%)	0 (0.0%)
**<1st**	12 (70.6%)	11 (84.6%)	0 (0.0%)	0 (0.0%)	1 (100.0%)
**NA**	12	6	0	3	3
**Genetic cause known**	100%				
IBMFS diagnosis required one or more pathogenic constitutional variants in genes known to cause IBMF were identified or if the chromosomal breakage test was diagnostic for Fanconi anemia					

Supplemental Table S7. Clinical Characteristics of the NIH/USP BMF Cohort

Patient Characteristic		Patients (n=247)	Aplastic Anemia (n=203)	Inherited BMF (n=44)	OR	95% Confidence Interval	P-value
Sex	M, n (%)	127 (51.4%)	100 (49.3%)	27 (61.4%)			
	F, n (%)	120 (48.6%)	103 (50.7%)	17 (38.6%)			
Age at diagnosis	Medan, years (range)	38 (18-86)	40 (18-86)	29 (18-59)			
	Age ≥ 60 years, n (%)	42 (17.0%)	42 (20.7%)	0 (0.0%)	23.421[1]	1.413-388.164	**0.002**
	Age < 60 years, n (%)	205 (83.0%)	161 (79.3%)	44 (100.0%)			
Cytopenia severity, n (%)	NSAA	102 (41.3%)	62 (30.5%)	40 (90.9%)	22.742[2]	7.798-66.325	**<0.001**
	SAA/VSAA	145 (58.7%)	141 (69.5%)	4 (9.1%)			
Chronicity of cytopenia, n (%)	Acute	223 (90.3%)	199 (98.0%)	24 (54.5%)	41.458[3]	13.074-131.466	**<0.001**
	Longstanding cytopenias or macrocytosis	24 (9.7%)	4 (2.0%)	20 (45.5%)			
Lymphocyte telomere lengths	Normal	152 (61.5%)	150 (73.9%)	2 (4.5%)	0.010[4]	0.003 - 0.032	**<0.001**
	1-10th	36 (14.6%)	34 (16.7%)	2 (4.5%)			
	<1st	59 (23.9%)	19 (9.4%)	40 (90.9%)			
Karyotype	Normal	195 (93.3%)	161 (92.5%)	34 (97.1%)			
	Abnormal	10 (4.8%)	9 (5.2%)	1 (2.9%)			
	Chromosome 7 or complex	4 (1.9%)	4 (2.3%)	0 (0.0%)			
	NA	38	29	9			
IBMF Red Flag	Yes	35 (14.2%)	12 (5.9%)	23 (52.3%)	0.057[5]	0.025-0.132	**<0.001**
	DC mucocutaneous triad	12 (4.9%)	0 (0.0%)	12 (27.3%)			
	Physical anomalies	9 (3.6%)	5 (2.5%)	4 (9.1%)			
	Immediate family members with similar phenotype	18 (7.3%)	7 (3.4%)	11 (25.0%)			
	No	212 (85.8%)	191 (94.1%)	21 (47.7%)			
PNH clone, patients, n (%)	None (<1%)	182 (77.1%)	142 (72.8%)	40 (97.6%)	14.939[6]	2.002-111.343	**<0.001**
	≥1%	34 (14.4%)	33 (16.9%)	1 (2.4%)			
	≥10%	20 (8.5%)	20 (10.3%)	0 (0.0%)			
	NA	11	8	3			

Our study inclusion criteria were applied to the published dataset from Gutierrez-Rodrigues et al: Differential diagnosis of bone marrow failure syndromes guided by machine learning. Blood 141:2100-2113, 2023, as shown in Supplemental Figure 1, resulting in 247 patients with AA and IBMFS that were used as one of the validation cohorts for the PASS score. Green shading indicates factors associated with the diagnosis of AA, and salmon shading indicates factors associated with the diagnosis of IBMFS. [1]Odds Ratio (OR) for Age ≥ 60 years in AA vs. IBMFS; [2]OR for SAA/VSAA in AA vs. IBMFS; [3]OR for acute onset cytopenias in AA vs IBMFS; [4]OR for lymphocyte telomere lengths <1st percentile in AA vs. IBMFS; [5]OR for presence of any of the listed IBMFS red flags in AA vs IBMFS; [6]OR for PNH clone >1% in AA vs IBMFS. Bold indicates statistically significant p-value.

Supplemental Table S8. Clinical Characteristics of the UTSW BMF Cohort

Patient Characteristic		Total Patients (n=78)	Aplastic Anemia (n=72)	Inherited BMF (n=6)	OR	95% Confidence Interval	P-value
Age at diagnosis	Median, years (range)	49 (18-81)	49.5 (18-81)	41 (31-65)			
	Age ≥ 60 years, n (%)	49 (62.8%)	27 (37.5%)	2 (33.3%)	1.200[1]	0.264 - 6.624	1.000
	Age < 60 years, n (%)	29 (37.2%)	45 (62.5%)	4 (66.7%)			
Cytopenia severity	NSAA	33 (42.4%)	28 (38.8 %)	5 (83.3%)	7.857[2]	0.871 – 70.81	0.06
	SAA/VSAA	45 (57.6%)	44 (61.1%)	1 (16.7%)			
Chronicity of cytopenia	Acute (1 year or less)	66 (84.6%)	64 (88.9%)	2 (33.3%)	16.000[3]	3.041 - 88.390	**0.004**
	Chronic (>1 year)	12 (15.4%)	8 (11.1%)	4 (66.6%)			
Lymphocyte telomere lengths	Normal	3 (27.3)	3 (60.0%)	0	0.000[4]	0.000 - 1.260	0.182
	1-10th	5 (55.6%)	2 (40.0%)	3 (50.0%)			
	<1st	3 (27.3%)	0	3 (50.0%)			
	NA	67	67	0			
AA-associated clinical findings	Present	5 (7.4%)	4 (5.5%)	1 (16.7%)	0.294	0.031 - 4.260	0.337
	Absent	73 (93.6%)	68 (94.4%)	5 (83.3%)			
IBMF red flag	Yes	16 (20.5%)	11 (15.3%)	5 (83.3%)	0.036[5]	0.003 - 0.338	**0.001**
	No	62 (79.5%)	61 (84.7%)	1 (16.7%)			
PNH clone	None (<0.5%)	35 (57.4%)	30 (52.6%)	5 (83.3%)	+infinity[6]	1.093 - infinity	0.068
	≥0.5% and < 10%	21 (34.4%)	21 (36.8%)	0			
	≥10%	5 (8.2%)	6 (10.5%)	0			
	NA	16	15	1			
Acquired 6pLOH	NA	78 (100%)	72 (100%)	6 (100%)			
BCOR or BCORL1 somatic mutation	Yes	2 (5.6%)	2 (6.1%)	0	+infinity	0.038 - infinity	1.000
	No	34 (94.4%)	31 (93.9%)	3 (100%)			
	NA	42	39	3			
Del(13)(q)	Yes	2 (3.1%)	2 (3.4%)	0	+infinity	0.037 - infinity	1.000
	No	62 (96.9%)	57 (96.6%)	5 (100%)			
	NA	14	13	1			

Age at diagnosis, cytopenia severity, chronicity of cytopenia, lymphocyte telomere lengths, AA-associated clinical findings, IBMF red flags, and PNH clone distribution are shown, along with data on acquired 6pLOH, BCOR/BCORL1 somatic mutations, and del(13q). Odds Ratio, OR. Bold indicates statistically significant p-values. Green shading indicates factors associated with the diagnosis of AA, and salmon shading indicates factors associated with the diagnosis of IBMFS. [1]Odds Ratio (OR) for Age ≥ 60 years in AA vs. IBMFS; [2]OR for SAA/VSAA in AA vs. IBMFS; [3]OR for acute onset cytopenias in AA vs IBMFS; [4]OR for lymphocyte telomere lengths <1st percentile in AA vs. IBMFS; [5]OR for presence of any of the listed IBMFS red flags in AA vs IBMFS; [6]OR for PNH clone ≥0.5% in AA vs IBMFS.

Supplemental Table S9. Clinical Characteristics of the MDA BMF Cohort

Patient Characteristic	Characteristic	Total Patients (n=121)	Aplastic Anemia (n=69)	Inherited BMF (n=52)	OR	95% Confidence Interval	P-value
Age at diagnosis	Median, years (range)	33 (1-78)	30 (18-59)	37 (1-78)			
	Age ≥ 60 years, n (%)	6 (5.0%)	0	7 (13.4%)	0.000[1]	0.000 - 0.511	**0.005**
	Age < 60 years, n (%)	115 (95.0%)	69 (100%)	45 (86.6%)			
Cytopenia severity	NSAA	60 (49.6%)	11 (15.9%)	49 (94.2%)	86.120[2]	22.650 - 282.300	**<0.001**
	SAA/VSAA	61 (50.4%)	58 (84.1%)	3 (5.8%)			
Chronicity of cytopenia	Acute (1 year or less)	80 (66.1%)	63 (91.3%)	17 (32.7%)	21.620[3]	7.800 - 59.320	**<0.001**
	Chronic (>1 year)	41 (13.9%)	6 (8.7%)	35 (67.3%)			
Lymphocyte telomere lengths	Normal	10 (25.6%)	10 (76.9%)	0	0.015[4]	0.002-0.1231	**<0.001**
	1-10th	2 (5.1%)	2 (15.4%)	0			
	<1st	25 (69.2%)	1(7.7%)	25 (100%)			
	NA	83	56	27			
AA-associated clinical findings	Present	6 (5.0%)	4 (5.8%)	2 (3.8%)	1.539	0.271-8.739	0.699
	Absent	115 (95.0%)	65 (94.2%)	50 (96.2%)			
IBMF red flag	Yes	46 (38.0%)	4 (5.8%)	42 (80.8%)	0.015[5]	0.004-0.050	**<0.001**
	No	75 (62.0%)	65 (94.2%)	10 (19.2%)			
PNH clone	None (<0.5%)	60 (69.8%)	35 (57.4%)	25 (100%)	Infinity[6]	4.567 - infinity	**<0.001**
	≥0.5% and < 10%	16 (18.6%)	16 (26.2%)	0			
	≥10%	10 (11.6%)	10 (16.4%)	0			
	NA	35	8	27			
BCOR or BCORL1 somatic mutation	Yes	7 (25.0%)	3 (21.4%)	3 (21.4%)	1.000	0.164-6.083	1.000
	No	21 (75.0%)	11 (78.6%)	11 (78.6%)			
	NA	93	55	38			
6pLOH	NA	121 (100%)	69 (100%)	52 (100%)			
Del(13)(q)	Yes	0	0	0			
	No	110 (100%)	61 (100%)	49 (100%)			
	NA	11	8	3			

Age at diagnosis, cytopenia severity, chronicity of cytopenia, lymphocyte telomere lengths, AA-associated clinical findings, IBMF red flags, and PNH clone distribution are shown, along with data on acquired 6pLOH, BCOR/BCORL1 somatic mutations, and del(13q). Odds Ratio, OR. Bold indicates statistically significant p-values. Green shading indicates factors associated with the diagnosis of AA, and salmon shading indicates factors associated with the diagnosis of IBMFS. [1]Odds Ratio (OR) for Age ≥ 60 years in AA vs. IBMFS; [2]OR for SAA/VSAA in AA vs. IBMFS; [3]OR for acute onset cytopenias in AA vs IBMFS; [4]OR for lymphocyte telomere lengths <1st percentile in AA vs. IBMFS; [5]OR for presence of any of the listed IBMFS red flags in AA vs IBMFS; [6]OR for PNH clone ≥0.5% in AA vs IBMFS.

Supplemental Table S10. Clinical Characteristics of the RIME BMF Cohort

Patient Characteristic	Characteristic	Total Patients (n=270)	Aplastic Anemia (n=241)	Inherited BMF (n=29)	OR	95% Confidence Interval	P-value
Age at diagnosis	Median, years (range)	48.3 (18.0–91.6)	51.4 (18.0–91.6)	36.6 (18.0–71.9)			
	Age ≥ 60 years, n (%)	91 (33.7%)	88 (36.5%)	3 (10.3%)	4.985[1]	1.538- 15.980	**0.004**
	Age < 60 years, n (%)	179 (66.3%)	153 (63.5%)	26 (89.7%)			
Cytopenia severity, n (%)	NSAA	58 (21.5%)	38 (15.8%)	20 (69.0%)	11.870[2]	4.901 - 27.700	**<0.001**
	SAA/VSAA	212 (78.5%)	203 (84.2%)	9 (31.0%)			
Chronicity of cytopenia, n (%)	Acute (1 year or less, or unknown)	243(90.0%)	236 (97.9%)	7 (24.1%)	148.3[3]	41.120 - 478.100	**<0.001**
	Chronic (>1 year)	27 (10.0%)	5 (2.1%)	22 (75.9%)			
Median lymphocyte telomere lengths	Normal	21 (46.7%)	18 (64.3%)	3 (17.6%)	0.197[4]	0.054 - 0.701	**0.016**
	1-10th	3 (6.7%)	1 (3.6%)	2 (11.8%)			
	<1st	21 (46.7%)	9 (32.1%)	12 (70.6%)			
	NA	225	213	12			
Findings associated with AA	Present	9 (3.3%)	9 (3.7%)	0 (0.0%)	+infinity	0.100 - +infinity	0.600
	Absent	261 (96.7%)	232 (96.3%)	29 (100.0%)			
Morphologic abnormalities	Yes	38 (14.2%)	14 (5.9%)	24 (82.8%)	0.013[5]	0.004 - 0.039	**<0.001**
	Yes, without description	2 (0.7%)	2 (0.8%)	0 (0.0%)			
	No	228 (85.1%)	223 (93.3%)	5 (17.2%)			
	NA	2	0	2			
PNH clone, patients, n (%)	None (0%)	117 (50.6%)	102 (54.2%)	15 (100.0%)	+infinity[6]	2.833 - +infinity	**<0.001**
	>0% to 0.5%	31 (12.4%)	31 (14.4%)	0			
	≥0.5% and < 10%	68 (29.4%)	68 (31.5%)	0			
	≥10%	15 (6.5%)	15 (6.9%)	0			
	NA	39	25	14			

Odds Ratio, OR. Bold indicates statistically significant p-values. Green shading indicates factors associated with the diagnosis of AA, and salmon shading indicates factors associated with the diagnosis of IBMFS. [1]Odds Ratio (OR) for Age ≥ 60 years in AA vs. IBMFS; [2]OR for SAA/VSAA in AA vs. IBMFS; [3]OR for acute onset cytopenias in AA vs IBMFS; [4]OR for lymphocyte telomere lengths <1st percentile in AA vs. IBMFS; [5]OR for presence of any morphologic abnormalities in AA vs IBMFS; we conservatively grouped entries without description of abnormalities as no abnormalities; [6]OR for PNH clone ≥0.5% in AA vs IBMFS. 6pLOH, BCOR, and del(13)(q) were not available.

Supplemental Table S11. The Effect of TL Availability on The Diagnostic Performance Of PASS

Cohort	Total Patients	Patients with available TL test[1]	AA	IBMFS	PASS score with TL: ROC (95% CI)	PASS score with TL: PPV for AA at PASS ≥30	PASS score with TL: PPV for IBMFS at PASS <0	PASS score with TL: PPV for AA at PASS 0–20	PASS score omitting TL: ROC (95% CI)	PASS score omitting TL: PPV for AA at PASS ≥30	PASS score omitting TL: PPV for IBMFS at PASS <0	PASS score omitting TL: PPV for AA at PASS 0–20
**Training cohort (Penn)**	**212**	116 (54.7%)	77	39	0.985 (0.971–1.000)	55/55 (100%)	36/41 (87.8%	17/20 (85.0%)	0.981 (0.963–0.998)	57/57 (100%)	34/39 (87.2%)	15/20 (75.0%)
**All validation cohorts**	**716**	341 (47.6%)	249	92	0.969 (0.952–0.985)	176/176 (100%)	83/101 (82.2%)	55/64 (85.9%)	0.945 (0.921-0.968)	189/192 (98.4%)	71/80 (88.8%)	51/69 (73.9%)
**UTSW**	**78**	11 (14.1%)	5	6	1.000 (1.000–1.000)	5/5 (100%)	4/4 (100%)	0/2 (0%)	1.000 (1.000–1.000)	5/5 (100%)	4/4 (100%)	0/2 (0%)
**MDA**	**121**	38 (31.4%)	13	25	0.934 (0.841–1.000)	7/7 (100%)	24/26 (92.3%)	4/5 (80%)	0.884 (0.747-1.000)	8/9 (88.9%)	20/22 (90.9%)	3/7 (42.9%)
**NIH/USP**	**247**	247 (100%)	203	44	0.969 (0.948–0.990)	147/147(100%)	40/53 (75.5%)	43/47 (91.5%)	0.937 (0.904–0.971)	153/155 (98.7%)	33/39 (84.6%)	44/53 (83.0%)
**RIME**	**270**	45 (16.7%)	28	17	0.974 (0.939–1.000)	17/17 (100%)	15/18 (83.3%)	8/10 (80%)	0.968 (0.928-1.000)	23/23 (100%)	14/15 (93.3%)	4/7 (57.1%)
**All patients combined**	**928**	457 (49.2%)	326	131	0.974 (0.961-0.986)	231/231 (100%)	119/142 (83.8%)	72/84 (85.7%)	0.955 (0.937–0.973)	246/249 (98.8%)	105/119 (88.2%)	66/89 (74.2%)
**Performance metrics[2]**					Brier score = 0.054	Hosmer–Lemeshow (HL) χ²(degrees of freedom, df)= 8.02 (9), p=0.330			Brier score = 0.069	HL χ²(df) = 6.38 (8), p=0.383		

[1] Performance metrics for PASS with telomere testing were calculated only among patients with available telomere length measurements; denominators therefore differ from the full cohort. Percentages exclude missing data. [2]Calibratoni performance metrics calculated for all patients combined.

Supplemental Table S12. 3 IBMFS patients with PASS ≥ 30 when TL score component is omitted.

Study ID	True Diagnosis	Diagnosis detail	PASS including TL component	PASS omitting TL component	IBMFS red flag	Acuity	Severity	PNH	TL
MDA074	IBMFS	TBD	20	40	No red flags	Acute	SAA/VSAA	PNH Negative (<0.1%)	Short (<1)
NIH316	IBMFS	TBD (TERT)	20	40	No red flags	Acute	SAA/VSAA	PNH Negative (<0.1%)	Short (<1)
USP022	IBMFS	TBD (TERT)	10	30	No red flags	Acute	NSAA	PNH Positive (6% clone)	Short (<1)

Supplemental Table S13. The Effect of Somatic Genetic Testing Availability on the Diagnostic Performance of PASS

					PASS score with somatic component				PASS score omitting somatic component			
**Cohort**	**Total Patients**	**Patients with available somatic data[1]**	**AA**	**IBMFS**	**ROC (95% CI)**	**PPV for AA at PASS ≥30**	**PPV for IBMFS at PASS <0**	**PPV for AA at PASS 0–20**	**ROC (95% CI)**	**PPV for AA at PASS ≥30**	**PPV for IBMFS at PASS <0**	**PPV for AA at PASS 0–20**
**Training cohort (Penn)**	**212**	189 (89.2%)	155	34	0.988 (0.977–0.998)	129/129 (100%)	31/38 (81.8%)	19/22 (86.4%)	0.986 (0.973-0.998)	119/119 (100%)	32/42 (76.2%)	26/28 (92.9%)
**All validation cohorts**	**716**	652 (91.1%)	542	110	0.979 (0.969-0.988)	434/434 (100%)	97/119 (81.5%)	86/99 (86.9%)	0.975 (0.963-0.985)	395/395 (100%)	99/129 (76.7%)	117/128 (91.4%)
**UTSW**	**78**	71 (91%)	66	5	0.955 (0.888–1.000)	50/50 (100%)	3/5 (60%)	14/16 (87.5%)	0.927 (0.823–1.000)	36/36 (100%)	3/8 (37.5%)	25/27 (92.6%)
**MDA**	**121**	114 (94.2%)	65	49	0.978 (0.954-1)	53/53 (100%)	44/47 (93.6%)	9/14 (64.3%)	0.976 (0.951–0.999)	49/49 (100%)	45/48 (93.8%)	13/17 (76.5%)
**NIH/USP**	**247**	236 (95.5%)	195	41	0.971 (0.951-0.991)	145/145 (100%)	37/48 (77.1%)	39/43 (90.7%)	0.969 (0.947–0.990)	134/134 (100%)	38/52 (73.1%)	47/50 (94.0%)
**RIME**	**270**	231 (85.6%)	216	15	0.984 (0.970-0.998)	186/186 (100%)	13/19 (68.4%)	24/26 (92.3%)	0.981 (0.964-0.997)	176/176 (100%)	13/21 (61.9%)	32/34 (94.1%)
**All patients combined**	**928**	841 (90.6%)	697	144	0.981 (0.973-0.988)	563/563 (100%)	128/157 (81.5%)	105/121 (86.7%)	0.977 (0.968-0.986)	514/514 (100%)	131/171 (76.6%)	143/156 (91.6%)
**Performance metrics[2]**					**Brier score= 0.039**	**HL χ²(df)= 3.61 (7); p=0.607**			**Brier 0.041**	**HL χ²(df)= 2.02(7); p=0.846**		

[1]Analysis performed on a subset of patients who had one or more of the available somatic tests performed (PNH flow cytometry, next-generation sequencing, cytogenetics, or cytogenomic array). [2]Calibratoni performance metrics calculated for all patients combined.

Supplemental Table S14. The Effect of the Availability of Both Telomere Lengths and Somatic Genetic Testing on the Diagnostic Performance of PASS

					PASS score with TL and somatic components				PASS score omitting TL and somatic components			
**Cohort**	**Total Patients**	**Patients with available TL and somatic data** [1]	**AA**	**IBMFS**	**ROC (95% CI)**	**PPV for AA at PASS ≥30**	**PPV for IBMFS at PASS <0**	**PPV for AA at PASS 0–20**	**ROC (95% CI)**	**PPV for AA at PASS ≥30**	**PPV for IBMFS at PASS <0**	**PPV for AA at PASS 0–20**
**Training cohort**	**212**	100 (47.2%)	77	23	0.982 (0.963–1.000)	55/55 (100%)	21/26 (80.8%)	17/19 (89.5%)	0.978 (0.956–1.000)	49/49 (100%)	21/28 (75.0%)	21/23 (91.3%)
**All validation cohorts**	**716**	319 (44.6%)	240	79	0.969 (0.952-0.986)	174/174 (100%)	71/86 (82.6%)	51/59 (86.4%)	0.936 (0.908-0.964)	172/174 (98.9%)	59/69 (85.5%)	58/76 (76.3%)
**UTSW**	**78**	10 (12.8%)	5	5	1 (1-1)	5/5 (100%)	3/3 (100%)	0/2 (0%)	0.980 (0.924 -1.0)	2/2 (100%)	3/3 (100%)	3/5 (60%)
**MD Anderson**	**121**	36 (29.8%)	13	23	0.931 (0.836-1)	7/7 (100%)	22/24 (91.7%)	4/5 (80%)	0.871 (0.731-1)	6/7 (85.7%)	18/20 (90.0%)	5/9 (55.6%)
**NIH/USP**	**247**	236 (95.5%)	196	41	0.971 (0.951-0.991)	145/145 (100%)	37/48 (77.1%)	39/43 (90.7%)	0.931 (0.894-0.968)	143/144 (99.3%)	30/37 (81.1%)	46/56 (82.1%)
**RIME**	**270**	37 (13.7%)	27	10	0.972 (0.930-1.0)	17/17 (100%)	9/11 (81.8%)	8/9 (88.9%)	0.961 (0.909-1.0)	21/21 (100%)	8/9 (88.9%)	5/7 (71.4%)
**All patients total**	**928**	419 (45.1%)	317	102	0.973 (0.959-0.986)	229/229 (100%)	92/112 (82.1%)	68/78 (87.2%)	0.943 (0.920-0.966)	221/223 (99.1%)	80/97 (82.5%)	79/99 (79.8%)
**Performance metrics**[2]					**Brier score = 0.051**	**HL χ^2(df)= 3.11 (8) (p=0.794)**			**Brier score = 0.074**	**HL χ^2(df)= 4.07 (6) (p=0.396)**		

[1]Analysis performed on a subset of patients who had available TL testing and at least one of the following somatic testing performed (PNH flow cytometry, next-generation sequencing, cytogenetics, or cytogenomic array). [2]Calibratoni performance metrics calculated for all patients combined.

Supplemental Table S15: 8 NIH-USP IBMFS Patients Diagnosed as AA or Unable to be Classified by NIH-ML Model

Study ID	True Diagnosis	Diagnosis detail	NIH Model Prediction	PASS Score	IBMFS red flag	Acuity	Severity	PNH	Telomere
NIH216	IBMFS	TBD (TERT)	No prediction by model	-40	No red flags	Chronic	NSAA	none	Under1
330-1	IBMFS	SDS (SBDS)	No prediction by model	-20	Red flag	Acute	NSAA	none	Normal
353-1	IBMFS	TBD (TERT)	No prediction by model	-40	No red flags	Chronic	NSAA	none	Under1
NIH318	IBMFS	TBD (TERC)	No prediction by model	-10	No red flags	Acute	NSAA	none	Under1
NIH012	IBMFS	FA	AA	10	No red flags	Acute	NSAA	none	Normal
USP026	IBMFS	TBD (TERT)	AA	-20	No red flags	Chronic	NSAA	none	Under10
USP022	IBMFS	TBD (TERT)	AA	10	No red flags	Acute	NSAA	Yes (6% clone)	Under1
USP030	IBMFS	TBD (TINF2)	AA	-10	No red flags	Acute	NSAA	none	Under1

Supplemental Table S16: 22 IBMFS Patients Predicted to Have AA by the RIME Model

StudyID	True Diagnosis	Diagnosis detail	RIME model prediction	PASS	Morphology	Acuity	Severity	PNH	Telomere
MDA070	IBMFS	GATA2 deficiency syndrome	AA	10	No red flags	Acute	NSAA	PNH Positive (≥0.1%)	NA
MDA074	IBMFS	TBD	AA	20	No red flags	Acute	SAA/VSAA	PNH Negative (<0.1%)	Short (<1)
MDA080	IBMFS	SDS	AA	-20	No red flags	Acute	NSAA	PNH Negative (<0.1%)	NA
MDA082	IBMFS	DBA	AA	10	No red flags	Acute	SAA/VSAA	PNH Negative (<0.1%)	NA
MDA090	IBMFS	GATA2 deficiency syndrome	AA	-20	No red flags	Acute	NSAA	not done	NA
MDA097	IBMFS	TBD	AA	-10	No red flags	Acute	NSAA	PNH Negative (<0.1%)	Short (<1)
MDA105	IBMFS	TBD	AA	-40	No red flags	Acute	NSAA	not done	Short (<1)
MDA111	IBMFS	TBD	AA	-40	No red flags	Acute	NSAA	not done	Short (<1)
MDA112	IBMFS	GATA2 deficiency syndrome	AA	-20	No red flags	Acute	NSAA	PNH Positive (≥0.1%)	NA
MDA113	IBMFS	GATA2 deficiency syndrome	AA	-20	No red flags	Acute	NSAA	not done	NA
MDA114	IBMFS	TBD	AA	-40	No red flags	Acute	NSAA	PNH Negative (<0.1%)	Short (<1)
MDA120	IBMFS	Germline ANKRD26	AA	0	No red flags	Acute	NSAA	not done	NA

NIH004	IBMFS	TBD (TERC)	AA	-40	No red flags	Acute	NSAA	PNH Negative (<0.1%)	Short (<1)
NIH005	IBMFS	TBD (TERT)	AA	-40	No red flags	Acute	NSAA	PNH Negative (<0.1%)	Short (<1)
NIH006	IBMFS	TBD (TERT)	AA	-10	No red flags	Acute	NSAA	PNH Negative (<0.1%)	Short (<1)
NIH012	IBMFS	FA	AA	10	No red flags	Acute	NSAA	PNH Negative (<0.1%)	Normal
NIH305	IBMFS	TBD (TERT, PARN)	AA	-40	No red flags	Acute	NSAA	PNH Negative (<0.1%)	Short (<1)
USP022	IBMFS	TBD (TERT)	AA	10	No red flags	Acute	NSAA	PNH Positive (≥0.1%)	Short (<1)
PENN045	IBMFS	TBD	AA	-40	No red flags	Acute	NSAA	not done	Short (<1)
PENN058	IBMFS	TBD	AA	-20	No red flags	Acute	NSAA	not done	Normal
PENN197	IBMFS	WAS	AA	10	No red flags	Acute	NSAA	PNH Negative (<0.1%)	Normal
PENN204	IBMFS	FA	AA	-20	No red flags	Acute	NSAA	not done	NA

Supplemental Figures

Supplemental Figure S1. Selection of NIH/USP cohort patient inclusion/exclusion

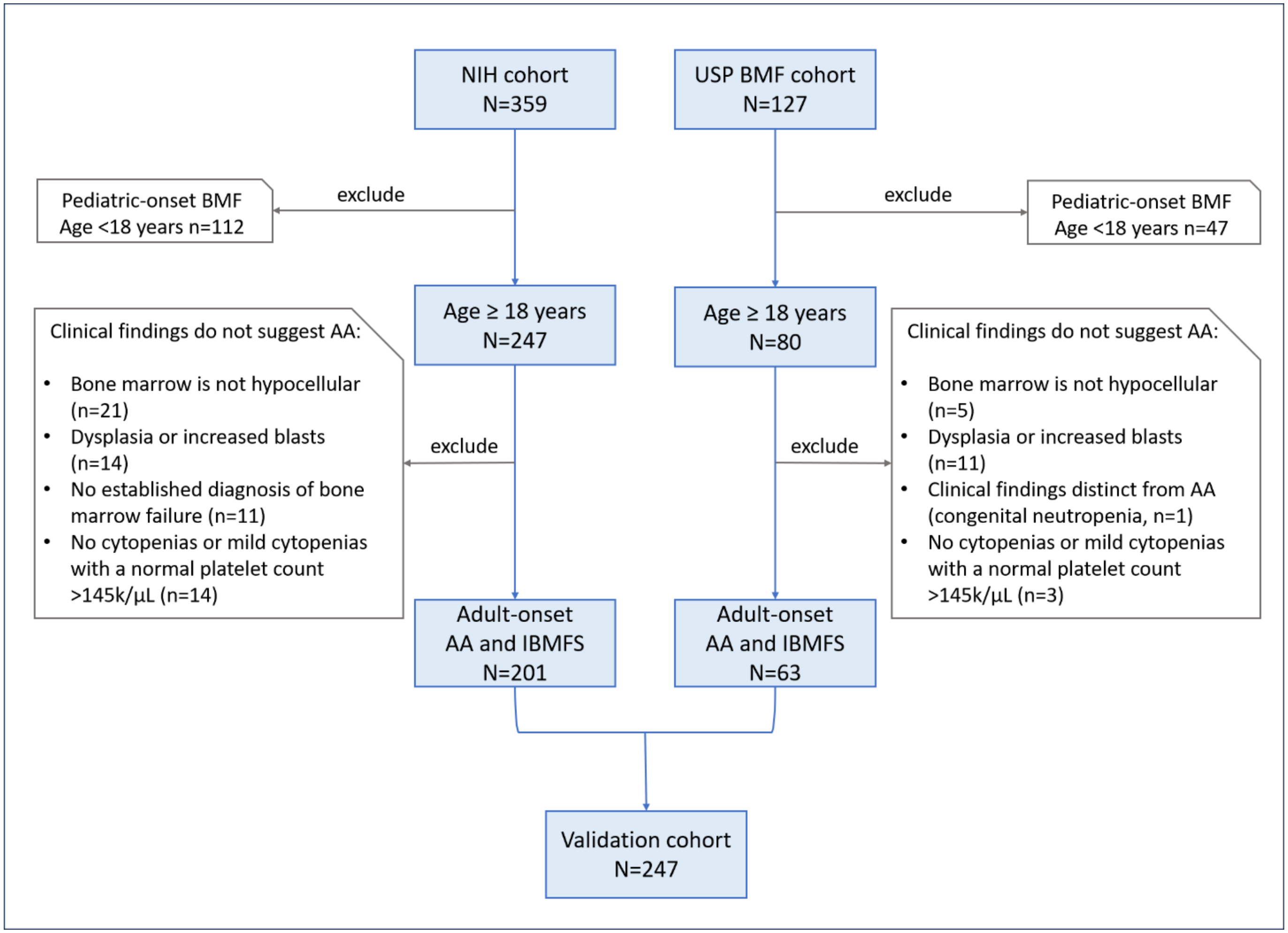
NIH cohort
N=359
USP BMF cohort
N=127
exclude
Pediatric-onset BMF
Age <18 years n=112
exclude
Pediatric-onset BMF
Age <18 years n=47
Age ≥ 18 years
N=247
Age ≥ 18 years
N=80
Clinical findings do not suggest AA:
• Bone marrow is not hypocellular (n=21)
• Dysplasia or increased blasts (n=14)
• No established diagnosis of bone marrow failure (n=11)
• No cytopenias or mild cytopenias with a normal platelet count >145k/μL (n=14)
exclude
exclude
Clinical findings do not suggest AA:
• Bone marrow is not hypocellular (n=5)
• Dysplasia or increased blasts (n=11)
• Clinical findings distinct from AA (congenital neutropenia, n=1)
• No cytopenias or mild cytopenias with a normal platelet count >145k/μL (n=3)
Adult-onset
AA and IBMFS
N=201
Adult-onset
AA and IBMFS
N=63
Validation cohort
N=247

Supplemental Figure S2 The PASS model performance in the training cohort, calculated using the most sensitive PNH threshold and using all AA-associated somatic findings detected over the course of the disease.

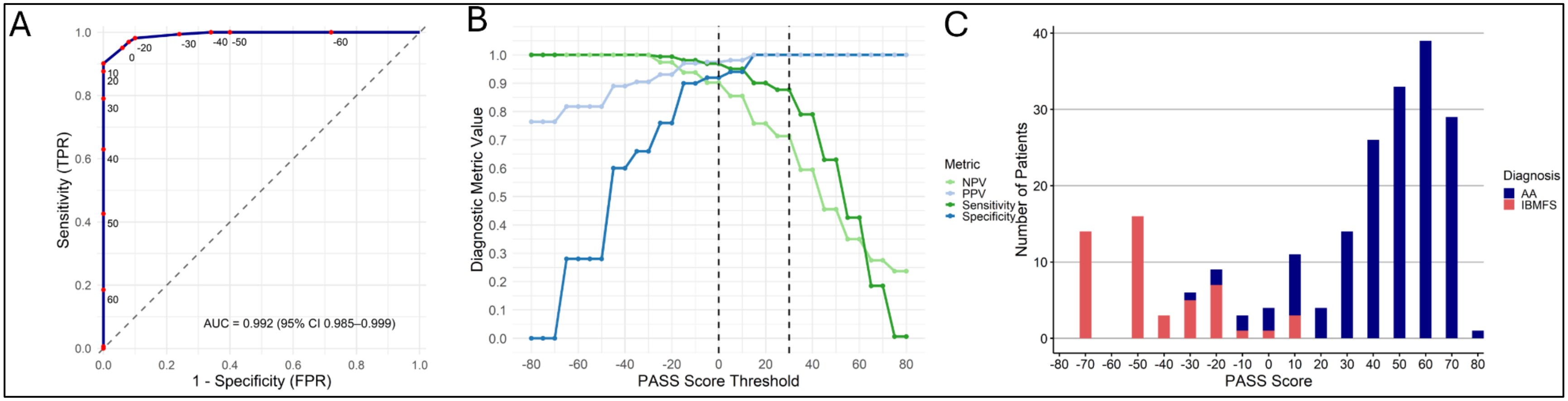

A) The ROC curve, showing the sensitivity (true positive rate, TPR) on the Y-axis against the false positive rate (FPR, 1 − specificity) on the X-axis for scores generated with the PASS model in the training cohort. The red dots correspond to labeled scores in the training cohort. Ideal discrimination allows perfect sensitivity without false positives (upper left quadrant). The dashed diagonal line indicates no discrimination between true and false positives (random). The PASS score shows an excellent area under the curve (AUC), indicating near-perfect diagnostic performance. **B)** A plot demonstrating the PASS score performance characteristics (plotted on the Y-axis) across a range of score thresholds (on the X-axis). Plotted are the PPV (light blue), specificity (dark blue), NPV (light green), and sensitivity (dark green). The dashed vertical lines are shown at a score of 30 (demarcating scores with 100% PPV and specificity for AA), and at a score of 0 (demarcating a threshold below which the probability of an AA diagnosis starts to fall). **C)** Distribution of AA and IBMFS diagnoses across the range of PASS scores (on X-axis), demonstrating excellent separation between the two diagnostic categories, with high specificity for AA for positive PASS scores of 30 and higher.

Supplemental Figure S3 The effect of telomere length availability on PASS performance.

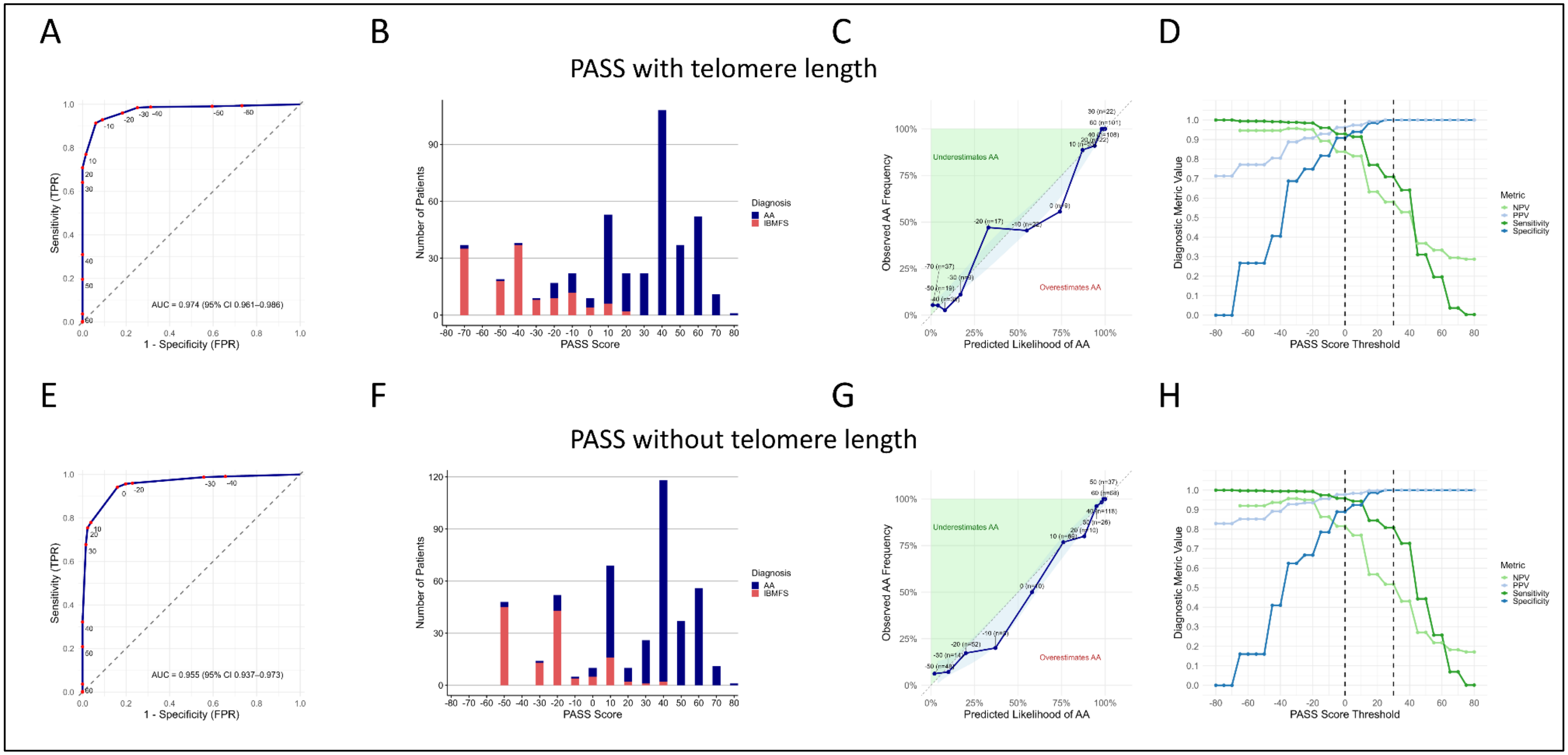

The effect of telomere length availability on PASS performance. Shown are the results of sensitivity analysis of patients with available telomere length measurements. PASS performance with telomere length included is shown in panels A–D, and PASS performance without telomere length is shown in panels E–H. Plotted are comparisons of receiver operating characteristic (ROC) curves (A, E), PASS score distributions by diagnosis (B, F), calibration of predicted probability of acquired aplastic anemia (AA) versus observed frequency (C, G), and PASS score performance metrics across score thresholds (D, H). ROC curves display sensitivity versus 1 − specificity, with dashed diagonal lines indicating no discrimination. Score distribution panels show the frequency of AA and inherited bone marrow failure syndromes (IBMFS) diagnoses across the range of PASS scores. Calibration plots compare predicted AA probabilities derived from logistic regression models using PASS score to observed AA frequencies, with points representing bins of rounded predicted probabilities and a dashed diagonal indicating perfect calibration; shaded regions represent LOESS-smoothed fits with 95% confidence intervals. Performance metric plots display positive predictive value (PPV), specificity, negative predictive value (NPV), and sensitivity across PASS score thresholds, with dashed vertical lines indicating thresholds at scores of 30 and 0.

Supplemental Figure S4 The effect of somatic genetic test availability on PASS performance

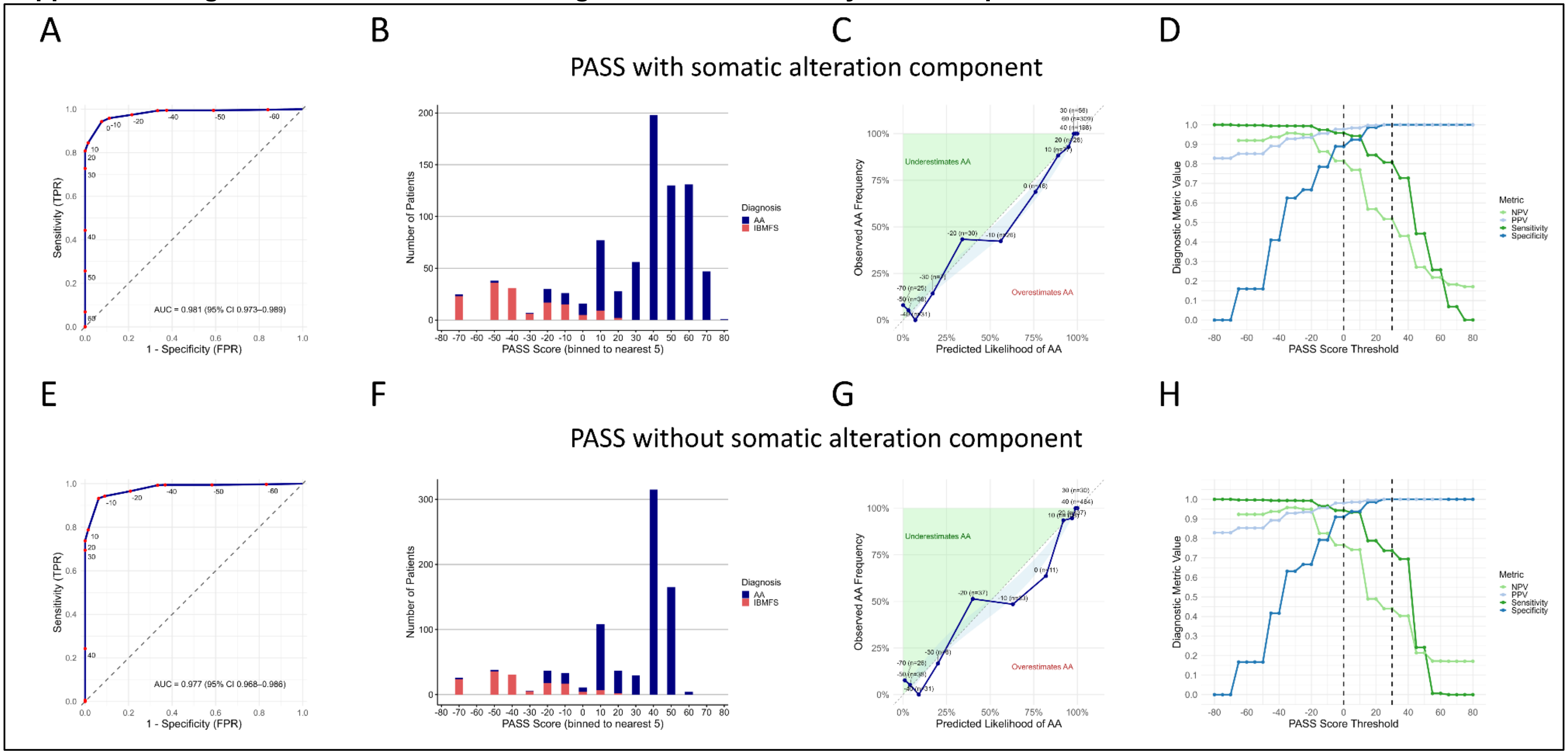

The effect of somatic score component on PASS performance. Shown are the results of sensitivity analyses restricted to patients with available results for somatic score component. PASS performance with the somatic component included is shown in panels A–D, and PASS performance with the somatic component omitted is shown in panels E–H. Plotted are comparisons of receiver operating characteristic (ROC) curves (A, E), PASS score distributions by diagnosis (B, F), calibration of predicted probability of acquired aplastic anemia (AA) versus observed frequency (C, G), and PASS score performance metrics across score thresholds (D, H). ROC curves display sensitivity versus 1 − specificity, with dashed diagonal lines indicating no discrimination. Score distribution panels show the frequency of AA and inherited bone marrow failure syndromes (IBMFS) diagnoses across the range of PASS scores. Calibration plots compare predicted AA probabilities derived from logistic regression models using the PASS score to observed AA frequencies, with points representing bins of rounded predicted probabilities and a dashed diagonal indicating perfect calibration; shaded regions represent LOESS-smoothed fits with 95% confidence intervals. Performance metric plots display positive predictive value (PPV), specificity, negative predictive value (NPV), and sensitivity across PASS score thresholds, with dashed vertical lines indicating thresholds at scores of 30 and 0.

Supplemental Figure S5 The effect of availability of both telomere length and somatic test components on PASS performance

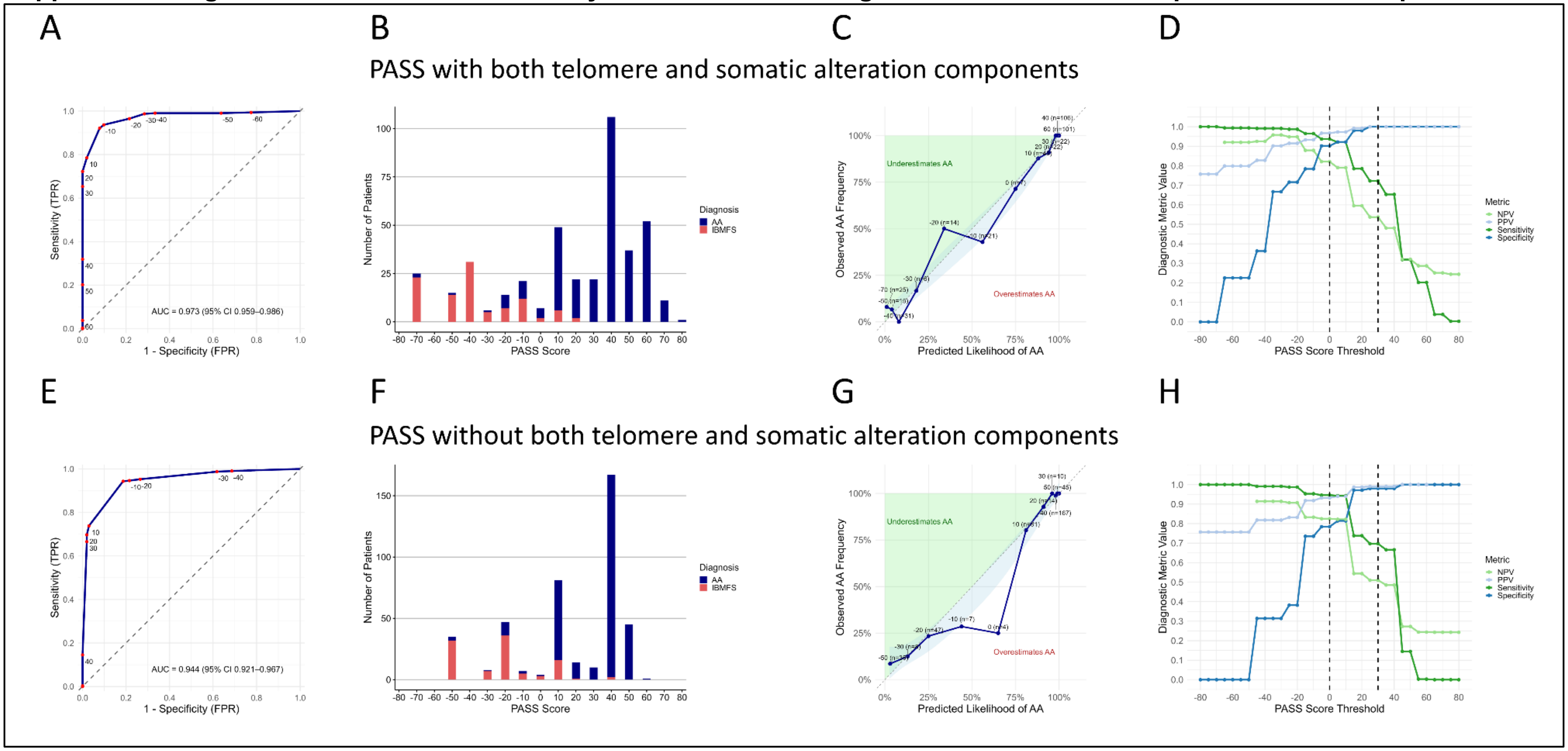

The effect of availability of both telomere length and somatic test components on PASS performance. **The effect of somatic score component on PASS performance.** Shown are the results of sensitivity analyses restricted to patients with available results for both telomere lengths and the somatic score component. PASS performance with TL and somatic component included is shown in panels A–D, and PASS performance with both TL and somatic component omitted is shown in panels E–H. Plotted are comparisons of receiver operating characteristic (ROC) curves (A, E), PASS score distributions by diagnosis (B, F), calibration of predicted probability of acquired aplastic anemia (AA) versus observed frequency (C, G), and PASS score performance metrics across score thresholds (D, H). ROC curves display sensitivity versus 1 − specificity, with dashed diagonal lines indicating no discrimination. Score distribution panels show the frequency of AA and inherited bone marrow failure syndromes (IBMFS) diagnoses across the range of PASS scores. Calibration plots compare predicted AA probabilities derived from logistic regression models using the PASS score to observed AA frequencies, with points representing bins of rounded predicted probabilities and a dashed diagonal indicating perfect calibration; shaded regions represent LOESS-smoothed fits with 95% confidence intervals. Performance metric plots display positive predictive value (PPV), specificity, negative predictive value (NPV), and sensitivity across PASS score thresholds, with dashed vertical lines indicating thresholds at scores of 30 and 0.

Supplemental References

1. Gutierrez-Rodrigues F, Munger E, Ma X, et al. Differential diagnosis of bone marrow failure syndromes guided by machine learning. *Blood*. 2023;141(17):2100-2113.
2. Alter BP. Diagnosis, genetics, and management of inherited bone marrow failure syndromes. *Hematology Am Soc Hematol Educ Program*. 2007:29-39.
3. West AH, Churpek JE. Old and new tools in the clinical diagnosis of inherited bone marrow failure syndromes. *Hematology Am Soc Hematol Educ Program*. 2017;2017(1):79-87.
4. Incidence of aplastic anemia: the relevance of diagnostic criteria. By the International Agranulocytosis and Aplastic Anemia Study. *Blood*. 1987;70(6):1718-1721.
5. Kulasekararaj A, Cavenagh J, Dokal I, et al. Guidelines for the diagnosis and management of adult aplastic anaemia: A British Society for Haematology Guideline. *Br J Haematol*. 2024;204(3):784-804.
6. Gutierrez-Rodrigues F, Patel BA, Groarke EM. When to consider inherited marrow failure syndromes in adults. *Hematology Am Soc Hematol Educ Program*. 2023;2023(1):548-555.
7. DeZern AE, Churpek JE. Approach to the diagnosis of aplastic anemia. *Blood Adv*. 2021;5(12):2660-2671.
8. Townsley DM, Scheinberg P, Winkler T, et al. Eltrombopag Added to Standard Immunosuppression for Aplastic Anemia. *N Engl J Med*. 2017;376(16):1540-1550.
9. Dasari S, Tse W, Wang J. Real-world evidence of incidence and outcomes of aplastic anaemia following administration of immune checkpoint inhibitors. *Br J Haematol*. 2023;202(6):1205-1208.
10. de Masson A, Bouaziz JD, de Latour RP, et al. Severe aplastic anemia associated with eosinophilic fasciitis: report of 4 cases and review of the literature. *Medicine (Baltimore)*. 2013;92(2):69-81.
11. Gendron N, de Fontbrune FS, Guyard A, et al. Aplastic anemia related to thymoma: a survey on behalf of the French reference center of aplastic anemia and a review of the literature. *Haematologica*. 2020;105(7):e333-e336.
12. Oriol A, Ribera JM, Hernandez A, Soriano V, Milla F, Feliu E. Aplastic anemia after non-A, non-B, and non-C hepatitis. *Haematologica*. 1994;79(2):168-169.
13. Rovo A, Kulasekararaj A, Medinger M, et al. Association of aplastic anaemia and lymphoma: a report from the severe aplastic anaemia working party of the European Society of Blood and Bone Marrow Transplantation. *Br J Haematol*. 2019;184(2):294-298.
14. Solhaug TS, Tjonnfjord GE, Bjorgo K, Kildahl-Andersen O. A family with cytotoxic T-lymphocyte-associated protein 4 haploinsufficiency presenting with aplastic anaemia. *BMJ Case Rep*. 2022;15(2).
15. Linaburg T, Davis AR, Frey NV, et al. Hodgkin lymphoma patients have an increased incidence of idiopathic acquired aplastic anemia. *PLoS One*. 2019;14(4):e0215021.
16. Camitta BM. Pathogenesis and treatment of aplastic anemia. *Rinsho Ketsueki*. 1984;25(4):459-469.
17. Rovo A, Tichelli A, Dufour C, Saa-Wp E. Diagnosis of acquired aplastic anemia. *Bone Marrow Transplant*. 2013;48(2):162-167.
18. Tibshirani R. Regression Shrinkage and Selection Via the Lasso. *Journal of the Royal Statistical Society: Series B (Methodological)*. 2018;58(1):267-288.
19. Hanley JA, McNeil BJ. The meaning and use of the area under a receiver operating characteristic (ROC) curve. *Radiology*. 1982;143(1):29-36.
20. Brier GW. Verification of forecasts expressed in terms of probability. *Monthly Weather Review*. 1950;78(1):1-3.
21. Kaphan E, Walter-Petrich A, Larcher L, et al. Recursive Partitioning to Differentiate Acquired From Inherited Bone Marrow Failure Syndromes. *Am J Hematol*. 2025;100(11):1983-1992.
22. StataCorp. Stata Statistical Software College Station, TX:: StataCorp LLC; 2025.

23. Parker CJ. Management of paroxysmal nocturnal hemoglobinuria in the era of complement inhibitory therapy. *Hematology Am Soc Hematol Educ Program*. 2011;2011:21-29.
24. Illingworth AJ, Marinov I, Sutherland DR. Immunophenotyping of Paroxysmal Nocturnal Hemoglobinuria (PNH). *Methods Mol Biol*. 2019;2032:323-354.
25. Babushok DV, Xie HM, Roth JJ, et al. Single nucleotide polymorphism array analysis of bone marrow failure patients reveals characteristic patterns of genetic changes. *Br J Haematol*. 2014;164(1):73-82.
26. Afable MG, 2nd, Tiu RV, Maciejewski JP. Clonal evolution in aplastic anemia. *Hematology Am Soc Hematol Educ Program*. 2011;2011:90-95.
27. Sande CM, Chen S, Mitchell DV, et al. ATM-dependent DNA damage response constrains cell growth and drives clonal hematopoiesis in telomere biology disorders. *JCI*. 2025;In Press.
28. Aubert G, Baerlocher GM, Vulto I, Poon SS, Lansdorp PM. Collapse of telomere homeostasis in hematopoietic cells caused by heterozygous mutations in telomerase genes. *PLoS Genet*. 2012;8(5):e1002696.
29. Alder JK, Hanumanthu VS, Strong MA, et al. Diagnostic utility of telomere length testing in a hospital-based setting. *Proc Natl Acad Sci U S A*. 2018;115(10):E2358-E2365.
30. Ji X, Kattan MW. Tutorial: development of an online risk calculator platform. *Ann Transl Med*. 2018;6(3):46.

Supplemental Datasets

Supplemental Dataset S1. Training cohort individual-level clinical data

Individual-level clinical data from the Penn training cohort. Age at bone marrow failure (BMF) evaluation refers to the patient's age at the time of diagnostic assessment. Documentation of a normal complete blood count (CBC) prior to BMF diagnosis is noted where available. Years from the last normal CBC to BMF diagnosis and years from the first known abnormal CBC to diagnosis are listed. Cytopenia severity is defined according to the modified Camitta criteria. PNH clone size is reported at diagnosis and updated if any additional findings emerged later at any point in the disease course; values ≥0.5% are considered positive. The presence of any of the following—PNH ≥0.5%, 6pLOH, BCOR mutation, or del(13q)—at diagnosis or at any point in the disease course contributes to the somatic score. AA-associated conditions are defined in Supplemental Table S2, and IBMFS red flags are listed in Supplemental Table S1. Lymphocyte and granulocyte telomere lengths are reported, with values <1st percentile considered abnormal. Chromosome breakage testing and germline genetic testing are recorded where available. Immunosuppressive therapy (IST) status is indicated as Yes, No, or Unknown, and response at 6 months is categorized as complete response (CR), partial response (PR), no response (NR), or not evaluable. Bone marrow transplant (BMT) status, duration of follow-up, and status at last follow-up are included. Justification for diagnosis assignment is based on clinical, genetic, and treatment response data. Study-adjudicated diagnoses reflect final classification after expert review.

PennUPN	Diagnosis	Age at BMF evaluation	Is there a documented normal CBC?	Years from last normal CBC to BMF diagnosis	Years from first abnormal to BMF diagnosis	Cytopenia Severity	PNH clone at diagnosis	PNH clone at any point in disease course	Any of PNH >0.5%, 6pLOH, BCOR, del13q AT DIAGNOSIS	Any of PNH >0.5%, 6pLOH, BCOR or del 13q at ANY POINT	AA associated conditions	IBMF red flags	Lymphocyte telomere length	Granulocyte telomere length	Chromosome Breakage	Germline genetic testing	IST (Yes, No, unknown)	Response to IST at 6 months	BMT	Follow-up (Years)	Status at last follow-up	Justification for diagnosis assignment	Study Adjudicated Diagnosis
PENN001	AA	65.4	normal_exists	2.0	0.27	SAA/VSAA	0.00	0.00	.	Present	.	.	NA	NA	NA	Not done	Yes	Unknown	Yes	2.65	Alive	IBMFS genetics not tested AND no response/not evaluable resonse/no IST given BUT a clinical diagnosis of AA	Presumed AA
PENN002	IBMFS	31.7	normal_exists	5.0	1.52	NSAA	0.00	0.00	.	.	.	AVN	Under1	Under1	0	Done	Yes	Refractory	No	5.53	Alive	No genetic diagnosis of IBMFS, but functional and clinical data suggestive of IBMFS	Presumed IBMFS NOS
PENN003	AA	40.8	no_prior	no_prior	0.04	SAA/VSAA	0.00	0.00	.	.	.	.	NA	NA	0	Not done	Yes	Refractory	Yes	13.26	Alive	IBMFS genetics not tested, BUT responded to IST (PR or CR)	Confirmed AA
PENN004	AA	40.0	no_prior	no_prior	0.00	SAA/VSAA	0.00	0.00	.	.	.	.	NA	NA	NA	Done	Yes	CR	No	3.27	Alive	IBMFS genetic testing negative AND responded to IST	Confirmed AA
PENN005	AA	53.7	normal_exists	0.2	0.06	SAA/VSAA	0.00	0.00	.	6pLOH	.	.	Between1and10	Under1	0	Not done	Yes	PR	Yes	1.53	Dead	IBMFS genetics not tested, BUT responded to IST (PR or CR)	Confirmed AA
PENN006	AA	55.7	normal_exists	0.8	0.27	SAA/VSAA	0.00	0.00	.	.	.	.	Under1	Under1	0	Not done	Yes	Refractory	Yes	1.75	Dead	IBMFS genetics not tested AND no response/not evaluable resonse/no IST given BUT a clinical diagnosis of AA	Presumed AA
PENN007	AA	56.4	no_prior	no_prior	0.14	SAA/VSAA	0.00	0.00	.	.	.	.	NA	NA	NA	Not done	Yes	CR	No	12.00	Alive	IBMFS genetics not tested, BUT responded to IST (PR or CR)	Confirmed AA
PENN008	AA	45.9	normal_exists	0.3	0.16	SAA/VSAA	0.00	0.00	.	.	Hodgkins	.	NA	NA	NA	Not done	Yes	Unknown	No	0.39	Dead	IBMFS genetics not tested AND no response/not evaluable resonse/no IST given BUT a clinical diagnosis of AA	Presumed AA
PENN009	AA	75.0	no_prior	no_prior	0.01	SAA/VSAA	0.00	0.00	.	.	.	.	NA	NA	NA	Not done	Yes	CR	No	5.07	Dead	IBMFS genetics not tested, BUT responded to IST (PR or CR)	Confirmed AA
PENN010	AA	73.8	normal_exists	1.6	0.92	SAA/VSAA	small <0.5%	small <0.5%	.	.	.	.	NA	NA	NA	Not done	Yes	PR	No	3.56	Alive	IBMFS genetics not tested, BUT responded to IST (PR or CR)	Confirmed AA
PENN011	IBMFS	36.5	abnormalities_since_childhood	lifelong	1.22	NSAA	NA	NA	.	.	.	congenital abnormality	NA	NA	NA	Done	No	Not Applicable- not treated with IST	No	7.16	Alive	Confirmed IBMFS genetic and/or functional test (breakage/telomere) diagnostic of IBMFS	Confirmed IBMFS
PENN012	IBMFS	18.8	normal_exists	4.3	2.02	NSAA	0.00	0.00	.	.	.	cirrhosis	Between1and10	Normal	NA	Done	No	Not Applicable- not treated with IST	No	4.79	Alive	No genetic diagnosis of IBMFS, but functional and clinical data suggestive of IBMFS	Presumed IBMFS NOS
PENN013	AA	57.6	normal_exists	5.0	0.00	SAA/VSAA	small <0.5%	small <0.5%	PNH	.	.	.	NA	NA	NA	Not done	Yes	PR	No	3.43	Alive	IBMFS genetics not tested, BUT responded to IST (PR or CR)	Confirmed AA
PENN014	AA	77.5	no_prior	no_prior	0.01	NSAA	small <0.5%	small <0.5%	.	.	.	.	NA	NA	NA	Not done	Yes	PR	No	3.64	Alive	IBMFS genetics not tested, BUT responded to IST (PR or CR)	Confirmed AA
PENN015	AA	42.1	no_prior	no_prior	0.08	NSAA	≥1%	≥1%	PNH	PNH	.	.	Under1	NA	0	Not done	Yes	PR	No	6.09	Alive	IBMFS genetics not tested, BUT responded to IST (PR or CR)	Confirmed AA
PENN016	AA	85.4	normal_exists	1.3	0.44	SAA/VSAA	0.00	0.00	.	.	.	.	NA	NA	NA	Not done	Yes	PR	No	0.45	Dead	IBMFS genetics not tested, BUT responded to IST (PR or CR)	Confirmed AA
PENN017	AA	68.4	normal_exists	3.6	0.04	SAA/VSAA	≥1%	≥1%	PNH, del13q	PNH	.	.	NA	NA	NA	Not done	Yes	PR	No	5.01	Alive	IBMFS genetics not tested, BUT responded to IST (PR or CR)	Confirmed AA
PENN018	AA	74.2	no_prior	no_prior	0.02	SAA/VSAA	NA	NA	.	.	.	.	NA	NA	NA	Not done	Yes	Not Applicable- died or transplanted before 6 mo	No	0.53	Dead	IBMFS genetics not tested AND no response/not evaluable resonse/no IST given BUT a clinical diagnosis of AA	Presumed AA
PENN019	AA	19.4	normal_exists	1.9	0.00	SAA/VSAA	0.00	0.00	.	.	.	.	Between1and10	Between1and10	0	Not done	Yes	PR	Yes	3.10	Alive	IBMFS genetics not tested, BUT responded to IST (PR or CR)	Confirmed AA
PENN020	AA	85.4	normal_exists	2.8	0.24	SAA/VSAA	0.00	0.00	.	.	.	.	NA	NA	NA	Not done	Yes	Refractory	No	1.32	Dead	IBMFS genetics not tested AND no response/not evaluable resonse/no IST given BUT a clinical diagnosis of AA	Presumed AA
PENN021	AA	50.1	normal_exists	2.6	0.02	SAA/VSAA	small <0.5%	small <0.5%	.	.	.	.	Between1and10	Under1	0	Not done	Yes	PR	No	2.85	Alive	IBMFS genetics not tested, BUT responded to IST (PR or CR)	Confirmed AA
PENN022	AA	70.1	no_prior	no_prior	0.00	SAA/VSAA	0.00	0.00	.	.	.	.	NA	NA	NA	Not done	Yes	PR	No	2.97	Alive	IBMFS genetics not tested, BUT responded to IST (PR or CR)	Confirmed AA
PENN023	AA	24.6	no_prior	no_prior	0.14	SAA/VSAA	≥10%	≥10%	PNH	PNH	.	.	NA	NA	NA	Not done	Yes	PR	Yes	4.47	Alive	IBMFS genetics not tested, BUT responded to IST (PR or CR)	Confirmed AA
PENN024	AA	42.7	normal_exists	4.5	0.01	SAA/VSAA	0.00	0.00	.	.	.	.	Between1and10	Under1	0	Done	Yes	Not Applicable- died or transplanted before 6 mo	Yes	1.71	Dead	IBMFS genetic testing negative	Confirmed AA
PENN025	IBMFS	53.7	no_prior	no_prior	1.33	SAA/VSAA	0.00	0.00	.	.	.	SCC larynx, chemo sensitivity	NA	NA	1	Not done	No	Not Applicable- not treated with IST	No	0.88	Dead	Confirmed IBMFS genetic and/or functional test (breakage/telomere) diagnostic of IBMFS	Confirmed IBMFS

PENN026	AA	30.8	no_prior	no_prior	1.67	SAA/VSAA	0.00	0.00	.	.	.	.	Between1and10	Under1	0	Done	Yes	Refractory	Yes	3.22	Dead	IBMFS genetic testing negative	Confirmed AA
PENN027	AA	65.6	no_prior	no_prior	0.03	NSAA	≥1%	≥1%	PNH	PNH	.	.	NA	NA	NA	Not done	Yes	CR	No	6.12	Alive	IBMFS genetics not tested, BUT responded to IST (PR or CR)	Confirmed AA
PENN028	AA	68.6	no_prior	no_prior	0.05	SAA/VSAA	0.00	0.00	.	.	.	.	NA	NA	NA	Not done	Yes	CR	No	2.62	Alive	IBMFS genetics not tested, BUT responded to IST (PR or CR)	Confirmed AA
PENN029	AA	30.0	no_prior	no_prior	0.05	NSAA	≥10%	≥10%	PNH	PNH	.	.	NA	NA	NA	Not done	Yes	CR	No	0.61	Alive	IBMFS genetics not tested, BUT responded to IST (PR or CR)	Confirmed AA
PENN030	IBMFS	29.6	abnormalities_since_childhood	lifelong	29.29	NSAA	NA	NA	.	.	.	short stature	Under1	Under1	0	Done	No	Not Applicable-not treated with IST	No	0.81	Alive	Confirmed IBMFS genetic and/or functional test (breakage/telomere) diagnostic of IBMFS	Confirmed IBMFS
PENN031	AA	61.5	normal_exists	0.5	0.22	SAA/VSAA	≥10%	≥10%	PNH	6pLOH, PNH	.	.	Normal	Normal	NA	Not done	Yes	PR	Yes	12.33	Alive	IBMFS genetics not tested, BUT responded to IST (PR or CR)	Confirmed AA
PENN032	AA	54.7	normal_exists	0.8	0.28	SAA/VSAA	0.00	0.00	.	.	.	.	Normal	Normal	NA	Not done	Yes	PR	Yes	2.24	Alive	IBMFS genetics not tested, BUT responded to IST (PR or CR)	Confirmed AA
PENN033	IBMFS	68.3	normal_exists	4.6	3.59	NSAA	0.00	0.00	.	.	.	ILD, cirrhosis	Between1and10	Between1and10	0	Done	No	Not Applicable-not treated with IST	No	0.28	Dead	Confirmed IBMFS genetic and/or functional test (breakage/telomere) diagnostic of IBMFS	Confirmed IBMFS
PENN034	AA	63.0	normal_exists	1.8	0.98	NSAA	0.00	0.00	6pLOH	6pLOH	.	.	NA	NA	NA	Not done	No	Not Applicable-not treated with IST	No	1.15	Alive	IBMFS genetics not tested AND no response/not evaluable resonse/no IST given BUT a clinical diagnosis of AA	Presumed AA
PENN035	AA	35.4	no_prior	2.0	0.15	SAA/VSAA	0.00	0.00	.	.	.	.	Normal	Normal	0	Not done	Yes	CR	No	9.41	Alive	IBMFS genetics not tested, BUT responded to IST (PR or CR)	Confirmed AA
PENN036	AA	71.0	normal_exists	2.1	0.48	SAA/VSAA	0.00	0.00	.	.	.	.	NA	NA	NA	Not done	Yes	PR	No	2.23	Dead	IBMFS genetics not tested, BUT responded to IST (PR or CR)	Confirmed AA
PENN037	IBMFS	30.8	no_prior	no_prior	1.30	NSAA	0.00	0.00	.	.	.	squamous cell, dysmorphology	Normal	Between1and10	1	Done	No	Not Applicable-not treated with IST	Yes	12.06	Alive	Confirmed IBMFS genetic and/or functional test (breakage/telomere) diagnostic of IBMFS	Confirmed IBMFS
PENN038	IBMFS	32.8	no_prior	no_prior	0.50	SAA/VSAA	NA	NA	.	.	.	vulvar cancer, chemo sensitivity, thumb	NA	NA	1	Done	No	Not Applicable-not treated with IST	No	4.85	Alive	Confirmed IBMFS genetic and/or functional test (breakage/telomere) diagnostic of IBMFS	Confirmed IBMFS
PENN039	AA	41.0	normal_exists	0.5	0.52	NSAA	≥10%	≥10%	PNH	PNH	.	.	NA	NA	0	Not done	Yes	CR	Yes	3.45	Dead	IBMFS genetics not tested, BUT responded to IST (PR or CR)	Confirmed AA
PENN040	AA	27.6	normal_exists	6.2	1.49	NSAA	0.5 to <1%	0.5 to <1%	.	PNH	.	.	Normal	Normal	NA	Done	Yes	PR	No	1.86	Alive	IBMFS genetic testing negative AND responded to IST	Confirmed AA
PENN041	AA	67.5	no_prior	no_prior	1.30	SAA/VSAA	≥1%	≥1%	PNH	PNH	.	.	NA	NA	NA	Not done	Yes	Refractory	Yes	2.47	Alive	IBMFS genetics not tested, BUT responded to IST (PR or CR)	Presumed AA
PENN042	AA	34.1	no_prior	no_prior	7.07	SAA/VSAA	0.00	0.00	.	.	.	.	Normal	Normal	0	Not done	Yes	PR	Yes	4.83	Alive	IBMFS genetics not tested, BUT responded to IST (PR or CR)	Confirmed AA
PENN043	AA	46.6	normal_exists	0.6	0.01	SAA/VSAA	0.00	0.00	.	.	.	.	Between1and10	Under1	0	Not done	Yes	PR	No	7.45	Alive	IBMFS genetics not tested, BUT responded to IST (PR or CR)	Confirmed AA
PENN044	AA	56.4	no_prior	no_prior	0.69	SAA/VSAA	0.00	0.00	.	.	.	.	NA	NA	NA	Not done	Yes	PR	Yes	14.86	Dead	IBMFS genetics not tested, BUT responded to IST (PR or CR)	Confirmed AA
PENN045	IBMFS	38.9	normal_exists	8.2	0.75	NSAA	NA	NA	.	.	.	father IPF	Under1	Under1	NA	Done	No	Not Applicable-not treated with IST	Yes	1.49	Alive	Confirmed IBMFS genetic and/or functional test (breakage/telomere) diagnostic of IBMFS	Confirmed IBMFS
PENN046	AA	51.6	no_prior	no_prior	0.06	SAA/VSAA	small <0.5%	small <0.5%	.	.	.	.	Normal	NA	NA	Not done	Yes	CR	No	4.89	Alive	IBMFS genetics not tested, BUT responded to IST (PR or CR)	Confirmed AA
PENN047	AA	63.4	no_prior	no_prior	6.98	NSAA	0.00	0.00	.	.	.	sister leukemia	Between1and10	Under1	NA	Done	Yes	Refractory	No	3.46	Alive	IBMFS genetic testing negative	Confirmed AA
PENN048	AA	55.4	no_prior	no_prior	0.00	SAA/VSAA	small <0.5%	small <0.5%	.	.	.	.	Normal	Between1and10	0	Not done	Yes	PR	No	7.15	Alive	IBMFS genetics not tested, BUT responded to IST (PR or CR)	Confirmed AA
PENN049	AA	72.6	normal_exists	0.5	0.00	SAA/VSAA	0.00	0.00	6pLOH	6pLOH	.	.	Normal	Between1and10	0	Not done	Yes	Refractory	Yes	0.84	Alive	IBMFS genetics not tested, BUT responded to IST (PR or CR)	Presumed AA
PENN050	AA	61.0	normal_exists	3.4	0.12	SAA/VSAA	0.00	0.00	.	BCOR	.	.	Normal	Normal	0	Not done	Yes	PR	Yes	3.17	Alive	IBMFS genetics not tested, BUT responded to IST (PR or CR)	Confirmed AA
PENN051	IBMFS	44.8	abnormalities_since_childhood	lifelong	16.01	NSAA	NA	NA	.	.	.	ILD, AVN, mother with leukemia	Under1	Under1	NA	Done	No	Not Applicable-not treated with IST	Yes	0.08	Alive	Confirmed IBMFS genetic and/or functional test (breakage/telomere) diagnostic of IBMFS	Confirmed IBMFS
PENN052	AA	20.6	no_prior	no_prior	4.14	SAA/VSAA	≥1%	≥1%	PNH	PNH, BCOR	.	.	Normal	Between1and10	0	Not done	No	Not Applicable-not treated with IST	Yes	3.87	Alive	IBMFS genetics not tested AND no response/not evaluable resonse/no IST given BUT a clinical diagnosis of AA	Presumed AA
PENN053	AA	56.5	normal_exists	0.7	0.00	NSAA	0.00	0.00	.	.	.	.	Normal	Between1and10	NA	Done	Yes	PR	No	8.08	Alive	IBMFS genetic testing negative	Confirmed AA

PENN054	IBMFS	24.5	abnormalities_since_childhood	lifelong	23.72	NSAA	NA	NA	.	.	.	congenital abnormality	Normal	NA	0	Done	No	Not Applicable-not treated with IST	No	0.00	Alive	No genetic diagnosis of IBMFS, but functional and clinical data suggestive of IBMFS	Presumed IBMFS NOS
PENN055	AA	66.7	no_prior	no_prior	0.75	SAA/VSAA	0.00	0.00	.	.	.	.	Normal	NA	0	Not done	Yes	CR	No	6.10	Alive	IBMFS genetics not tested, BUT responded to IST (PR or CR)	Confirmed AA
PENN056	AA	26.7	normal_exists	2.8	0.04	SAA/VSAA	≥10%	≥10%	PNH	PNH	.	.	Between1and10	Under1	0	Not done	Yes	PR	No	4.74	Alive	IBMFS genetics not tested, BUT responded to IST (PR or CR)	Confirmed AA
PENN057	AA	23.6	no_prior	no_prior	0.01	SAA/VSAA	0.00	0.00	.	.	.	.	Normal	NA	0	Not done	Yes	CR	No	3.49	Alive	IBMFS genetics not tested, BUT responded to IST (PR or CR)	Confirmed AA
PENN058	IBMFS	38.2	no_prior	no_prior	0.00	NSAA	NA	NA	.	.	.	father with BMF	Between1and10	Between1and10	NA	Done	No	Not Applicable-not treated with IST	No	1.03	Alive	Confirmed IBMFS genetic and/or functional test (breakage/telomere) diagnostic of IBMFS	Confirmed IBMFS
PENN059	IBMFS	62.4	no_prior	no_prior	10.99	SAA/VSAA	0.00	0.00	.	.	.	chemotherapy sensitivity	Under1	Under1	0	Done	No	Not Applicable-not treated with IST	No	1.04	Dead	No genetic diagnosis of IBMFS, but functional and clinical data suggestive of IBMFS	Confirmed IBMFS
PENN060	AA	57.1	no_prior	no_prior	0.02	SAA/VSAA	0.00	0.00	.	.	.	.	Normal	Under1	0	Not done	Yes	CR	No	5.04	Alive	IBMFS genetics not tested, BUT responded to IST (PR or CR)	Confirmed AA
PENN061	AA	33.4	no_prior	no_prior	0.00	SAA/VSAA	≥10%	≥10%	PNH, BCOR	PNH, BCOR	.	.	NA	NA	0	Not done	Yes	PR	Yes	4.10	Alive	IBMFS genetics not tested, BUT responded to IST (PR or CR)	Confirmed AA
PENN062	AA	58.0	no_prior	no_prior	2.97	NSAA	small <0.5%	small <0.5%	.	.	.	.	NA	NA	NA	Not done	Yes	PR	No	6.72	Alive	IBMFS genetics not tested, BUT responded to IST (PR or CR)	Confirmed AA
PENN063	AA	30.1	normal_exists	7.5	0.98	SAA/VSAA	≥10%	≥10%	PNH	PNH	.	father with cytopenias	Normal	Normal	0	Not done	No	Not Applicable-not treated with IST	Yes	1.28	Alive	IBMFS genetics not tested, BUT responded to IST (PR or CR)	Confirmed AA
PENN064	AA	33.2	abnormalities_since_childhood	8.9	1.86	SAA/VSAA	NA	≥1%	.	PNH	.	.	Normal	Normal	0	Not done	Yes	Refractory	Yes	7.36	Dead	IBMFS genetics not tested AND no response/not evaluable resonse/no IST given BUT a clinical diagnosis of AA	Presumed AA
PENN065	AA	76.5	no_prior	no_prior	0.21	SAA/VSAA	≥1%	≥1%	PNH	PNH	.	.	NA	NA	NA	Not done	Yes	CR	No	6.33	Alive	IBMFS genetics not tested, BUT responded to IST (PR or CR)	Confirmed AA
PENN066	AA	56.1	normal_exists	exists_NA	0.12	SAA/VSAA	0.00	0.00	.	.	.	.	NA	NA	NA	Not done	Yes	PR	No	10.32	Alive	IBMFS genetics not tested, BUT responded to IST (PR or CR)	Confirmed AA
PENN067	IBMFS	22.2	abnormalities_since_childhood	lifelong	20.90	NSAA	0.00	0.00	.	.	.	congenital abnormality	Normal	Normal	0	Done	No	Not Applicable-not treated with IST	Yes	11.32	Alive	Confirmed IBMFS genetic and/or functional test (breakage/telomere) diagnostic of IBMFS	Confirmed IBMFS
PENN068	AA	75.1	normal_exists	0.3	0.03	SAA/VSAA	≥10%	≥10%	PNH	PNH	.	.	Normal	Normal	0	Not done	Yes	PR	Yes	2.20	Dead	IBMFS genetics not tested AND no response/not evaluable resonse/no IST given BUT a clinical diagnosis of AA	Confirmed AA
PENN069	AA	77.0	normal_exists	0.3	0.23	SAA/VSAA	0.00	0.00	.	.	Use of checkpoint inhibitors, osimertinib	.	NA	NA	NA	Not done	Yes	CR	Yes	3.01	Dead	IBMFS genetics not tested, BUT responded to IST (PR or CR)	Confirmed AA
PENN070	AA	82.1	normal_exists	0.8	0.01	NSAA	0.00	0.00	.	.	.	.	NA	NA	NA	Not done	Yes	Not Applicable-died or transplanted before 6 mo	Yes	0.51	Dead	IBMFS genetics not tested AND no response/not evaluable resonse/no IST given BUT a clinical diagnosis of AA	Presumed AA
PENN071	AA	69.1	no_prior	no_prior	0.04	SAA/VSAA	NA	NA	.	.	.	.	NA	NA	NA	Not done	Yes	PR	Yes	3.70	Alive	IBMFS genetics not tested, BUT responded to IST (PR or CR)	Confirmed AA
PENN072	AA	75.7	normal_exists	1.0	0.04	SAA/VSAA	0.00	0.00	.	6pLOH	.	.	NA	NA	NA	Not done	Yes	PR	Yes	9.36	Alive	IBMFS genetics not tested, BUT responded to IST (PR or CR)	Confirmed AA
PENN073	IBMFS	51.5	normal_exists	1.4	1.31	NSAA	NA	NA	.	.	.	ILD	Under1	Under1	NA	Done	No	Not Applicable-not treated with IST	Yes	3.96	Alive	Confirmed IBMFS genetic and/or functional test (breakage/telomere) diagnostic of IBMFS	Confirmed IBMFS
PENN074	AA	67.8	no_prior	no_prior	0.12	SAA/VSAA	0.00	0.00	.	.	.	.	NA	NA	NA	Not done	Yes	PR	Yes	8.27	Alive	IBMFS genetics not tested, BUT responded to IST (PR or CR)	Confirmed AA
PENN075	AA	47.0	no_prior	no_prior	0.04	SAA/VSAA	0.00	0.00	.	.	.	.	NA	NA	NA	Not done	Yes	PR	Yes	10.31	Alive	IBMFS genetics not tested AND no response/not evaluable resonse/no IST given BUT a clinical diagnosis of AA	Presumed AA
PENN076	AA	58.6	no_prior	no_prior	0.66	SAA/VSAA	small <0.5%	≥1%	.	PNH	.	.	Normal	NA	0	Not done	Yes	PR	No	5.32	Alive	IBMFS genetics not tested, BUT responded to IST (PR or CR)	Confirmed AA
PENN077	AA	24.9	normal_exists	1.8	0.01	SAA/VSAA	NA	small <0.5%	.	BCOR	.	neurologic abnormalities, autism-spectrum	Normal	Normal	0	Done	Yes	CR	Yes	2.51	Dead	IBMFS genetic testing negative AND responded to IST	Confirmed AA
PENN078	AA	20.3	no_prior	no_prior	0.00	SAA/VSAA	≥10%	≥10%	PNH	PNH	.	.	Between1and10	Under1	0	Not done	No	Not Applicable-not treated with IST	Yes	7.82	Alive	IBMFS genetics not tested AND no response/not evaluable resonse/no IST given BUT a clinical diagnosis of AA	Presumed AA
PENN079	AA	25.8	no_prior	no_prior	0.04	SAA/VSAA	NA	NA	.	.	.	asymmetric thumbs	Normal	NA	0	Done	No	Not Applicable-not treated with IST	Yes	5.64	Alive	IBMFS genetic testing negative	Confirmed AA

PENN080	AA	34.3	no_prior	no_prior	0.00	SAA/VSAA	0.00	0.00	.	.	.	.	Normal	Under1	0	Not done	Yes	PR	No	1.57	Dead	IBMFS genetics not tested, BUT responded to IST (PR or CR)	Confirmed AA
PENN081	IBMFS	32.3	abnormalities_since_childhood	no_prior	13.73	NSAA	0.00	0.00	.	.	.	.	Under1	Under1	0	Done	No	Not Applicable-not treated with IST	No	2.61	Alive	Confirmed IBMFS genetic and/or functional test (breakage/telomere) diagnostic of IBMFS	Presumed IBMFS NOS
PENN082	AA	42.1	normal_exists	6.6	0.30	NSAA	≥1%	≥1%	PNH	PNH	.	.	Normal	Normal	NA	Not done	Yes	Not Applicable-died or transplanted before 6 mo	No	1.40	Alive	IBMFS genetics not tested AND no response/not evaluable resonse/no IST given BUT a clinical diagnosis of AA	Presumed AA
PENN083	AA	69.0	no_prior	no_prior	0.03	SAA/VSAA	≥10%	≥10%	PNH	PNH	.	.	NA	NA	NA	Not done	Yes	PR	No	4.81	Dead	IBMFS genetics not tested, BUT responded to IST (PR or CR)	Confirmed AA
PENN084	AA	67.8	normal_exists	0.9	0.02	NSAA	small <0.5%	small <0.5%	.	.	.	.	NA	NA	NA	Not done	Yes	CR	No	1.60	Alive	IBMFS genetics not tested, BUT responded to IST (PR or CR)	Confirmed AA
PENN085	AA	66.7	no_prior	no_prior	0.01	SAA/VSAA	0.00	0.00	.	.	.	.	NA	NA	NA	Not done	Yes	PR	No	0.63	Alive	IBMFS genetics not tested, BUT responded to IST (PR or CR)	Confirmed AA
PENN086	AA	81.7	no_prior	no_prior	0.01	NSAA	≥10%	≥10%	PNH	PNH	.	.	NA	NA	NA	Not done	Yes	PR	Yes	1.64	Alive	IBMFS genetics not tested, BUT responded to IST (PR or CR)	Confirmed AA
PENN087	AA	50.3	normal_exists	1.4	0.08	SAA/VSAA	0.00	0.00	.	del 13q	.	.	NA	NA	NA	Not done	Yes	PR	Yes	17.14	Alive	IBMFS genetics not tested, BUT responded to IST (PR or CR)	Confirmed AA
PENN088	AA	66.1	normal_exists	1.1	0.16	SAA/VSAA	0.00	0.00	.	del 13q	.	.	NA	NA	NA	Not done	Yes	Refractory	No	9.20	Alive	IBMFS genetics not tested, BUT responded to IST (PR or CR)	Confirmed AA
PENN089	AA	27.1	normal_exists	0.1	0.11	SAA/VSAA	0.00	0.00	.	.	hepatitis	.	Normal	NA	NA	Not done	Yes	Not Applicable-died or transplanted before 6 mo	No	0.49	Dead	IBMFS genetics not tested, BUT responded to IST (PR or CR)	Presumed AA
PENN090	AA	19.3	normal_exists	1.2	0.01	SAA/VSAA	0.00	0.00	.	.	.	.	Normal	NA	0	Not done	Yes	CR	No	1.23	Alive	IBMFS genetics not tested, BUT responded to IST (PR or CR)	Confirmed AA
PENN091	AA	26.2	no_prior	no_prior	0.04	SAA/VSAA	≥1%	≥1%	PNH, 6pLOH	6pLOH	.	.	Normal	Under1	0	Not done	Yes	PR	No	0.71	Alive	IBMFS genetics not tested, BUT responded to IST (PR or CR)	Confirmed AA
PENN092	AA	54.7	normal_exists	1.4	0.38	NSAA	0.00	0.00	.	.	.	.	Normal	NA	0	Not done	Yes	PR	No	5.69	Alive	IBMFS genetics not tested, BUT responded to IST (PR or CR)	Confirmed AA
PENN093	IBMFS	70.1	normal_exists	2.7	2.54	NSAA	NA	NA	.	.	.	ILD	Between1and10	Under1	NA	Done	No	Not Applicable-not treated with IST	No	1.71	Dead	No genetic diagnosis of IBMFS, but functional and clinical data suggestive of IBMFS	Presumed IBMFS NOS
PENN094	AA	59.7	normal_exists	3.0	0.51	NSAA	small <0.5%	small <0.5%	.	.	.	.	Normal	Between1and10	0	Not done	Yes	CR	No	2.18	Alive	IBMFS genetics not tested, BUT responded to IST (PR or CR)	Confirmed AA
PENN095	IBMFS	72.4	abnormalities_since_childhood	no_prior	1.35	NSAA	na	na	.	.	.	ILD, rectal cancer, tongue cancer	Under1	Under1	NA	Done	No	Not Applicable-not treated with IST	No	0.95	Dead	Confirmed IBMFS genetic and/or functional test (breakage/telomere) diagnostic of IBMFS	Confirmed IBMFS
PENN096	AA	26.8	normal_exists	0.4	0.12	SAA/VSAA	0.00	0.00	.	.	.	.	Normal	NA	0	Not done	Yes	Refractory	Yes	1.78	Alive	IBMFS genetics not tested AND no response/not evaluable resonse/no IST given BUT a clinical diagnosis of AA	Presumed AA
PENN097	AA	49.4	no_prior	no_prior	0.21	SAA/VSAA	NA	≥1%	.	PNH	.	.	NA	NA	NA	Not done	Yes	PR	Yes	9.49	Alive	IBMFS genetics not tested, BUT responded to IST (PR or CR)	Confirmed AA
PENN098	AA	86.7	normal_exists	0.7	0.14	SAA/VSAA	0.00	0.00	.	.	.	.	NA	NA	NA	Not done	Yes	PR	No	3.84	Alive	IBMFS genetics not tested, BUT responded to IST (PR or CR)	Confirmed AA
PENN099	AA	51.9	normal_exists	1.7	0.11	SAA/VSAA	0.5 to <1%	≥1%	PNH	PNH	.	.	NA	NA	NA	Not done	Yes	CR	No	4.24	Alive	IBMFS genetics not tested, BUT responded to IST (PR or CR)	Confirmed AA
PENN100	IBMFS	33.5	abnormalities_since_childhood	lifelong	33.55	SAA/VSAA	0.00	0.00	6pLOH	6pLOH	.	congenital abnormality	Normal	Under1	0	Done	Yes	Refractory	No	12.84	Alive	Confirmed IBMFS genetic and/or functional test (breakage/telomere) diagnostic of IBMFS	Confirmed IBMFS
PENN101	AA	76.4	normal_exists	0.5	0.12	SAA/VSAA	0	0	.	.	.	.	NA	NA	NA	Not done	Yes	PR	No	6.72	Alive	IBMFS genetics not tested, BUT responded to IST (PR or CR)	Confirmed AA
PENN102	AA	58.1	normal_exists	0.2	0.01	SAA/VSAA	0.00	0.00	.	.	.	.	Between1and10	Under1	NA	Not done	Yes	CR	No	7.71	Dead	IBMFS genetics not tested, BUT responded to IST (PR or CR)	Confirmed AA
PENN103	AA	64.9	normal_exists	0.5	0.36	SAA/VSAA	0.00	0.00	.	BCOR	.	.	Normal	Normal	0	Not done	Yes	Refractory	No	3.36	Alive	IBMFS genetics not tested, BUT responded to IST (PR or CR)	Confirmed AA
PENN104	AA	61.2	normal_exists	exists_NA	0.05	SAA/VSAA	0.00	0.00	.	.	.	.	NA	NA	NA	Not done	Yes	CR	No	10.81	Alive	IBMFS genetics not tested, BUT responded to IST (PR or CR)	Confirmed AA
PENN105	IBMFS	69.8	normal_exists	0.8	0.65	NSAA	NA	NA	.	.	.	ILD	Between1and10	Under1	NA	Done	No	Not Applicable-not treated with IST	No	4.75	Alive	No genetic diagnosis of IBMFS, but functional and clinical data suggestive of IBMFS	Confirmed IBMFS
PENN106	AA	22.4	normal_exists	5.7	0.36	SAA/VSAA	≥1%	≥1%	PNH	PNH	.	.	NA	NA	NA	Not done	Yes	Not Applicable-died or transplanted before 6 mo	Yes	4.90	Alive	IBMFS genetics not tested AND no response/not evaluable resonse/no IST given BUT a clinical diagnosis of AA	Presumed AA

PENN107	AA	29.0	abnormalities_since_childhood	no_prior	11.65	NSAA	0.00	0.00	.	.	.	.	Between1and10	Under1	0	Done	Yes	PR	No	0.29	Alive	IBMFS genetic testing negative	Presumed AA
PENN108	AA	44.3	normal_exists	0.7	0.44	SAA/VSAA	0.00	0.00	.	.	.	.	Normal	NA	0	Done	Yes	PR	No	5.75	Alive	IBMFS genetic testing negative	Confirmed AA
PENN109	AA	48.8	no_prior	no_prior	4.90	SAA/VSAA	≥10%	≥10%	PNH	PNH	.	.	Normal	Under1	NA	Not done	Yes	PR	No	3.78	Alive	IBMFS genetics not tested, BUT responded to IST (PR or CR)	Confirmed AA
PENN110	IBMFS	35.7	no_prior	no_prior	0.87	NSAA	NA	NA	.	.	.	cirrhosis	Under1	Under1	NA	Done	No	Not Applicable-not treated with IST	No	9.66	Alive	Confirmed IBMFS genetic and/or functional test (breakage/telomere) diagnostic of IBMFS	Confirmed IBMFS
PENN111	AA	21.1	no_prior	no_prior	0.35	NSAA	0.00	0.00	.	.	.	.	Normal	Between1and10	0	Done	No	Not Applicable-not treated with IST	No	0.08	Alive	IBMFS genetic testing negative	Presumed AA
PENN112	AA	19.4	no_prior	no_prior	0.09	SAA/VSAA	≥1%	≥10%	PNH	PNH	.	.	Normal	NA	0	Not done	Yes	Refractory	Yes	8.72	Alive	IBMFS genetics not tested AND no response/not evaluable resonse/no IST given BUT a clinical diagnosis of AA	Presumed AA
PENN113	AA	67.1	normal_exists	0.7	0.00	SAA/VSAA	0.5 to <1%	0.5 to <1%	PNH	PNH	.	.	NA	NA	NA	Not done	Yes	CR	No	3.02	Alive	IBMFS genetics not tested, BUT responded to IST (PR or CR)	Confirmed AA
PENN114	AA	61.0	normal_exists	3.1	0.03	NSAA	0.00	small <0.5%	.	BCOR	thymoma	.	NA	NA	0	Not done	Yes	CR	No	4.47	Alive	IBMFS genetics not tested, BUT responded to IST (PR or CR)	Confirmed AA
PENN115	AA	50.6	normal_exists	3.5	0.00	SAA/VSAA	small <0.5%	≥1%	.	PNH, BCOR	.	.	Normal	NA	NA	Not done	Yes	PR	No	11.04	Alive	IBMFS genetics not tested, BUT responded to IST (PR or CR)	Confirmed AA
PENN116	AA	52.7	no_prior	no_prior	0.59	NSAA	≥1%	≥1%	PNH	PNH, BCOR	.	.	Normal	Between1and10	0	Not done	Yes	CR	No	3.39	Alive	IBMFS genetics not tested, BUT responded to IST (PR or CR)	Confirmed AA
PENN117	AA	32.0	no_prior	no_prior	0.01	SAA/VSAA	0.00	NA	.	.	.	.	Normal	NA	0	Not done	No	Not Applicable-not treated with IST	Yes	7.18	Alive	IBMFS genetics not tested, BUT responded to IST (PR or CR)	Confirmed AA
PENN118	AA	56.6	normal_exists	7.2	0.61	SAA/VSAA	≥1%	≥1%	PNH	PNH	.	.	NA	NA	NA	Not done	Yes	PR	No	7.05	Alive	IBMFS genetics not tested, BUT responded to IST (PR or CR)	Confirmed AA
PENN119	AA	48.8	normal_exists	0.6	0.01	SAA/VSAA	0.00	0.00	.	.	Hodgkins	.	NA	NA	NA	Not done	Yes	Refractory	Yes	1.06	Dead	IBMFS genetics not tested AND no response/not evaluable resonse/no IST given BUT a clinical diagnosis of AA	Presumed AA
PENN120	AA	76.1	normal_exists	0.5	0.00	SAA/VSAA	0.00	0.00	.	.	.	.	NA	NA	NA	Not done	Yes	Refractory	No	0.88	Dead	IBMFS genetics not tested AND no response/not evaluable resonse/no IST given BUT a clinical diagnosis of AA	Presumed AA
PENN121	AA	41.1	normal_exists	0.8	0.05	SAA/VSAA	NA	≥1%	.	PNH	.	.	Normal	NA	0	Not done	Yes	CR	No	9.27	Alive	IBMFS genetics not tested, BUT responded to IST (PR or CR)	Confirmed AA
PENN122	AA	65.8	normal_exists	1.9	0.67	NSAA	small <0.5%	≥1%	.	PNH	.	.	NA	NA	NA	Not done	Yes	PR	No	6.20	Alive	IBMFS genetics not tested, BUT responded to IST (PR or CR)	Confirmed AA
PENN123	AA	22.9	no_prior	no_prior	0.01	SAA/VSAA	0.00	0.00	.	.	.	.	Normal	Normal	0	Not done	No	Not Applicable-not treated with IST	Yes	3.13	Alive	IBMFS genetics not tested AND no response/not evaluable resonse/no IST given BUT a clinical diagnosis of AA	Presumed AA
PENN124	AA	69.6	normal_exists	4.1	0.08	SAA/VSAA	small <0.5%	small <0.5%	.	.	.	.	NA	NA	NA	Not done	Yes	CR	No	3.25	Alive	IBMFS genetics not tested, BUT responded to IST (PR or CR)	Confirmed AA
PENN125	IBMFS	22.5	abnormalities_since_childhood	no_prior	4.01	NSAA	NA	NA	.	.	.	IPF, cirrhosis, congenital defect	Under1	Under1	NA	Done	No	Not Applicable-not treated with IST	No	11.76	Alive	Confirmed IBMFS genetic and/or functional test (breakage/telomere) diagnostic of IBMFS	Confirmed IBMFS
PENN126	AA	76.7	normal_exists	0.6	0.18	SAA/VSAA	≥1%	≥1%	PNH	PNH	.	.	NA	NA	NA	Not done	Yes	PR	No	1.67	Alive	IBMFS genetics not tested, BUT responded to IST (PR or CR)	Confirmed AA
PENN127	AA	73.7	no_prior	no_prior	0.18	SAA/VSAA	≥10%	≥10%	PNH	PNH	.	.	NA	NA	NA	Not done	Yes	CR	No	2.20	Dead	IBMFS genetics not tested, BUT responded to IST (PR or CR)	Confirmed AA
PENN128	AA	55.8	normal_exists	4.5	0.81	SAA/VSAA	≥10%	≥10%	PNH, 6pLOH	6pLOH, BCOR, del 13q	.	.	NA	NA	NA	Not done	Yes	PR	No	1.11	Alive	IBMFS genetics not tested, BUT responded to IST (PR or CR)	Confirmed AA
PENN129	IBMFS	34.9	abnormalities_since_childhood	lifelong	21.09	NSAA	0.00	0.00	.	.	.	brother with BMF	Under1	Under1	0	Done	No	Not Applicable-not treated with IST	No	3.87	Alive	No genetic diagnosis of IBMFS, but functional and clinical data suggestive of IBMFS	Presumed IBMFS NOS
PENN130	AA	68.8	no_prior	no_prior	1.54	SAA/VSAA	0.00	0.00	.	.	.	.	NA	NA	0	Not done	Yes	Refractory	Yes	5.48	Alive	IBMFS genetics not tested AND no response/not evaluable resonse/no IST given BUT a clinical diagnosis of AA	Confirmed AA
PENN131	AA	75.6	normal_exists	0.6	0.08	SAA/VSAA	small <0.5%	small <0.5%	.	.	.	.	NA	NA	NA	Not done	Yes	PR	No	1.33	Dead	IBMFS genetics not tested AND no response/not evaluable resonse/no IST given BUT a clinical diagnosis of AA	Presumed AA
PENN132	AA	61.8	normal_exists	10.9	1.56	SAA/VSAA	0.5 to <1%	0.5 to <1%	PNH	PNH	.	.	NA	NA	NA	Not done	Yes	PR	No	2.76	Alive	IBMFS genetics not tested, BUT responded to IST (PR or CR)	Confirmed AA
PENN133	AA	60.8	normal_exists	1.3	0.01	SAA/VSAA	0.00	≥1%	.	PNH	.	.	NA	NA	NA	Not done	Yes	PR	No	2.95	Alive	IBMFS genetics not tested, BUT responded to IST (PR or CR)	Confirmed AA

PENN134	AA	72.0	normal_exists	1.3	0.32	NSAA	≥1%	≥10%	PNH	PNH	.	.	NA	NA	NA	Not done	Yes	PR	No	2.40	Dead	IBMFS genetics not tested, BUT responded to IST (PR or CR)	Confirmed AA
PENN135	AA	52.8	normal_exists	19.5	0.32	NSAA	≥10%	≥10%	PNH	PNH	.	.	NA	NA	NA	Not done	No	Not Applicable-not treated with IST	No	5.31	Alive	IBMFS genetics not tested, BUT responded to IST (PR or CR)	Confirmed AA
PENN136	AA	57.1	no_prior	no_prior	0.00	NSAA	0.00	0.00	.	.	.	.	NA	NA	NA	Not done	Yes	PR	No	14.32	Alive	IBMFS genetics not tested, BUT responded to IST (PR or CR)	Confirmed AA
PENN137	AA	22.7	no_prior	no_prior	0.01	SAA/VSAA	0.00	small <0.5%	.	BCOR	.	.	Normal	Between1and10	0	Not done	Yes	PR	No	9.04	Alive	IBMFS genetics not tested, BUT responded to IST (PR or CR)	Confirmed AA
PENN138	AA	61.0	no_prior	no_prior	0.00	SAA/VSAA	NA	NA	.	.	.	.	NA	NA	NA	Not done	Yes	PR	No	18.55	Dead	IBMFS genetics not tested, BUT responded to IST (PR or CR)	Presumed AA
PENN139	AA	79.5	normal_exists	1.8	0.89	SAA/VSAA	small <0.5%	NA	.	.	.	.	NA	NA	NA	Not done	Yes	PR	No	0.58	Dead	IBMFS genetics not tested AND no response/not evaluable resonse/no IST given BUT a clinical diagnosis of AA	Confirmed AA
PENN140	AA	62.2	normal_exists	3.0	0.11	NSAA	0.00	0.00	.	BCOR	.	.	Normal	NA	NA	Not done	Yes	PR	No	1.94	Alive	IBMFS genetics not tested, BUT responded to IST (PR or CR)	Confirmed AA
PENN141	AA	42.2	normal_exists	2.2	5.08	NSAA	0.00	0.00	.	.	Previously tolerated chemotherapy or radiation	.	NA	NA	NA	Not done	Yes	CR	No	1.30	Alive	IBMFS genetics not tested, BUT responded to IST (PR or CR)	Confirmed AA
PENN142	AA	26.6	no_prior	no_prior	0.29	NSAA	≥1%	≥1%	PNH	PNH	.	.	Between1and10	Under1	0	Not done	Yes	CR	No	0.30	Alive	IBMFS genetics not tested, BUT responded to IST (PR or CR)	Confirmed AA
PENN143	AA	30.0	no_prior	no_prior	0.04	SAA/VSAA	0.00	0.00	BCOR	BCOR	.	.	Between1and10	Normal	0	Not done	Yes	CR	No	0.82	Alive	IBMFS genetics not tested, BUT responded to IST (PR or CR)	Confirmed AA
PENN144	IBMFS	33.3	no_prior	no_prior	9.04	NSAA	NA	NA	.	.	.	AVN, father with IPF	Under1	Under1	NA	Done	No	Not Applicable-not treated with IST	No	3.31	Alive	Confirmed IBMFS genetic and/or functional test (breakage/telomere) diagnostic of IBMFS	Confirmed IBMFS
PENN145	IBMFS	60.4	normal_exists	1.3	1.10	NSAA	0.00	0.00	.	.	.	IPF, family history of BMF and IPF	Under1	Under1	NA	Done	No	Not Applicable-not treated with IST	No	4.19	Dead	Confirmed IBMFS genetic and/or functional test (breakage/telomere) diagnostic of IBMFS	Confirmed IBMFS
PENN146	AA	49.3	normal_exists	2.2	0.75	SAA/VSAA	≥10%	≥10%	PNH, del13q	PNH, del 13q	.	.	Normal	Under1	0	Not done	Yes	PR	No	9.81	Alive	IBMFS genetics not tested, BUT responded to IST (PR or CR)	Confirmed AA
PENN147	IBMFS	57.4	no_prior	no_prior	2.82	NSAA	NA	NA	.	.	.	IPF, gray hair, mother leukemia	Under1	Under1	NA	Done	No	Not Applicable-not treated with IST	No	1.17	Alive	Confirmed IBMFS genetic and/or functional test (breakage/telomere) diagnostic of IBMFS	Confirmed IBMFS
PENN148	AA	64.7	normal_exists	0.7	0.02	SAA/VSAA	small <0.5%	small <0.5%	.	.	.	.	NA	NA	NA	Not done	Yes	PR	No	2.25	Alive	IBMFS genetics not tested, BUT responded to IST (PR or CR)	Confirmed AA
PENN149	AA	56.0	normal_exists	0.7	0.07	SAA/VSAA	0.00	0.00	.	.	.	.	Normal	NA	0	Not done	Yes	Not Applicable-died or transplanted before 6 mo	No	0.06	Dead	IBMFS genetics not tested, BUT responded to IST (PR or CR)	Confirmed AA
PENN150	AA	33.5	normal_exists	2.7	0.12	SAA/VSAA	0.5 to <1%	0.5 to <1%	PNH	PNH	.	.	Normal	Normal	0	Not done	No	Not Applicable-not treated with IST	Yes	3.05	Alive	IBMFS genetics not tested AND no response/not evaluable resonse/no IST given BUT a clinical diagnosis of AA	Confirmed AA
PENN151	AA	61.1	normal_exists	1.2	0.00	SAA/VSAA	≥1%	≥1%	PNH	PNH	.	.	NA	NA	NA	Not done	Yes	PR	No	5.48	Alive	IBMFS genetics not tested, BUT responded to IST (PR or CR)	Confirmed AA
PENN152	AA	48.1	no_prior	no_prior	0.06	SAA/VSAA	NA	NA	.	.	.	.	NA	NA	NA	Not done	Yes	PR	No	8.86	Alive	IBMFS genetics not tested, BUT responded to IST (PR or CR)	Confirmed AA
PENN153	IBMFS	62.0	normal_exists	10.0	9.01	NSAA	NA	NA	.	.	.	ILD, fam hx ILD	Under1	Under1	NA	Done	No	Not Applicable-not treated with IST	No	9.53	Alive	Confirmed IBMFS genetic and/or functional test (breakage/telomere) diagnostic of IBMFS	Confirmed IBMFS
PENN154	AA	25.3	no_prior	no_prior	0.01	SAA/VSAA	NA	≥10%	.	PNH	.	.	NA	NA	NA	Not done	Yes	PR	No	15.11	Alive	IBMFS genetics not tested, BUT responded to IST (PR or CR)	Confirmed AA
PENN155	AA	63.4	no_prior	no_prior	0.67	SAA/VSAA	≥1%	≥1%	PNH, 6pLOH	6pLOH	.	.	NA	NA	NA	Not done	Yes	CR	No	9.89	Alive	IBMFS genetics not tested, BUT responded to IST (PR or CR)	Confirmed AA
PENN156	AA	44.8	no_prior	no_prior	0.08	SAA/VSAA	small <0.5%	≥1%	.	PNH, del 13q, BCORL1	.	.	Normal	Between1and10	0	Not done	Yes	CR	No	4.39	Alive	IBMFS genetics not tested, BUT responded to IST (PR or CR)	Confirmed AA
PENN157	AA	61.2	normal_exists	1.8	0.09	SAA/VSAA	≥1%	small <0.5%	PNH	BCORL1	.	.	Between1and10	Under1	NA	Not done	Yes	CR	No	7.21	Dead	IBMFS genetics not tested, BUT responded to IST (PR or CR)	Confirmed AA
PENN158	AA	47.8	normal_exists	4.1	0.05	SAA/VSAA	0.00	≥1%	.	PNH	.	.	NA	NA	NA	Not done	Yes	PR	No	9.22	Alive	IBMFS genetics not tested, BUT responded to IST (PR or CR)	Confirmed AA
PENN159	AA	38.6	normal_exists	1.4	0.00	SAA/VSAA	≥1%	0.5 to <1%	PNH	PNH	.	.	Normal	NA	0	Not done	Yes	PR	No	3.43	Alive	IBMFS genetics not tested, BUT responded to IST (PR or CR)	Confirmed AA
PENN160	AA	63.2	no_prior	no_prior	0.02	SAA/VSAA	0.00	0.00	.	.	.	.	NA	NA	NA	Not done	Yes	PR	No	2.72	Dead	IBMFS genetics not tested, BUT responded to IST (PR or CR)	Confirmed AA
PENN161	AA	22.1	no_prior	no_prior	0.09	SAA/VSAA	na	≥10%	.	PNH, BCOR	.	.	NA	NA	NA	Not done	Yes	CR	No	14.18	Alive	IBMFS genetics not tested, BUT responded to IST (PR or CR)	Confirmed AA

PENN162	IBMFS	52.2	abnormalities_since_childhood	no_prior	7.84	NSAA	NA	NA	.	.	.	ILD	Under1	Under1	NA	Done	No	Not Applicable-not treated with IST	No	0.70	Dead	Confirmed IBMFS genetic and/or functional test (breakage/telomere) diagnostic of IBMFS	Confirmed IBMFS
PENN163	AA	23.8	no_prior	no_prior	0.00	SAA/VSAA	≥1%	≥1%	PNH	PNH	.	.	Normal	Normal	0	Not done	Yes	CR	No	1.66	Alive	IBMFS genetics not tested, BUT responded to IST (PR or CR)	Confirmed AA
PENN164	AA	50.8	no_prior	no_prior	0.00	NSAA	small <0.5%	NA	.	BCOR	.	.	Normal	Between1and10	0	Not done	Yes	CR	No	1.00	Alive	IBMFS genetics not tested, BUT responded to IST (PR or CR)	Confirmed AA
PENN165	AA	54.0	normal_exists	0.3	0.16	SAA/VSAA	NA	NA	.	.	Previously tolerated chemotherapy or radiation	.	NA	NA	NA	Not done	Yes	CR	No	1.92	Dead	IBMFS genetics not tested, BUT responded to IST (PR or CR)	Confirmed AA
PENN166	IBMFS	60.5	abnormalities_since_childhood	no_prior	8.52	NSAA	0.00	0.00	.	.	.	ILD, cirrhosis	Between1and10	Under1	NA	Done	No	Not Applicable-not treated with IST	No	5.35	Dead	No genetic diagnosis of IBMFS, but functional and clinical data suggestive of IBMFS	Confirmed IBMFS
PENN167	AA	20.5	no_prior	no_prior	3.01	SAA/VSAA	NA	≥1%	.	PNH	.	.	Between1and10	Under1	0	Not done	Yes	PR	No	18.36	Alive	IBMFS genetics not tested, BUT responded to IST (PR or CR)	Confirmed AA
PENN168	AA	66.8	no_prior	no_prior	0.04	SAA/VSAA	≥1%	small <0.5%	PNH	.	.	.	NA	NA	NA	Not done	Yes	PR	No	5.60	Alive	IBMFS genetics not tested, BUT responded to IST (PR or CR)	Confirmed AA
PENN169	AA	28.5	no_prior	no_prior	0.02	SAA/VSAA	≥1%	≥1%	PNH	PNH	.	.	Normal	Normal	0	Not done	Yes	PR	No	1.71	Alive	IBMFS genetics not tested, BUT responded to IST (PR or CR)	Confirmed AA
PENN170	AA	50.8	normal_exists	27.3	1.93	SAA/VSAA	small <0.5%	NA	.	.	.	tongue cancer, CVID, mother glioblastoma	Between1and10	NA	0	Done	Yes	Refractory	No	0.31	Dead	IBMFS genetic testing negative	Presumed AA
PENN171	AA	51.3	no_prior	no_prior	0.01	SAA/VSAA	0.00	NA	.	.	.	.	NA	NA	NA	Not done	Yes	PR	No	8.25	Alive	IBMFS genetics not tested, BUT responded to IST (PR or CR)	Confirmed AA
PENN172	AA	58.0	no_prior	no_prior	0.04	SAA/VSAA	0.5 to <1%	≥1%	PNH	PNH	.	.	NA	NA	NA	Not done	Yes	PR	Yes	8.78	Dead	IBMFS genetics not tested, BUT responded to IST (PR or CR)	Presumed AA
PENN173	AA	63.7	normal_exists	0.5	0.08	SAA/VSAA	0.00	≥10%	.	PNH	.	.	NA	NA	NA	Not done	Yes	PR	No	6.12	Dead	IBMFS genetics not tested, BUT responded to IST (PR or CR)	Confirmed AA
PENN174	IBMFS	20.6	no_prior	no_prior	1.74	NSAA	0.00	NA	.	.	.	.	Normal	Under1	1	Done	No	Not Applicable-not treated with IST	No	5.24	Alive	Confirmed IBMFS genetic and/or functional test (breakage/telomere) diagnostic of IBMFS	Confirmed IBMFS
PENN175	AA	75.3	normal_exists	2.2	0.34	SAA/VSAA	NA	small <0.5%	.	.	.	.	NA	NA	NA	Not done	Yes	Refractory	No	3.82	Alive	IBMFS genetics not tested AND no response/not evaluable resonse/no IST given BUT a clinical diagnosis of AA	Presumed AA
PENN176	AA	62.5	no_prior	no_prior	0.53	SAA/VSAA	≥1%	≥10%	PNH	PNH, BCOR, del 13q	.	.	NA	NA	NA	Not done	Yes	PR	No	8.93	Alive	IBMFS genetics not tested, BUT responded to IST (PR or CR)	Confirmed AA
PENN177	AA	27.4	normal_exists	6.2	0.10	SAA/VSAA	≥10%	≥10%	PNH, BCOR	PNH	hepatitis	.	Normal	Between1and10	0	Not done	Yes	Refractory	No	1.49	Alive	IBMFS genetics not tested AND no response/not evaluable resonse/no IST given BUT a clinical diagnosis of AA	Presumed AA
PENN178	AA	52.6	normal_exists	2.9	1.13	NSAA	≥1%	≥1%	PNH	PNH, BCOR	.	.	Normal	Between1and10	0	Not done	Yes	CR	No	7.41	Alive	IBMFS genetics not tested, BUT responded to IST (PR or CR)	Confirmed AA
PENN179	AA	22.5	no_prior	no_prior	0.42	SAA/VSAA	NA	≥10%	.	PNH	.	.	Between1and10	Under1	NA	Not done	Yes	PR	No	14.67	Alive	IBMFS genetics not tested, BUT responded to IST (PR or CR)	Confirmed AA
PENN180	AA	65.3	no_prior	no_prior	0.05	SAA/VSAA	≥10%	≥10%	PNH	PNH	.	.	NA	NA	NA	Not done	Yes	PR	No	3.89	Alive	IBMFS genetics not tested, BUT responded to IST (PR or CR)	Confirmed AA
PENN181	AA	51.6	normal_exists	1.0	0.23	NSAA	small <0.5%	small <0.5%	.	.	.	.	NA	NA	NA	Not done	Yes	CR	No	13.09	Alive	IBMFS genetics not tested AND no response/not evaluable resonse/no IST given BUT a clinical diagnosis of AA	Confirmed AA
PENN182	AA	46.9	no_prior	no_prior	0.00	NSAA	0.00	0.00	6pLOH	6pLOH	.	.	Normal	Between1and10	0	Not done	Yes	PR	No	12.87	Alive	IBMFS genetics not tested, BUT responded to IST (PR or CR)	Confirmed AA
PENN183	AA	53.4	no_prior	no_prior	0.00	SAA/VSAA	≥1%	≥1%	PNH	PNH	.	.	Normal	Under1	NA	Not done	Yes	CR	No	5.57	Alive	IBMFS genetics not tested, BUT responded to IST (PR or CR)	Confirmed AA
PENN184	AA	78.7	normal_exists	0.2	0.12	SAA/VSAA	0.00	0.00	del13q	del 13q	.	.	NA	NA	NA	Not done	Yes	Refractory	No	7.83	Alive	IBMFS genetics not tested AND no response/not evaluable resonse/no IST given BUT a clinical diagnosis of AA	Presumed AA
PENN185	AA	28.4	normal_exists	6.0	2.01	SAA/VSAA	0.00	0.00	.	.	.	.	Between1and10	Under1	0	Done	Yes	Refractory	No	20.74	Alive	IBMFS genetic testing negative	Confirmed AA
PENN186	AA	41.7	normal_exists	6.1	3.21	NSAA	0.00	0.00	6pLOH	6pLOH	eosinophilic fasciitis	.	Between1and10	Between1and10	0	Done	Yes	CR	No	3.22	Alive	IBMFS genetic testing negative	Confirmed AA
PENN187	AA	45.9	normal_exists	6.6	0.05	SAA/VSAA	≥10%	≥10%	PNH	PNH	.	.	Between1and10	Under1	0	Not done	Yes	PR	No	0.19	Alive	IBMFS genetics not tested, BUT responded to IST (PR or CR)	Confirmed AA
PENN188	AA	42.1	normal_exists	3.6	0.70	SAA/VSAA	0.00	0.00	BCOR	BCOR	.	.	Normal	NA	0	Done	Yes	Unknown	No	0.30	Alive	IBMFS genetic testing negative	Confirmed AA
PENN189	AA	25.0	normal_exists	1.4	0.16	SAA/VSAA	≥1%	≥10%	PNH	PNH	.	.	NA	NA	0	Not done	Yes	PR	No	0.48	Alive	IBMFS genetics not tested, BUT responded to IST (PR or CR)	Confirmed AA

PENN190	AA	21.5	no_prior	no_prior	1.04	NSAA	NA	0.00	.	.	.	.	Normal	Normal	0	Done	No	Not Applicable- not treated with IST	No	6.53	Alive	IBMFS genetic testing negative	Presumed AA
PENN191	AA	64.9	normal_exists	7.0	0.30	SAA/VSAA	≥10%	≥10%	PNH	PNH	.	.	NA	NA	NA	Not done	Yes	PR	No	0.81	Alive	IBMFS genetics not tested, BUT responded to IST (PR or CR)	Confirmed AA
PENN192	AA	70.3	normal_exists	0.7	0.10	SAA/VSAA	small <0.5%	0.5 to <1%	PNH	PNH	Previously tolerated chemotherapy or radiation	.	Normal	NA	NA	Done	Yes	Not Applicable- died or transplanted before 6 mo	No	0.42	dead	IBMFS genetics not tested AND no response/not evaluable resonse/no IST given BUT a clinical diagnosis of AA	Presumed AA
PENN194	IBMFS	55.2	no_prior	lifelong	15.00	NSAA	NA	NA	.	.	.	IPF, AVN, family history	Under1	Under1	NA	Done	No	Not Applicable- not treated with IST	No	0.70	Dead	Confirmed IBMFS genetic and/or functional test (breakage/telomere) diagnostic of IBMFS	Confirmed IBMFS
PENN195	IBMFS	77.3	normal_exists	no_prior	6.10	NSAA	NA	NA	.	.	.	IPF	Between1and10	Between1and10	NA	Done	No	Not Applicable- not treated with IST	No	1.59	Alive	Confirmed IBMFS genetic and/or functional test (breakage/telomere) diagnostic of IBMFS	Confirmed IBMFS
PENN196	IBMFS	65.0	normal_exists	no_prior	0.80	NSAA	NA	NA	.	.	.	IPF	Under1	Under1	NA	Not done	No	Not Applicable- not treated with IST	No	0.03	Dead	No genetic diagnosis of IBMFS, but functional and clinical data suggestive of IBMFS	Confirmed IBMFS
PENN197	IBMFS	28.2	normal_exists	no_prior	0.90	NSAA	0	NA	.	.	.	.	Between1and10	Normal	0	Done	No	Not Applicable- not treated with IST	No	1.96	Alive	Confirmed IBMFS genetic and/or functional test (breakage/telomere) diagnostic of IBMFS	Confirmed IBMFS
PENN198	IBMFS	67.8	no_prior	no_prior	lifelong	NSAA	NA	NA	.	.	.	Cirrhosis, IPF, 2 brothers with MDS or AML	Between1and10	Under1	NA	Done	No	Not Applicable- not treated with IST	No	3.82	Alive	Confirmed IBMFS genetic and/or functional test (breakage/telomere) diagnostic of IBMFS	Confirmed IBMFS
PENN199	IBMFS	34.5	no_prior	no_prior	16.00	NSAA	0	0	.	.	.	multiple bacterial infections- lymphedema - severe HPV - severe and persistent HSV FH son motor malformation	Normal	NA	0	Done	Yes	Refractory	Yes	13.49	Alive	Confirmed IBMFS genetic and/or functional test (breakage/telomere) diagnostic of IBMFS	Confirmed IBMFS
PENN200	IBMFS	30.4	no_prior	lifelong	lifelong	NSAA	NA	NA	.	.	.	small thumbs VSD; father with triphalangeal thumb	NA	NA	NA	Done	No	Not Applicable- not treated with IST	No	6.71	Alive	Confirmed IBMFS genetic and/or functional test (breakage/telomere) diagnostic of IBMFS	Confirmed IBMFS
PENN201	IBMFS	38.7	no_prior	no_prior	lifelong	NSAA	0	NA	.	.	.	small for age - thumb malformation café au lait spots and frequent infections	NA	NA	1	Done	No	Not Applicable- not treated with IST	No	1.91	Alive	Confirmed IBMFS genetic and/or functional test (breakage/telomere) diagnostic of IBMFS	Confirmed IBMFS
PENN202	IBMFS	33.8	no_prior	no_prior	lifelong	NSAA	NA	NA	.	.	.	IPF, Brother with short telomere	Under1	Under1	NA	Done	No	Not Applicable- not treated with IST	No	0.04	Dead	Confirmed IBMFS genetic and/or functional test (breakage/telomere) diagnostic of IBMFS	Confirmed IBMFS
PENN203	IBMFS	24.4	no_prior	no_prior	lifelong	NSAA	0	NA	.	.	.	.	Normal	Normal	0	Done	No	Not Applicable- not treated with IST	No	2.29	Alive	Confirmed IBMFS genetic and/or functional test (breakage/telomere) diagnostic of IBMFS	Confirmed IBMFS
PENN204	IBMFS	27.5	normal_exists	no_prior	0.00	NSAA	NA	NA	.	.	.	FH Wilms tumor, T-cell ALL, medulloblastoma, and AML in sister; personal history of colon cancer, BCC, and metastatic neuroendocrine cancer, and sensitivity to chemotherapy	NA	NA	1	Done	No	Not Applicable- not treated with IST	No	0.53	Dead	Confirmed IBMFS genetic and/or functional test (breakage/telomere) diagnostic of IBMFS	Confirmed IBMFS
PENN205	IBMFS	31.0	no_prior	no_prior	1.62	NSAA	NA	NA	.	.	.	.	Under1	NA	NA	Done	No	Not Applicable- not treated with IST	No	5.71	Alive	Confirmed IBMFS genetic and/or functional test (breakage/telomere) diagnostic of IBMFS	Confirmed IBMFS
PENN206	IBMFS	37.7	no_prior	no_prior	2.16	NSAA	NA	NA	.	.	.	brother with FA; patient had café au lait, hypoplastic thumb, dysplasia upper palate, SCC oropharynx	NA	NA	1	Done	No	Not Applicable- not treated with IST	No	1.61	Dead	Confirmed IBMFS genetic and/or functional test (breakage/telomere) diagnostic of IBMFS	Confirmed IBMFS
PENN207	IBMFS	22.5	no_prior	no_prior	4.41	NSAA	0	0	.	.	.	skeletal dysplasia, short stature	NA	NA	NA	Done	No	Not Applicable- not treated with IST	No	1.43	Alive	Confirmed IBMFS genetic and/or functional test (breakage/telomere) diagnostic of IBMFS	Confirmed IBMFS
PENN208	IBMFS	57.8	no_prior	no_prior	25.96	NSAA	NA	NA	.	.	.	Family hx - brother had leukemia; short stature	Under1	Under1	1	Done	No	Not Applicable- not treated with IST	No	0.14	Alive	Confirmed IBMFS genetic and/or functional test (breakage/telomere) diagnostic of IBMFS	Confirmed IBMFS
PENN209	IBMFS	58.5	no_prior	no_prior	15.51	NSAA	0	0	.	.	.	brother with chronic unexplained thrombocytopenia	Between1and10	Between1and10	0	Done	No	Not Applicable- not treated with IST	No	2.15	Dead	Confirmed IBMFS genetic and/or functional test (breakage/telomere) diagnostic of IBMFS	Confirmed IBMFS
PENN210	IBMFS	18.2	no_prior	no_prior	18.00	NSAA	NA	NA	.	.	.	VSD, ASD, and PDA- closed spontaneously. Neurodevelopmental delays, seizures; femoral retroversion; amblyopia, growth hormone deficiency.	Under1	Under1	NA	Done	No	Not Applicable- not treated with IST	No	5.33	Alive	Confirmed IBMFS genetic and/or functional test (breakage/telomere) diagnostic of IBMFS	Confirmed IBMFS
PENN211	IBMFS	28.6	no_prior	no_prior	14.30	NSAA	0	0	.	.	.	recurrent warts	NA	NA	0	Done	No	Not Applicable- not treated with IST	No	5.54	Alive	Confirmed IBMFS genetic and/or functional test (breakage/telomere) diagnostic of IBMFS	Confirmed IBMFS

PENN212	IBMFS	26.4	no_prior	no_prior	17.66	NSAA	NA	NA	.	.	.	dextrocardia	NA	NA	0	Done	No	Not Applicable-not treated with IST	No	1.36	Alive	Confirmed IBMFS genetic and/or functional test (breakage/telomere) diagnostic of IBMFS	Confirmed IBMFS
PENN213	IBMFS	25.4	no_prior	no_prior	23.98	NSAA	NA	NA	.	.	.	.	NA	NA	NA	Done	No	Not Applicable-not treated with IST	No	4.32	Alive	Confirmed IBMFS genetic and/or functional test (breakage/telomere) diagnostic of IBMFS	Confirmed IBMFS

Supplemental Dataset S2. Training cohort individual-level PASS calculation.

Age at BMF evaluation refers to the patient's age at the time of bone marrow failure assessment. Years from first abnormal complete blood count (CBC) to BMF diagnosis represents the interval between the initial abnormal blood count and confirmed diagnosis, with ≤1 year considered acute and >1 year chronic. Cytopenia severity is defined according to standard aplastic anemia (AA) criteria. The presence of any of the following at diagnosis—PNH clone ≥0.5% in granulocytes, 6pLOH, BCOR or BCORL1 somatic mutation, or del(13q) as an isolated somatic abnormality—count towards AA-associated somatic changes score, with only one required for positivity. AA-associated conditions are defined in Supplemental Table S2, and IBMFS red flags are listed in Supplemental Table S1. Lymphocyte telomere length is considered abnormal for measurements <1st percentile compared to age-matched control, as measured by flow-FISH clinical assay. Columns for severity, acuity, age, IBMFS red flag, AA-associated condition, AA somatic, and TL <1st percentile represent the number of points assigned for each individual score component. PASS denotes the total score derived from summing individual points across components and serves as the final classification metric.

		Age at BMF evaluation	Years from first abnormal CBC to BMF diagnosis	Cytopenia Severity	Any of PNH >0.5%, 6pLOH, BCOR, del13q AT DIAGNOSIS	Any of PNH >0.5%, 6pLOH, BCOR or del 13q at ANY POINT	AA associated conditions	IBMF red flags	Lymphocyte telomere length	1. Severity	2. Acuity	3. Age ≥60	4. IBMFS red flag	5. AA associated condition	6. AA somatic	7. TL <1st	PASS
PENN001	AA	65.4	0.27	SAA/VSAA	.	.	.	.	NA	20	10	10	10	0	0	0	50
PENN002	IBMFS	31.7	1.52	NSAA	.	.	.	AVN	Under1	0	-20	-10	-20	0	0	-20	-70
PENN003	AA	40.8	0.04	SAA/VSAA	.	.	.	.	NA	20	10	0	10	0	0	0	40
PENN004	AA	40.0	0.00	SAA/VSAA	.	.	.	.	NA	20	10	0	10	0	0	0	40
PENN005	AA	53.7	0.06	SAA/VSAA	.	6pLOH	.	.	Between1and10	20	10	0	10	0	0	0	40
PENN006	AA	55.7	0.27	SAA/VSAA	.	.	.	.	Under1	20	10	0	10	0	0	-20	20
PENN007	AA	56.4	0.14	SAA/VSAA	.	.	.	.	NA	20	10	0	10	0	0	0	40
PENN008	AA	45.9	0.16	SAA/VSAA	.	.	Hodgkins	.	NA	20	10	0	10	10	0	0	50
PENN009	AA	75.0	0.01	SAA/VSAA	.	.	.	.	NA	20	10	10	10	0	0	0	50
PENN010	AA	73.8	0.92	SAA/VSAA	.	.	.	.	NA	20	10	10	10	0	0	0	50
PENN011	IBMFS	36.5	1.22	NSAA	.	.	.	congenital abnormality	NA	0	-20	-10	-20	0	0	0	-50
PENN012	IBMFS	18.8	2.02	NSAA	.	.	.	cirrhosis	Between1and10	0	-20	-10	-20	0	0	0	-50
PENN013	AA	57.6	0.00	SAA/VSAA	PNH	.	.	.	NA	20	10	0	10	0	20	0	60
PENN014	AA	77.5	0.01	NSAA	.	.	.	.	NA	0	10	10	10	0	0	0	30
PENN015	AA	42.1	0.08	NSAA	PNH	PNH	.	.	Under1	0	10	-10	10	0	20	-20	10
PENN016	AA	85.4	0.44	SAA/VSAA	.	.	.	.	NA	20	10	10	10	0	0	0	50
PENN017	AA	68.4	0.04	SAA/VSAA	PNH, del13q	PNH	.	.	NA	20	10	10	10	0	20	0	70
PENN018	AA	74.2	0.02	SAA/VSAA	.	.	.	.	NA	20	10	10	10	0	0	0	50
PENN019	AA	19.4	0.00	SAA/VSAA	.	.	.	.	Between1and10	20	10	0	10	0	0	0	40
PENN020	AA	85.4	0.24	SAA/VSAA	.	.	.	.	NA	20	10	10	10	0	0	0	50
PENN021	AA	50.1	0.02	SAA/VSAA	.	.	.	.	Between1and10	20	10	0	10	0	0	0	40
PENN022	AA	70.1	0.00	SAA/VSAA	.	.	.	.	NA	20	10	10	10	0	0	0	50
PENN023	AA	24.6	0.14	SAA/VSAA	PNH	PNH	.	.	NA	20	10	0	10	0	20	0	60
PENN024	AA	42.7	0.01	SAA/VSAA	.	.	.	.	Between1and10	20	10	0	10	0	0	0	40
PENN025	IBMFS	53.7	1.33	SAA/VSAA	.	.	.	SCC larynx, chemo sensitivity	NA	20	-10	0	-20	0	0	0	-10
PENN026	AA	30.8	1.67	SAA/VSAA	.	.	.	.	Between1and10	20	-10	0	10	0	0	0	20
PENN027	AA	65.6	0.03	NSAA	PNH	PNH	.	.	NA	0	10	10	10	0	20	0	50
PENN028	AA	68.6	0.05	SAA/VSAA	.	.	.	.	NA	20	10	10	10	0	0	0	50
PENN029	AA	30.0	0.05	NSAA	PNH	PNH	.	.	NA	0	10	-10	10	0	20	0	30
PENN030	IBMFS	29.6	29.29	NSAA	.	.	.	short stature	Under1	0	-20	-10	-20	0	0	-20	-70
PENN031	AA	61.5	0.22	SAA/VSAA	PNH	6pLOH, PNH	.	.	Normal	20	10	10	10	0	20	0	70
PENN032	AA	54.7	0.28	SAA/VSAA	.	.	.	.	Normal	20	10	0	10	0	0	0	40
PENN033	IBMFS	68.3	3.59	NSAA	.	.	.	ILD, cirrhosis	Between1and10	0	-20	10	-20	0	0	0	-30
PENN034	AA	63.0	0.98	NSAA	6pLOH	6pLOH	.	.	NA	0	10	10	10	0	20	0	50
PENN035	AA	35.4	0.15	SAA/VSAA	.	.	.	.	Normal	20	10	0	10	0	0	0	40
PENN036	AA	71.0	0.48	SAA/VSAA	.	.	.	.	NA	20	10	10	10	0	0	0	50
PENN037	IBMFS	30.8	1.30	NSAA	.	.	.	squamous cell, dysmorphology	Normal	0	-20	-10	-20	0	0	0	-50
PENN038	IBMFS	32.8	0.50	SAA/VSAA	.	.	.	vulvar cancer, chemo sensitivity, thumb	NA	20	10	0	-20	0	0	0	10
PENN039	AA	41.0	0.52	NSAA	PNH	PNH	.	.	NA	0	10	-10	10	0	20	0	30
PENN040	AA	27.6	1.49	NSAA	.	PNH	.	.	Normal	0	-20	-10	10	0	0	0	-20
PENN041	AA	67.5	1.30	SAA/VSAA	PNH	PNH	.	.	NA	20	-10	10	10	0	20	0	50
PENN042	AA	34.1	7.07	SAA/VSAA	.	.	.	.	Normal	20	-10	0	10	0	0	0	20

PENN043	AA	46.6	0.01	SAA/VSAA	.	.	.	.	Between1and10	20	10	0	10	0	0	0	40
PENN044	AA	56.4	0.69	SAA/VSAA	.	.	.	.	NA	20	10	0	10	0	0	0	40
PENN045	IBMFS	38.9	0.75	NSAA	.	.	.	father IPF	Under1	0	10	-10	-20	0	0	-20	-40
PENN046	AA	51.6	0.06	SAA/VSAA	.	.	.	.	Normal	20	10	0	10	0	0	0	40
PENN047	AA	63.4	6.98	NSAA	.	.	.	sister leukemia	Between1and10	0	-20	10	-20	0	0	0	-30
PENN048	AA	55.4	0.00	SAA/VSAA	.	.	.	.	Normal	20	10	0	10	0	0	0	40
PENN049	AA	72.6	0.00	SAA/VSAA	6pLOH	6pLOH	.	.	Normal	20	10	10	10	0	20	0	70
PENN050	AA	61.0	0.12	SAA/VSAA	.	BCOR	.	.	Normal	20	10	10	10	0	0	0	50
PENN051	IBMFS	44.8	16.01	NSAA	.	.	.	ILD, AVN, mother with leukemia	Under1	0	-20	-10	-20	0	0	-20	-70
PENN052	AA	20.6	4.14	SAA/VSAA	PNH	PNH, BCOR	.	.	Normal	20	-10	0	10	0	20	0	40
PENN053	AA	56.5	0.00	NSAA	.	.	.	.	Normal	0	10	-10	10	0	0	0	10
PENN054	IBMFS	24.5	23.72	NSAA	.	.	.	congenital abnormality	Normal	0	-20	-10	-20	0	0	0	-50
PENN055	AA	66.7	0.75	SAA/VSAA	.	.	.	.	Normal	20	10	10	10	0	0	0	50
PENN056	AA	26.7	0.04	SAA/VSAA	PNH	PNH	.	.	Between1and10	20	10	0	10	0	20	0	60
PENN057	AA	23.6	0.01	SAA/VSAA	.	.	.	.	Normal	20	10	0	10	0	0	0	40
PENN058	IBMFS	38.2	0.00	NSAA	.	.	.	father with BMF	Between1and10	0	10	-10	-20	0	0	0	-20
PENN059	IBMFS	62.4	10.99	SAA/VSAA	.	.	.	chemotherapy sensitivity	Under1	20	-10	10	-20	0	0	-20	-20
PENN060	AA	57.1	0.02	SAA/VSAA	.	.	.	.	Normal	20	10	0	10	0	0	0	40
PENN061	AA	33.4	0.00	SAA/VSAA	PNH, BCOR	PNH, BCOR	.	.	NA	20	10	0	10	0	20	0	60
PENN062	AA	58.0	2.97	NSAA	.	.	.	.	NA	0	-20	-10	10	0	0	0	-20
PENN063	AA	30.1	0.98	SAA/VSAA	PNH	PNH	.	father with cytopenias	Normal	20	10	0	-20	0	20	0	30
PENN064	AA	33.2	1.86	SAA/VSAA	.	PNH	.	.	Normal	20	-10	0	10	0	0	0	20
PENN065	AA	76.5	0.21	SAA/VSAA	PNH	PNH	.	.	NA	20	10	10	10	0	20	0	70
PENN066	AA	56.1	0.12	SAA/VSAA	.	.	.	.	NA	20	10	0	10	0	0	0	40
PENN067	IBMFS	22.2	20.90	NSAA	.	.	.	congenital abnormality	Normal	0	-20	-10	-20	0	0	0	-50
PENN068	AA	75.1	0.03	SAA/VSAA	PNH	PNH	.	.	Normal	20	10	10	10	0	20	0	70
PENN069	AA	77.0	0.23	SAA/VSAA	.	.	Use of checkpoint inhibitors, osimertinib	.	NA	20	10	10	10	10	0	0	60
PENN070	AA	82.1	0.01	NSAA	.	.	.	.	NA	0	10	10	10	0	0	0	30
PENN071	AA	69.1	0.04	SAA/VSAA	.	.	.	.	NA	20	10	10	10	0	0	0	50
PENN072	AA	75.7	0.04	SAA/VSAA	.	6pLOH	.	.	NA	20	10	10	10	0	0	0	50
PENN073	IBMFS	51.5	1.31	NSAA	.	.	.	ILD	Under1	0	-20	-10	-20	0	0	-20	-70
PENN074	AA	67.8	0.12	SAA/VSAA	.	.	.	.	NA	20	10	10	10	0	0	0	50
PENN075	AA	47.0	0.04	SAA/VSAA	.	.	.	.	NA	20	10	0	10	0	0	0	40
PENN076	AA	58.6	0.66	SAA/VSAA	.	PNH	.	.	Normal	20	10	0	10	0	0	0	40
PENN077	AA	24.9	0.01	SAA/VSAA	.	BCOR	.	neurologic abnormalities, autism-spectrum	Normal	20	10	0	-20	0	0	0	10
PENN078	AA	20.3	0.00	SAA/VSAA	PNH	PNH	.	.	Between1and10	20	10	0	10	0	20	0	60
PENN079	AA	25.8	0.04	SAA/VSAA	.	.	.	asymmetric thumbs	Normal	20	10	0	-20	0	0	0	10
PENN080	AA	34.3	0.00	SAA/VSAA	.	.	.	.	Normal	20	10	0	10	0	0	0	40
PENN081	IBMFS	32.3	13.73	NSAA	.	.	.	AVN	Under1	0	-20	-10	-20	0	0	-20	-70
PENN082	AA	42.1	0.30	NSAA	PNH	PNH	.	.	Normal	0	10	-10	10	0	20	0	30
PENN083	AA	69.0	0.03	SAA/VSAA	PNH	PNH	.	.	NA	20	10	10	10	0	20	0	70
PENN084	AA	67.8	0.02	NSAA	.	.	.	.	NA	0	10	10	10	0	0	0	30
PENN085	AA	66.7	0.01	SAA/VSAA	.	.	.	.	NA	20	10	10	10	0	0	0	50
PENN086	AA	81.7	0.01	NSAA	PNH	PNH	.	.	NA	0	10	10	10	0	20	0	50

PENN087	AA	50.3	0.08	SAA/VSAA	.	del 13q	.	.	NA	20	10	0	10	0	0	0	40
PENN088	AA	66.1	0.16	SAA/VSAA	.	del 13q	.	.	NA	20	10	10	10	0	0	0	50
PENN089	AA	27.1	0.11	SAA/VSAA	.	.	hepatitis	.	Normal	20	10	0	10	10	0	0	50
PENN090	AA	19.3	0.01	SAA/VSAA	.	.	.	.	Normal	20	10	0	10	0	0	0	40
PENN091	AA	26.2	0.04	SAA/VSAA	PNH, 6pLOH	6pLOH	.	.	Normal	20	10	0	10	0	20	0	60
PENN092	AA	54.7	0.38	NSAA	.	.	.	.	Normal	0	10	-10	10	0	0	0	10
PENN093	IBMFS	70.1	2.54	NSAA	.	.	.	ILD	Between1and10	0	-20	10	-20	0	0	0	-30
PENN094	AA	59.7	0.51	NSAA	.	.	.	.	Normal	0	10	-10	10	0	0	0	10
PENN095	IBMFS	72.4	1.35	NSAA	.	.	.	ILD, rectal cancer, tongue cancer	Under1	0	-20	10	-20	0	0	-20	-50
PENN096	AA	26.8	0.12	SAA/VSAA	.	.	.	.	Normal	20	10	0	10	0	0	0	40
PENN097	AA	49.4	0.21	SAA/VSAA	.	PNH	.	.	NA	20	10	0	10	0	0	0	40
PENN098	AA	86.7	0.14	SAA/VSAA	.	.	.	.	NA	20	10	10	10	0	0	0	50
PENN099	AA	51.9	0.11	SAA/VSAA	PNH	PNH	.	.	NA	20	10	0	10	0	20	0	60
PENN100	IBMFS	33.5	33.55	SAA/VSAA	6pLOH	6pLOH	.	congenital abnormality	Normal	20	-10	0	-20	0	20	0	10
PENN101	AA	76.4	0.12	SAA/VSAA	.	.	.	.	NA	20	10	10	10	0	0	0	50
PENN102	AA	58.1	0.01	SAA/VSAA	.	.	.	.	Between1and10	20	10	0	10	0	0	0	40
PENN103	AA	64.9	0.36	SAA/VSAA	.	BCOR	.	.	Normal	20	10	10	10	0	0	0	50
PENN104	AA	61.2	0.05	SAA/VSAA	.	.	.	.	NA	20	10	10	10	0	0	0	50
PENN105	IBMFS	69.8	0.65	NSAA	.	.	.	ILD	Between1and10	0	10	10	-20	0	0	0	0
PENN106	AA	22.4	0.36	SAA/VSAA	PNH	PNH	.	.	NA	20	10	0	10	0	20	0	60
PENN107	AA	29.0	11.65	NSAA	.	.	.	.	Between1and10	0	-20	-10	10	0	0	0	-20
PENN108	AA	44.3	0.44	SAA/VSAA	.	.	.	dysmorphology	Normal	20	10	0	-20	0	0	0	10
PENN109	AA	48.8	4.90	SAA/VSAA	PNH	PNH	.	.	Normal	20	-10	0	10	0	20	0	40
PENN110	IBMFS	35.7	0.87	NSAA	.	.	.	cirrhosis	Under1	0	10	-10	-20	0	0	-20	-40
PENN111	AA	21.1	0.35	NSAA	.	.	.	.	Normal	0	10	-10	10	0	0	0	10
PENN112	AA	19.4	0.09	SAA/VSAA	PNH	PNH	.	.	Normal	20	10	0	10	0	20	0	60
PENN113	AA	67.1	0.00	SAA/VSAA	PNH	PNH	.	.	NA	20	10	10	10	0	20	0	70
PENN114	AA	61.0	0.03	NSAA	.	BCOR	thymoma	.	NA	0	10	10	10	10	0	0	40
PENN115	AA	50.6	0.00	SAA/VSAA	.	PNH, BCOR	.	.	Normal	20	10	0	10	0	0	0	40
PENN116	AA	52.7	0.59	NSAA	PNH	PNH, BCOR	.	.	Normal	0	10	-10	10	0	20	0	30
PENN117	AA	32.0	0.01	SAA/VSAA	.	.	.	.	Normal	20	10	0	10	0	0	0	40
PENN118	AA	56.6	0.61	SAA/VSAA	PNH	PNH	.	.	NA	20	10	0	10	0	20	0	60
PENN119	AA	48.8	0.01	SAA/VSAA	.	.	Hodgkins	.	NA	20	10	0	10	10	0	0	50
PENN120	AA	76.1	0.00	SAA/VSAA	.	.	.	.	NA	20	10	10	10	0	0	0	50
PENN121	AA	41.1	0.05	SAA/VSAA	.	PNH	.	.	Normal	20	10	0	10	0	0	0	40
PENN122	AA	65.8	0.67	NSAA	.	PNH	.	.	NA	0	10	10	10	0	0	0	30
PENN123	AA	22.9	0.01	SAA/VSAA	.	.	.	.	Normal	20	10	0	10	0	0	0	40
PENN124	AA	69.6	0.08	SAA/VSAA	.	.	.	.	NA	20	10	10	10	0	0	0	50
PENN125	IBMFS	22.5	4.01	NSAA	.	.	.	IPF, cirrhosis, congenital defect	Under1	0	-20	-10	-20	0	0	-20	-70
PENN126	AA	76.7	0.18	SAA/VSAA	PNH	PNH	.	.	NA	20	10	10	10	0	20	0	70
PENN127	AA	73.7	0.18	SAA/VSAA	PNH	PNH	.	.	NA	20	10	10	10	0	20	0	70
PENN128	AA	55.8	0.81	SAA/VSAA	PNH, 6pLOH	6pLOH, BCOR, del 13q	.	.	NA	20	10	0	10	0	20	0	60
PENN129	IBMFS	34.9	21.09	NSAA	.	.	.	brother with BMF	Under1	0	-20	-10	-20	0	0	-20	-70
PENN130	AA	68.8	1.54	SAA/VSAA	.	.	.	.	NA	20	-10	10	10	0	0	0	30
PENN131	AA	75.6	0.08	SAA/VSAA	.	.	.	.	NA	20	10	10	10	0	0	0	50
PENN132	AA	61.8	1.56	SAA/VSAA	PNH	PNH	.	.	NA	20	-10	10	10	0	20	0	50
PENN133	AA	60.8	0.01	SAA/VSAA	.	PNH	.	.	NA	20	10	10	10	0	0	0	50

PENN134	AA	72.0	0.32	NSAA	PNH	PNH	.	.	NA	0	10	10	10	0	20	0	50
PENN135	AA	52.8	0.32	NSAA	PNH	PNH	.	.	NA	0	10	-10	10	0	20	0	30
PENN136	AA	57.1	0.00	NSAA	.	.	.	.	NA	0	10	-10	10	0	0	0	10
PENN137	AA	22.7	0.01	SAA/VSAA	.	BCOR	.	.	Normal	20	10	0	10	0	0	0	40
PENN138	AA	61.0	0.00	SAA/VSAA	.	.	.	.	NA	20	10	10	10	0	0	0	50
PENN139	AA	79.5	0.89	SAA/VSAA	.	.	.	.	NA	20	10	10	10	0	0	0	50
PENN140	AA	62.2	0.11	NSAA	.	BCOR	.	.	Normal	0	10	10	10	0	0	0	30
PENN141	AA	42.2	5.08	NSAA	.	.	Previously tolerated chemotherapy or radiation	.	NA	0	-20	-10	10	10	0	0	-10
PENN142	AA	26.6	0.29	NSAA	PNH	PNH	.	.	Between1and10	0	10	-10	10	0	20	0	30
PENN143	AA	30.0	0.04	SAA/VSAA	BCOR	BCOR	.	.	Between1and10	20	10	0	10	0	20	0	60
PENN144	IBMFS	33.3	9.04	NSAA	.	.	.	AVN, father with IPF	Under1	0	-20	-10	-20	0	0	-20	-70
PENN145	IBMFS	60.4	1.10	NSAA	.	.	.	IPF, family history of BMF and IPF	Under1	0	-20	10	-20	0	0	-20	-50
PENN146	AA	49.3	0.75	SAA/VSAA	PNH, del13q	PNH, del 13q	.	.	Normal	20	10	0	10	0	20	0	60
PENN147	IBMFS	57.4	2.82	NSAA	.	.	.	IPF, gray hair, mother leukemia	Under1	0	-20	-10	-20	0	0	-20	-70
PENN148	AA	64.7	0.02	SAA/VSAA	.	.	.	.	NA	20	10	10	10	0	0	0	50
PENN149	AA	56.0	0.07	SAA/VSAA	.	.	.	.	Normal	20	10	0	10	0	0	0	40
PENN150	AA	33.5	0.12	SAA/VSAA	PNH	PNH	.	.	Normal	20	10	0	10	0	20	0	60
PENN151	AA	61.1	0.00	SAA/VSAA	PNH	PNH	.	.	NA	20	10	10	10	0	20	0	70
PENN152	AA	48.1	0.06	SAA/VSAA	.	.	.	.	NA	20	10	0	10	0	0	0	40
PENN153	IBMFS	62.0	9.01	NSAA	.	.	.	ILD, fam hx ILD	Under1	0	-20	10	-20	0	0	-20	-50
PENN154	AA	25.3	0.01	SAA/VSAA	.	PNH	.	.	NA	20	10	0	10	0	0	0	40
PENN155	AA	63.4	0.67	SAA/VSAA	PNH, 6pLOH	6pLOH	.	.	NA	20	10	10	10	0	20	0	70
PENN156	AA	44.8	0.08	SAA/VSAA	.	PNH, del 13q, BCORL1	.	.	Normal	20	10	0	10	0	0	0	40
PENN157	AA	61.2	0.09	SAA/VSAA	PNH	BCORL1	.	.	Between1and10	20	10	10	10	0	20	0	70
PENN158	AA	47.8	0.05	SAA/VSAA	.	PNH	.	.	NA	20	10	0	10	0	0	0	40
PENN159	AA	38.6	0.00	SAA/VSAA	PNH	PNH	.	.	Normal	20	10	0	10	0	20	0	60
PENN160	AA	63.2	0.02	SAA/VSAA	.	.	.	.	NA	20	10	10	10	0	0	0	50
PENN161	AA	22.1	0.09	SAA/VSAA	.	PNH, BCOR	.	.	NA	20	10	0	10	0	0	0	40
PENN162	IBMFS	52.2	7.84	NSAA	.	.	.	ILD	Under1	0	-20	-10	-20	0	0	-20	-70
PENN163	AA	23.8	0.00	SAA/VSAA	PNH	PNH	.	.	Normal	20	10	0	10	0	20	0	60
PENN164	AA	50.8	0.00	NSAA	.	BCOR	.	.	Normal	0	10	-10	10	0	0	0	10
PENN165	AA	54.0	0.16	SAA/VSAA	.	.	Previously tolerated chemotherapy or radiation	.	NA	20	10	0	10	10	0	0	50
PENN166	IBMFS	60.5	8.52	NSAA	.	.	.	ILD, cirrhosis	Between1and10	0	-20	10	-20	0	0	0	-30
PENN167	AA	20.5	3.01	SAA/VSAA	.	PNH	.	.	Between1and10	20	-10	0	10	0	0	0	20
PENN168	AA	66.8	0.04	SAA/VSAA	PNH	.	.	.	NA	20	10	10	10	0	20	0	70
PENN169	AA	28.5	0.02	SAA/VSAA	PNH	PNH	.	.	Normal	20	10	0	10	0	20	0	60
PENN170	AA	50.8	1.93	SAA/VSAA	.	.	.	tongue cancer, CVID, mother glioblastoma	Between1and10	20	-10	0	-20	0	0	0	-10
PENN171	AA	51.3	0.01	SAA/VSAA	.	.	.	.	NA	20	10	0	10	0	0	0	40
PENN172	AA	58.0	0.04	SAA/VSAA	PNH	PNH	.	.	NA	20	10	0	10	0	20	0	60
PENN173	AA	63.7	0.08	SAA/VSAA	.	PNH	.	.	NA	20	10	10	10	0	0	0	50
PENN174	IBMFS	20.6	1.74	NSAA	.	.	.	.	Normal	0	-20	-10	10	0	0	0	-20

PENN175	AA	75.3	0.34	SAA/VSAA	.	.	.	.	NA	20	10	10	10	0	0	0	50
PENN176	AA	62.5	0.53	SAA/VSAA	PNH	PNH, BCOR, del 13q	.	.	NA	20	10	10	10	0	20	0	70
PENN177	AA	27.4	0.10	SAA/VSAA	PNH, BCOR	PNH	hepatitis	.	Normal	20	10	0	10	10	20	0	70
PENN178	AA	52.6	1.13	NSAA	PNH	PNH, BCOR	.	.	Normal	0	-20	-10	10	0	20	0	0
PENN179	AA	22.5	0.42	SAA/VSAA	.	PNH	.	.	Between1and10	20	10	0	10	0	0	0	40
PENN180	AA	65.3	0.05	SAA/VSAA	PNH	PNH	.	.	NA	20	10	10	10	0	20	0	70
PENN181	AA	51.6	0.23	NSAA	.	.	.	.	NA	0	10	-10	10	0	0	0	10
PENN182	AA	46.9	0.00	NSAA	6pLOH	6pLOH	.	.	Normal	0	10	-10	10	0	20	0	30
PENN183	AA	53.4	0.00	SAA/VSAA	PNH	PNH	.	.	Normal	20	10	0	10	0	20	0	60
PENN184	AA	78.7	0.12	SAA/VSAA	del13q	del 13q	.	.	NA	20	10	10	10	0	20	0	70
PENN185	AA	28.4	2.01	SAA/VSAA	.	.	.	.	Between1and10	20	-10	0	10	0	0	0	20
PENN186	AA	41.7	3.21	NSAA	6pLOH	6pLOH	eosinophilic fasciitis	.	Between1and10	0	-20	-10	10	10	20	0	10
PENN187	AA	45.9	0.05	SAA/VSAA	PNH	PNH	.	.	Between1and10	20	10	0	10	0	20	0	60
PENN188	AA	42.1	0.70	SAA/VSAA	BCOR	BCOR	.	.	Normal	20	10	0	10	0	20	0	60
PENN189	AA	25.0	0.16	SAA/VSAA	PNH	PNH	.	.	NA	20	10	0	10	0	20	0	60
PENN190	AA	21.5	1.04	NSAA	.	.	.	.	Normal	0	-20	-10	10	0	0	0	-20
PENN191	AA	64.9	0.30	SAA/VSAA	PNH	PNH	.	.	NA	20	10	10	10	0	20	0	70
PENN192	AA	70.3	0.10	SAA/VSAA	PNH	PNH	Previously tolerated chemotherap y or radiation	.	Normal	20	10	10	10	10	20	0	80
PENN194	IBMFS	55.2	15.00	NSAA	.	.	.	IPF, AVN, family history	Under1	0	-20	-10	-20	0	0	-20	-70
PENN195	IBMFS	77.3	6.10	NSAA	.	.	.	IPF	Between1and10	0	-20	10	-20	0	0	0	-30
PENN196	IBMFS	65.0	0.80	NSAA	.	.	.	IPF	Under1	0	10	10	-20	0	0	-20	-20
PENN197	IBMFS	28.2	0.90	NSAA	.	.	.	.	Between1and10	0	10	-10	10	0	0	0	10
PENN198	IBMFS	67.8	lifelong	NSAA	.	.	.	Cirrhosis, IPF, 2 brothers with MDS or AML	Between1and10	0	-20	10	-20	0	0	0	-30
PENN199	IBMFS	34.5	16.00	NSAA	.	.	.	multiple bacterial infections- lymphedema - severe HPV - severe and persistent HSV FH son motor malformation	Normal	0	-20	-10	-20	0	0	0	-50
PENN200	IBMFS	30.4	lifelong	NSAA	.	.	.	small thumbs VSD; father with triphalangeal thumb	NA	0	-20	-10	-20	0	0	0	-50
PENN201	IBMFS	38.7	lifelong	NSAA	.	.	.	small for age - thumb malformation café au lait spots and frequent infections	NA	0	-20	-10	-20	0	0	0	-50
PENN202	IBMFS	33.8	lifelong	NSAA	.	.	.	IPF, Brother with short telomere	Under1	0	-20	-10	-20	0	0	-20	-70
PENN203	IBMFS	24.4	lifelong	NSAA	.	.	.	.	Normal	0	-20	-10	10	0	0	0	-20
PENN204	IBMFS	27.5	0.00	NSAA	.	.	.	FH Wilms tumor, T-cell ALL, medulloblastoma, and AML in sister; personal history of colon cancer, BCC, and metastatic neuroendocrine cancer, and sensitivity to chemotherapy	NA	0	10	-10	-20	0	0	0	-20
PENN205	IBMFS	31.0	1.62	NSAA	.	.	.	.	Under1	0	-20	-10	10	0	0	-20	-40
PENN206	IBMFS	37.7	2.16	NSAA	.	.	.	brother with FA; patient had café au lait, hypoplastic thumb, dysplasia upper palate, SCC oropharynx	NA	0	-20	-10	-20	0	0	0	-50

PENN207	IBMFS	22.5	4.41	NSAA	.	.	.	skeletal dysplasia, short stature	NA	0	-20	-10	-20	0	0	0	-50
PENN208	IBMFS	57.8	25.96	NSAA	.	.	.	Family hx - brother had leukemia; short stature	Under1	0	-20	-10	-20	0	0	-20	-70
PENN209	IBMFS	58.5	15.51	NSAA	.	.	.	brother with chronic unexplained thrombocytopenia	Between1and10	0	-20	-10	-20	0	0	0	-50
PENN210	IBMFS	18.2	18.00	NSAA	.	.	.	VSD, ASD, and PDA- closed spontaneously. Neurodevelopmental delays, seizures; femoral retroversion; amblyopia, growth hormone deficiency.	Under1	0	-20	-10	-20	0	0	-20	-70
PENN211	IBMFS	28.6	14.30	NSAA	.	.	.	recurrent warts	NA	0	-20	-10	-20	0	0	0	-50
PENN212	IBMFS	26.4	17.66	NSAA	.	.	.	dextrocardia	NA	0	-20	-10	-20	0	0	0	-50
PENN213	IBMFS	25.4	23.98	NSAA	.	.	.	.	NA	0	-20	-10	10	0	0	0	-20

Supplemental Dataset S3. Individual-level data and scoring for the NIH/USP cohort

This table shows individual-level data from the NIH/USP cohort. The original cohort was previously published by Gutierrez-Rodrigues et al. *(Gutierrez-Rodrigues F, Munger E, Ma X, et al. Differential diagnosis of bone marrow failure syndromes guided by machine learning. Blood. 2023;141(17):2100–2113)*.

For our study, patients were selected from the published dataset using the structure described in Figure S1 to ensure patients analyzed match our study inclusion criteria, as detailed in the methods. Age refers to the patient's age at the time of bone marrow failure evaluation. Longstanding cytopenias or macrocytosis was used to distinguish chronic versus acute disease, with "Yes" corresponding to chronic (>1 year) and "No" to acute (<1 year). Cytopenia severity is defined according to standard criteria. PNH clone size was recorded to a minimum detection threshold of 1%, with ≥1% considered positive. Data for other AA-associated somatic changes aside from PNH (6pLOH, del(13)(q), BCOR/BCORL1) were not available and were assigned 0 points. In this dataset, we defined IBMFS red flags by the presence of dyskeratosis congenita mucocutaneous triad, family history in immediate relatives, and physical anomalies. No data were available on AA-associated conditions, which were assigned 0 points. Lymphocyte telomere length (TL) was considered abnormal for <1st percentile of age-matched controls. Columns for severity, acuity, age, IBMFS red flag, AA somatic, and TL <1st percentile represent the number of points assigned for each feature. PASS indicates the final sum of points used for classification.

StudyID	Diagnosis	Clnical and laboratory data at diagnosis								Individual Score Components							PASS Score
		Age	Longstanding cytopenias or macrocytosis	Severity	PNH clone	DC clinical triad	Physical anomalies	Immediate family members with similar phenotype	Lymphocyte TL	1. Severity	2. Acuity	3. Age ≥60	4. IBMFS red flag	5. AA associated condition	6. AA somatic	7. TL <1st	
NIH216	TBD	<60	Yes	NSAA	none	No	No	No	Under1	0	-20	-10	10	0	0	-20	-40
330-1	SDS	<60	No	NSAA	none	No	Yes	No	No	0	10	-10	-20	0	0	0	-20
353-1	TBD	<60	Yes	NSAA	none	No	No	No	Under1	0	-20	-10	10	0	0	-20	-40
NIH318	TBD	<60	No	NSAA	none	No	No	No	Under1	0	10	-10	10	0	0	-20	-10
163-1	AA	<60	No	NSAA	na	No	Yes	No	No	0	10	-10	-20	0	0	0	-20
4-1	AA	<60	No	NSAA	none	No	No	No	Under1	0	10	-10	10	0	0	-20	-10
NIH223	AA	<60	No	SAA/VSAA	none	No	No	No	No	20	10	0	10	0	0	0	40
NIH200	AA	<60	Yes	SAA/VSAA	none	No	No	No	No	20	-10	0	10	0	0	0	20
NIH022	AA	60+	No	NSAA	none	No	No	No	No	0	10	10	10	0	0	0	30
NIH033	AA	60+	No	NSAA	none	No	No	Yes	No	0	10	10	-20	0	0	0	0
NIH167	AA	<60	No	NSAA	none	No	No	Yes	No	0	10	-10	-20	0	0	0	-20
NIH065	AA	<60	No	SAA/VSAA	4.8	No	No	No	No	20	10	0	10	0	20	0	60
NIH004	TBD	<60	No	NSAA	none	No	No	Yes	Under1	0	10	-10	-20	0	0	-20	-40
NIH308	TBD	<60	No	NSAA	none	Yes	No	No	Under1	0	10	-10	-20	0	0	-20	-40
NIH007	TBD	<60	Yes	NSAA	none	No	No	No	Under1	0	-20	-10	10	0	0	-20	-40
NIH020	AA	<60	No	NSAA	none	No	No	No	Under1	0	10	-10	10	0	0	-20	-10
NIH305	TBD	<60	No	NSAA	none	No	No	Yes	Under1	0	10	-10	-20	0	0	-20	-40
NIH310	TBD	<60	Yes	NSAA	none	No	No	No	Under1	0	-20	-10	10	0	0	-20	-40
NIH239	AA	<60	No	SAA/VSAA	none	No	No	No	No	20	10	0	10	0	0	0	40
NIH037	AA	<60	Yes	NSAA	none	No	No	Yes	Under1	0	-20	-10	-20	0	0	-20	-70
NIH008	TBD	<60	Yes	NSAA	none	No	No	Yes	Under1	0	-20	-10	-20	0	0	-20	-70
NIH040	AA	<60	No	NSAA	none	No	No	No	No	0	10	-10	10	0	0	0	10
NIH044	AA	60+	No	SAA/VSAA	none	No	No	No	No	20	10	10	10	0	0	0	50
NIH129	AA	60+	No	SAA/VSAA	none	No	No	No	No	20	10	10	10	0	0	0	50
NIH206	AA	<60	No	SAA/VSAA	none	No	No	No	No	20	10	0	10	0	0	0	40
NIH005	TBD	<60	No	NSAA	none	No	No	Yes	Under1	0	10	-10	-20	0	0	-20	-40
NIH023	AA	<60	No	NSAA	1.3	No	No	Yes	No	0	10	-10	-20	0	20	0	0
NIH297	AA	<60	No	NSAA	na	No	No	No	No	0	10	-10	10	0	0	0	10
NIH058	AA	<60	No	SAA/VSAA	9.5	No	No	No	No	20	10	0	10	0	20	0	60
NIH021	AA	<60	No	NSAA	none	No	No	No	Under1	0	10	-10	10	0	0	-20	-10
NIH225	AA	<60	No	NSAA	5.8	No	No	No	No	0	10	-10	10	0	20	0	30
NIH026	AA	<60	No	NSAA	none	No	No	No	Under1	0	10	-10	10	0	0	-20	-10
NIH073	AA	60+	No	SAA/VSAA	none	No	No	No	No	20	10	10	10	0	0	0	50
NIH011	AA	<60	No	NSAA	none	No	No	No	Under1	0	10	-10	10	0	0	-20	-10
NIH028	AA	<60	No	NSAA	none	No	No	No	No	0	10	-10	10	0	0	0	10
NIH118	AA	<60	No	SAA/VSAA	none	No	No	No	No	20	10	0	10	0	0	0	40
NIH210	AA	<60	No	SAA/VSAA	na	No	No	No	No	20	10	0	10	0	0	0	40
NIH288	AA	60+	No	NSAA	33.6	No	No	No	No	0	10	10	10	0	20	0	50

288-1	TBD	<60	No	NSAA	none	Yes	No	No	Under1	0	10	-10	-20	0	0	-20	-40
NIH006	TBD	<60	No	NSAA	none	No	No	No	Under1	0	10	-10	10	0	0	-20	-10
NIH029	AA	<60	No	NSAA	2.8	No	No	Yes	No	0	10	-10	-20	0	20	0	0
NIH025	AA	<60	No	NSAA	none	No	No	No	No	0	10	-10	10	0	0	0	10
NIH140	AA	<60	Yes	NSAA	none	No	Yes	No	Under1	0	-20	-10	-20	0	0	-20	-70
NIH160	AA	<60	No	NSAA	none	No	No	No	No	0	10	-10	10	0	0	0	10
NIH197	AA	<60	No	SAA/VSAA	none	No	No	No	No	20	10	0	10	0	0	0	40
NIH220	TBD	<60	Yes	NSAA	none	No	No	No	Under1	0	-20	-10	10	0	0	-20	-40
161-1	AA	<60	No	NSAA	na	No	Yes	No	Under1	0	10	-10	-20	0	0	-20	-40
NIH295	AA	<60	No	SAA/VSAA	na	No	No	No	No	20	10	0	10	0	0	0	40
NIH262	AA	60+	No	NSAA	none	No	No	No	No	0	10	10	10	0	0	0	30
NIH282	AA	<60	No	SAA/VSAA	none	No	No	No	No	20	10	0	10	0	0	0	40
NIH213	TBD	<60	Yes	SAA/VSAA	none	No	No	No	Under1	20	-10	0	10	0	0	-20	0
NIH136	AA	<60	No	SAA/VSAA	32.3	No	No	No	No	20	10	0	10	0	20	0	60
NIH180	AA	60+	No	SAA/VSAA	6.9	No	No	No	No	20	10	10	10	0	20	0	70
NIH031	AA	<60	Yes	NSAA	none	No	No	No	No	0	-20	-10	10	0	0	0	-20
NIH071	AA	<60	No	NSAA	2.1	No	No	No	No	0	10	-10	10	0	20	0	30
167-1	TBD	<60	Yes	NSAA	na	Yes	No	No	Under1	0	-20	-10	-20	0	0	-20	-70
291-1	TBD	<60	No	NSAA	none	Yes	Yes	No	Under1	0	10	-10	-20	0	0	-20	-40
NIH116	AA	<60	No	NSAA	97.8	No	No	No	No	0	10	-10	10	0	20	0	30
NIH036	AA	<60	No	NSAA	none	No	No	No	No	0	10	-10	10	0	0	0	10
382-1	TBD	<60	Yes	NSAA	none	Yes	No	Yes	Under1	0	-20	-10	-20	0	0	-20	-70
NIH045	AA	<60	No	NSAA	none	No	No	No	No	0	10	-10	10	0	0	0	10
NIH179	AA	<60	No	NSAA	4	No	No	No	No	0	10	-10	10	0	20	0	30
NIH053	AA	<60	No	NSAA	none	No	No	No	Under1	0	10	-10	10	0	0	-20	-10
NIH185	AA	<60	No	SAA/VSAA	none	No	No	No	No	20	10	0	10	0	0	0	40
NIH013	FA	<60	No	NSAA	none	No	Yes	No	Under1	0	10	-10	-20	0	0	-20	-40
NIH009	TBD	<60	Yes	NSAA	none	No	No	No	Under1	0	-20	-10	10	0	0	-20	-40
NIH312	TBD	<60	Yes	SAA/VSAA	none	No	No	Yes	Under1	20	-10	0	-20	0	0	-20	-30
NIH174	AA	<60	No	NSAA	1.4	No	Yes	No	No	0	10	-10	-20	0	20	0	0
NIH102	AA	<60	No	SAA/VSAA	8.3	No	No	No	No	20	10	0	10	0	20	0	60
NIH266	AA	<60	No	SAA/VSAA	none	No	No	No	No	20	10	0	10	0	0	0	40
NIH014	TBD	<60	Yes	NSAA	none	No	Yes	Yes	No	0	-20	-10	-20	0	0	0	-50
NIH165	AA	<60	No	NSAA	none	No	No	No	No	0	10	-10	10	0	0	0	10
NIH195	AA	60+	No	NSAA	none	No	No	No	No	0	10	10	10	0	0	0	30
NIH172	AA	60+	No	NSAA	3.3	No	No	No	No	0	10	10	10	0	20	0	50
NIH229	AA	60+	No	NSAA	none	No	No	No	No	0	10	10	10	0	0	0	30
96-1	TBD	<60	No	NSAA	none	Yes	No	Yes	Under1	0	10	-10	-20	0	0	-20	-40
NIH088	AA	<60	No	NSAA	none	No	No	No	No	0	10	-10	10	0	0	0	10
NIH063	AA	<60	No	SAA/VSAA	none	No	No	No	No	20	10	0	10	0	0	0	40
NIH232	AA	60+	No	SAA/VSAA	none	No	No	No	No	20	10	10	10	0	0	0	50
NIH041	AA	<60	No	NSAA	none	No	No	No	No	0	10	-10	10	0	0	0	10
NIH248	AA	<60	No	SAA/VSAA	none	No	No	No	No	20	10	0	10	0	0	0	40

NIH306	TBD	<60	No	NSAA	none	No	No	No	Under1	0	10	-10	10	0	0	-20	-10
NIH176	TBD	<60	Yes	NSAA	none	No	No	Yes	Under1	0	-20	-10	-20	0	0	-20	-70
NIH279	AA	<60	No	SAA/VSAA	none	No	No	No	No	20	10	0	10	0	0	0	40
NIH284	AA	60+	No	SAA/VSAA	10.5	No	No	No	No	20	10	10	10	0	20	0	70
NIH002	TBD	<60	Yes	NSAA	none	No	No	No	Under1	0	-20	-10	10	0	0	-20	-40
NIH219	TBD	<60	Yes	NSAA	none	No	No	No	Under1	0	-20	-10	10	0	0	-20	-40
NIH059	AA	<60	No	SAA/VSAA	1.5	No	No	No	No	20	10	0	10	0	20	0	60
NIH313	TBD	<60	Yes	NSAA	none	No	No	No	Under1	0	-20	-10	10	0	0	-20	-40
NIH327	TBD	<60	Yes	NSAA	none	No	No	No	Under1	0	-20	-10	10	0	0	-20	-40
NIH012	FA	<60	No	NSAA	none	No	No	No	No	0	10	-10	10	0	0	0	10
NIH141	AA	<60	No	NSAA	none	No	No	No	No	0	10	-10	10	0	0	0	10
NIH070	AA	60+	No	SAA/VSAA	none	No	No	No	No	20	10	10	10	0	0	0	50
NIH083	AA	<60	No	NSAA	none	No	No	No	No	0	10	-10	10	0	0	0	10
NIH085	AA	<60	No	NSAA	1.6	No	No	No	No	0	10	-10	10	0	20	0	30
NIH128	AA	<60	No	SAA/VSAA	none	No	No	No	No	20	10	0	10	0	0	0	40
NIH042	AA	<60	No	SAA/VSAA	none	No	No	No	No	20	10	0	10	0	0	0	40
NIH091	AA	60+	No	SAA/VSAA	2.4	No	No	No	No	20	10	10	10	0	20	0	70
NIH177	AA	<60	No	SAA/VSAA	none	No	Yes	No	No	20	10	0	-20	0	0	0	10
NIH230	AA	<60	No	SAA/VSAA	37.7	No	No	No	No	20	10	0	10	0	20	0	60
NIH293	AA	<60	No	SAA/VSAA	none	No	No	No	No	20	10	0	10	0	0	0	40
NIH190	AA	<60	No	SAA/VSAA	none	No	No	No	No	20	10	0	10	0	0	0	40
NIH272	AA	<60	No	SAA/VSAA	none	No	No	No	No	20	10	0	10	0	0	0	40
NIH015	AA	<60	No	NSAA	none	No	No	No	No	0	10	-10	10	0	0	0	10
NIH046	AA	<60	No	NSAA	5.7	No	No	No	No	0	10	-10	10	0	20	0	30
NIH052	AA	<60	No	SAA/VSAA	none	No	No	No	No	20	10	0	10	0	0	0	40
NIH067	AA	<60	No	NSAA	none	No	No	No	No	0	10	-10	10	0	0	0	10
NIH121	AA	60+	No	SAA/VSAA	none	No	No	No	No	20	10	10	10	0	0	0	50
NIH107	AA	<60	No	SAA/VSAA	none	No	No	No	Under1	20	10	0	10	0	0	-20	20
NIH212	AA	<60	No	SAA/VSAA	2.7	No	No	No	Under1	20	10	0	10	0	20	-20	40
NIH316	TBD	<60	No	SAA/VSAA	none	No	No	No	Under1	20	10	0	10	0	0	-20	20
NIH057	AA	60+	No	SAA/VSAA	none	No	No	No	No	20	10	10	10	0	0	0	50
NIH097	AA	<60	No	SAA/VSAA	1.3	No	No	No	No	20	10	0	10	0	20	0	60
NIH119	AA	<60	No	SAA/VSAA	95	No	No	No	No	20	10	0	10	0	20	0	60
NIH236	AA	<60	No	SAA/VSAA	none	No	No	No	No	20	10	0	10	0	0	0	40
NIH238	AA	60+	No	SAA/VSAA	none	No	No	No	No	20	10	10	10	0	0	0	50
NIH322	TBD	<60	No	SAA/VSAA	none	No	No	Yes	Under1	20	10	0	-20	0	0	-20	-10
NIH155	AA	<60	No	SAA/VSAA	none	No	No	No	No	20	10	0	10	0	0	0	40
NIH191	AA	<60	No	SAA/VSAA	none	No	No	No	No	20	10	0	10	0	0	0	40
NIH283	AA	60+	No	SAA/VSAA	none	No	No	No	No	20	10	10	10	0	0	0	50
196-1	TBD	<60	Yes	NSAA	na	No	No	Yes	Under1	0	-20	-10	-20	0	0	-20	-70
NIH134	AA	<60	No	SAA/VSAA	none	No	No	No	No	20	10	0	10	0	0	0	40
NIH098	AA	60+	No	SAA/VSAA	none	No	No	No	No	20	10	10	10	0	0	0	50
NIH099	AA	<60	No	SAA/VSAA	1.8	No	No	No	No	20	10	0	10	0	20	0	60

NIH126	AA	60+	No	SAA/VSAA	4.9	No	No	No	No	20	10	10	10	0	20	0	70
NIH193	AA	<60	No	SAA/VSAA	74.4	No	No	No	Under1	20	10	0	10	0	20	-20	40
NIH253	AA	60+	No	SAA/VSAA	none	No	No	No	No	20	10	10	10	0	0	0	50
NIH263	AA	<60	No	SAA/VSAA	2.7	No	No	No	No	20	10	0	10	0	20	0	60
292-1	TBD	<60	Yes	NSAA	none	Yes	No	No	Under1	0	-20	-10	-20	0	0	-20	-70
NIH182	AA	<60	No	SAA/VSAA	none	No	No	No	No	20	10	0	10	0	0	0	40
NIH281	AA	<60	No	NSAA	none	No	No	No	No	0	10	-10	10	0	0	0	10
NIH090	AA	<60	No	SAA/VSAA	none	No	No	No	No	20	10	0	10	0	0	0	40
NIH187	AA	<60	No	SAA/VSAA	none	No	No	No	No	20	10	0	10	0	0	0	40
NIH194	AA	<60	No	SAA/VSAA	none	No	No	No	No	20	10	0	10	0	0	0	40
NIH201	AA	<60	No	SAA/VSAA	none	No	No	No	No	20	10	0	10	0	0	0	40
NIH286	AA	<60	No	SAA/VSAA	none	No	No	No	No	20	10	0	10	0	0	0	40
NIH075	AA	<60	No	NSAA	none	No	No	No	Under1	0	10	-10	10	0	0	-20	-10
NIH131	AA	<60	No	SAA/VSAA	none	No	No	No	No	20	10	0	10	0	0	0	40
NIH209	AA	<60	No	SAA/VSAA	2	No	No	No	No	20	10	0	10	0	20	0	60
NIH271	AA	<60	No	SAA/VSAA	15.8	No	No	No	No	20	10	0	10	0	20	0	60
NIH274	AA	60+	No	SAA/VSAA	none	No	No	No	No	20	10	10	10	0	0	0	50
NIH285	AA	60+	No	SAA/VSAA	none	No	No	No	No	20	10	10	10	0	0	0	50
NIH035	AA	<60	No	NSAA	none	No	No	No	No	0	10	-10	10	0	0	0	10
NIH054	AA	<60	No	SAA/VSAA	none	No	No	No	No	20	10	0	10	0	0	0	40
NIH112	AA	<60	No	SAA/VSAA	none	No	No	No	No	20	10	0	10	0	0	0	40
NIH264	AA	60+	No	SAA/VSAA	none	No	No	No	No	20	10	10	10	0	0	0	50
NIH267	AA	<60	No	SAA/VSAA	98.2	No	No	No	No	20	10	0	10	0	20	0	60
NIH077	AA	<60	No	SAA/VSAA	none	No	No	No	No	20	10	0	10	0	0	0	40
NIH117	AA	<60	No	SAA/VSAA	1.3	No	No	No	No	20	10	0	10	0	20	0	60
NIH135	AA	<60	No	SAA/VSAA	12.4	No	No	No	No	20	10	0	10	0	20	0	60
NIH233	AA	<60	No	SAA/VSAA	none	No	No	No	No	20	10	0	10	0	0	0	40
NIH270	AA	60+	No	SAA/VSAA	none	No	No	No	No	20	10	10	10	0	0	0	50
NIH291	AA	<60	No	SAA/VSAA	3.6	No	No	No	No	20	10	0	10	0	20	0	60
NIH093	AA	<60	No	SAA/VSAA	none	No	No	Yes	No	20	10	0	-20	0	0	0	10
NIH133	AA	<60	No	SAA/VSAA	36.9	No	No	No	No	20	10	0	10	0	20	0	60
NIH224	AA	<60	No	SAA/VSAA	none	No	No	No	No	20	10	0	10	0	0	0	40
NIH255	AA	<60	No	SAA/VSAA	none	No	No	No	Under1	20	10	0	10	0	0	-20	20
NIH257	AA	<60	No	SAA/VSAA	none	No	No	No	No	20	10	0	10	0	0	0	40
NIH294	AA	60+	No	SAA/VSAA	2	No	No	No	No	20	10	10	10	0	20	0	70
NIH275	AA	<60	No	SAA/VSAA	none	No	No	No	No	20	10	0	10	0	0	0	40
74-1	TBD	<60	No	NSAA	na	Yes	No	No	Under1	0	10	-10	-20	0	0	-20	-40
NIH138	AA	60+	No	SAA/VSAA	none	No	No	No	No	20	10	10	10	0	0	0	50
NIH188	AA	<60	No	SAA/VSAA	none	No	No	No	No	20	10	0	10	0	0	0	40
NIH251	AA	<60	No	SAA/VSAA	none	No	No	No	No	20	10	0	10	0	0	0	40
NIH265	AA	60+	No	SAA/VSAA	none	No	No	No	No	20	10	10	10	0	0	0	50
NIH273	AA	60+	No	SAA/VSAA	none	No	No	No	No	20	10	10	10	0	0	0	50
NIH280	AA	60+	No	SAA/VSAA	none	No	No	No	No	20	10	10	10	0	0	0	50

NIH092	AA	<60	No	NSAA	none	No	No	No	No	0	10	-10	10	0	0	0	10
NIH048	AA	<60	No	SAA/VSAA	none	No	No	No	No	20	10	0	10	0	0	0	40
NIH064	AA	<60	No	SAA/VSAA	7.5	No	No	No	No	20	10	0	10	0	20	0	60
NIH111	AA	<60	No	SAA/VSAA	none	No	No	No	No	20	10	0	10	0	0	0	40
NIH192	AA	<60	No	SAA/VSAA	none	No	No	No	No	20	10	0	10	0	0	0	40
NIH260	AA	<60	No	SAA/VSAA	none	No	No	No	No	20	10	0	10	0	0	0	40
NIH120	AA	<60	No	SAA/VSAA	5.8	No	No	No	No	20	10	0	10	0	20	0	60
NIH178	AA	<60	No	SAA/VSAA	36.1	No	No	No	No	20	10	0	10	0	20	0	60
NIH103	AA	60+	No	SAA/VSAA	none	No	No	No	No	20	10	10	10	0	0	0	50
NIH204	AA	60+	No	SAA/VSAA	none	No	No	No	No	20	10	10	10	0	0	0	50
NIH261	AA	<60	No	SAA/VSAA	79.3	No	No	No	No	20	10	0	10	0	20	0	60
NIH287	AA	<60	No	SAA/VSAA	1.1	No	No	No	No	20	10	0	10	0	20	0	60
NIH125	AA	<60	No	SAA/VSAA	4	No	No	No	No	20	10	0	10	0	20	0	60
NIH181	AA	<60	No	SAA/VSAA	none	No	No	No	No	20	10	0	10	0	0	0	40
NIH278	AA	<60	No	SAA/VSAA	none	No	No	No	No	20	10	0	10	0	0	0	40
NIH069	AA	<60	No	SAA/VSAA	none	No	No	No	Under1	20	10	0	10	0	0	-20	20
NIH108	AA	<60	No	SAA/VSAA	none	No	No	No	No	20	10	0	10	0	0	0	40
NIH076	AA	<60	No	SAA/VSAA	1.3	No	No	No	No	20	10	0	10	0	20	0	60
NIH235	AA	<60	No	SAA/VSAA	none	No	No	No	No	20	10	0	10	0	0	0	40
NIH237	AA	<60	No	SAA/VSAA	none	No	No	Yes	No	20	10	0	-20	0	0	0	10
USP009	TBD	<60	No	NSAA	none	Yes	No	No	Under1	0	10	-10	-20	0	0	-20	-40
USP035	TBD	<60	No	NSAA	none	Yes	No	No	Under1	0	10	-10	-20	0	0	-20	-40
USP041	AA	<60	No	NSAA	none	No	No	No	No	0	10	-10	10	0	0	0	10
USP043	AA	<60	No	NSAA	na	No	No	No	No	0	10	-10	10	0	0	0	10
USP053	AA	<60	No	NSAA	none	No	No	No	No	0	10	-10	10	0	0	0	10
USP078	AA	<60	No	NSAA	none	No	No	No	No	0	10	-10	10	0	0	0	10
USP081	AA	<60	No	NSAA	na	No	No	No	No	0	10	-10	10	0	0	0	10
USP087	AA	<60	No	NSAA	none	No	No	No	No	0	10	-10	10	0	0	0	10
USP098	AA	<60	No	NSAA	58	No	No	No	No	0	10	-10	10	0	20	0	30
USP099	AA	60+	No	SAA/VSAA	none	No	No	No	No	20	10	10	10	0	0	0	50
USP102	AA	<60	No	NSAA	86	No	No	No	No	0	10	-10	10	0	20	0	30
USP103	AA	<60	No	NSAA	none	No	No	No	No	0	10	-10	10	0	0	0	10
USP107	AA	60+	No	NSAA	none	No	No	No	No	0	10	10	10	0	0	0	30
USP108	AA	60+	No	NSAA	none	No	No	No	No	0	10	10	10	0	0	0	30
USP124	AA	<60	No	NSAA	none	No	No	No	No	0	10	-10	10	0	0	0	10
USP146	AA	<60	No	NSAA	none	No	No	No	No	0	10	-10	10	0	0	0	10
USP151	AA	<60	No	NSAA	none	No	No	No	No	0	10	-10	10	0	0	0	10
USP156	AA	<60	No	NSAA	na	No	No	No	No	0	10	-10	10	0	0	0	10
USP037	AA	<60	No	SAA/VSAA	7.6	No	No	No	Under1	20	10	0	10	0	20	-20	40
USP040	AA	<60	No	SAA/VSAA	none	No	No	No	No	20	10	0	10	0	0	0	40
USP042	AA	<60	No	SAA/VSAA	none	No	No	No	Under1	20	10	0	10	0	0	-20	20
USP050	AA	<60	No	SAA/VSAA	none	No	No	No	No	20	10	0	10	0	0	0	40
USP052	AA	<60	No	SAA/VSAA	none	No	No	No	No	20	10	0	10	0	0	0	40

USP054	AA	<60	No	SAA/VSAA	none	No	No	No	Under1	20	10	0	10	0	0	-20	20
USP056	AA	<60	No	SAA/VSAA	none	No	No	No	Under1	20	10	0	10	0	0	-20	20
USP080	AA	<60	No	SAA/VSAA	26	No	No	No	No	20	10	0	10	0	20	0	60
USP100	AA	<60	No	SAA/VSAA	none	No	No	No	No	20	10	0	10	0	0	0	40
USP101	AA	<60	No	SAA/VSAA	9.7	No	No	No	No	20	10	0	10	0	20	0	60
USP110	AA	<60	No	SAA/VSAA	52	No	No	No	No	20	10	0	10	0	20	0	60
USP111	AA	<60	No	SAA/VSAA	none	No	No	No	No	20	10	0	10	0	0	0	40
USP113	AA	<60	No	SAA/VSAA	none	No	No	No	No	20	10	0	10	0	0	0	40
USP115	AA	<60	No	SAA/VSAA	14	No	No	No	No	20	10	0	10	0	20	0	60
USP116	AA	<60	No	SAA/VSAA	65	No	No	No	No	20	10	0	10	0	20	0	60
USP117	AA	<60	No	SAA/VSAA	none	No	No	No	No	20	10	0	10	0	0	0	40
USP118	AA	<60	No	SAA/VSAA	8	No	No	No	No	20	10	0	10	0	20	0	60
USP120	AA	<60	No	SAA/VSAA	none	No	No	No	No	20	10	0	10	0	0	0	40
USP121	AA	<60	No	SAA/VSAA	none	No	No	No	No	20	10	0	10	0	0	0	40
USP122	AA	60+	No	SAA/VSAA	none	No	No	No	No	20	10	10	10	0	0	0	50
USP125	AA	<60	No	SAA/VSAA	none	No	No	No	No	20	10	0	10	0	0	0	40
USP127	AA	<60	No	SAA/VSAA	none	No	No	No	No	20	10	0	10	0	0	0	40
USP131	AA	<60	No	SAA/VSAA	none	No	No	No	No	20	10	0	10	0	0	0	40
USP132	AA	60+	No	SAA/VSAA	none	No	No	No	No	20	10	10	10	0	0	0	50
USP134	AA	<60	No	SAA/VSAA	none	No	No	No	No	20	10	0	10	0	0	0	40
USP135	AA	60+	No	SAA/VSAA	none	No	No	No	No	20	10	10	10	0	0	0	50
USP136	AA	<60	No	SAA/VSAA	none	No	No	No	No	20	10	0	10	0	0	0	40
USP137	AA	<60	No	SAA/VSAA	none	No	No	No	No	20	10	0	10	0	0	0	40
USP139	AA	60+	No	SAA/VSAA	none	No	No	No	No	20	10	10	10	0	0	0	50
USP140	AA	60+	No	SAA/VSAA	none	No	No	No	No	20	10	10	10	0	0	0	50
USP143	AA	<60	No	NSAA	none	No	No	No	No	0	10	-10	10	0	0	0	10
USP144	AA	<60	No	SAA/VSAA	none	No	No	No	No	20	10	0	10	0	0	0	40
USP145	AA	<60	No	SAA/VSAA	none	No	No	No	No	20	10	0	10	0	0	0	40
USP147	AA	60+	No	SAA/VSAA	20	No	No	No	No	20	10	10	10	0	20	0	70
USP158	AA	<60	No	SAA/VSAA	3.5	No	No	No	No	20	10	0	10	0	20	0	60
USP017	TBD	<60	No	NSAA	none	Yes	No	No	Under1	0	10	-10	-20	0	0	-20	-40
USP002	TBD	<60	No	NSAA	none	Yes	No	No	Under1	0	10	-10	-20	0	0	-20	-40
USP026	TBD	<60	Yes	NSAA	none	No	No	No	No	0	-20	-10	10	0	0	0	-20
USP022	TBD	<60	No	NSAA	6	No	No	No	Under1	0	10	-10	10	0	20	-20	10
USP030	TBD	<60	No	NSAA	none	No	No	No	Under1	0	10	-10	10	0	0	-20	-10
USP048	TBD	<60	No	NSAA	none	No	No	No	Under1	0	10	-10	10	0	0	-20	-10
USP029	TBD	<60	No	NSAA	none	No	No	No	Under1	0	10	-10	10	0	0	-20	-10

Supplemental Dataset S4. UTSW cohort validation individual-level data and scoring

Age at diagnosis (years) refers to the patient's age at the time of confirmed bone marrow failure. Acute vs chronic classification reflects the interval between the first abnormal complete blood count (CBC) and diagnosis, with <1 year considered acute and >1 year chronic. Cytopenia severity is defined according to standard criteria. PNH clone size is reported as a continuous percentage, with ≥0.5% considered positive in this cohort. Somatic mutations (6pLOH, Del13q, and BCOR/BCORL1) are recorded individually; the presence of any of these alterations—PNH, 6pLOH, del(13)(q) as an isolated chromosomal change, or somatic mutations in BCOR/BCORL1—contributes to the somatic score. AA-associated conditions are defined in Supplemental Table S2, and IBMFS red flags are defined in Supplemental Table S1. Lymphocyte telomere length (TL) is considered abnormal if <1st percentile. IBMF genetic testing indicates whether germline testing was performed. Response to immunosuppressive therapy (IST) at 6 months is recorded as complete response (CR), partial response (PR), or no response (NR). Columns for severity, acuity, age, IBMFS red flag, AA-associated condition, AA somatic, and TL <1st percentile represent the number of points assigned for each factor. PASS represents the sum of points from each score component and serves as the final classification metric. The UTSW cohort had no data for 6pLOH, and that component was assigned 0 points.

StudyID	Diagnosis	Clnical and laboratory data at diagnosis												Individual Score Components							PASS SCORE
		Age at diagnosis (years)	Acute vs Chronic	Severity	PNH gran	6pLOH	Del13q	*BCOR/ BCORL1*	AA associated conditions	IBMF red flags	Lymphocyte TL	IBMF genetic testing	Response to IST at 6 months	1. Severity	2. Acuity	3. Age ≥60	4. IBMFS red flag	5. AA associated condition	6. AA somatic	7. TL <1st	
UTSW 1	AA	18	Acute	NSAA	3.8	NA	no	NA	Present (1)	No red flags	Over10	not done	CR	0	10	-10	10	10	20	0	40
UTSW 2	AA	64	Acute	SAA/VSAA	0.34	NA	no	no	Absent	No red flags	NA	not done	Refractory	20	10	10	10	0	0	0	50
UTSW 3	AA	58	Acute	SAA/VSAA	NA	NA	no	NA	Absent	Red flag present (7)	NA	done (negative)	PR	20	10	0	-20	0	0	0	10
UTSW 4	AA	34	Acute	SAA/VSAA	NA	NA	NA	NA	Absent	No red flags	NA	not done	PR	20	10	0	10	0	0	0	40
UTSW 5	AA	27	Acute	NSAA	14.1	NA	no	no	Absent	No red flags	NA	not done	CR	0	10	-10	10	0	20	0	30
UTSW 6	AA	27	Acute	SAA/VSAA	11.6	NA	no	no	Absent	No red flags	NA	not done	CR	20	10	0	10	0	20	0	60
UTSW 7	AA	26	Acute	NSAA	5.7	NA	no	no	Absent	No red flags	NA	not done	PR	0	10	-10	10	0	20	0	30
UTSW 8	AA	61	Acute	SAA/VSAA	NA	NA	NA	no	Absent	Red flag present (2)	NA	not done	Refractory	20	10	10	-20	0	0	0	20
UTSW 9	AA	57	Acute	NSAA	6.9	NA	no	no	Present (2)	No red flags	NA	not done	Refractory	0	10	-10	10	10	20	0	40
UTSW 10	AA	52	Acute	NSAA	0	NA	no	no	Absent	Red flag present (7)	NA	not done	NE	0	10	-10	-20	0	0	0	-20
UTSW 11	AA	67	Acute	SAA/VSAA	NA	NA	yes	no	Absent	Red flag present (2, 4)	1to10	done (negative)	NE	20	10	10	-20	0	20	0	40
UTSW 12	AA	35	Chronic	NSAA	NA	NA	NA	NA	Absent	Red flag present (2, 6)	NA	not done	PR	0	-20	-10	-20	0	0	0	-50
UTSW 13	AA	42	Acute	NSAA	0	NA	no	no	Absent	No red flags	NA	done (negative)	CR	0	10	-10	10	0	0	0	10
UTSW 14	AA	69	Acute	SAA/VSAA	0.18	NA	no	no	Absent	No red flags	NA	not done	PR	20	10	10	10	0	0	0	50
UTSW 15	AA	62	Acute	SAA/VSAA	NA	NA	no	no	Absent	No red flags	NA	not done	CR	20	10	10	10	0	0	0	50
UTSW 16	AA	50	Acute	SAA/VSAA	0	NA	no	NA	Absent	No red flags	NA	not done	NE	20	10	0	10	0	0	0	40
UTSW 17	AA	49	Acute	NSAA	0	NA	no	NA	Absent	No red flags	NA	not done	CR	0	10	-10	10	0	0	0	10
UTSW 18	AA	18	Acute	SAA/VSAA	0	NA	NA	NA	Absent	No red flags	NA	not done	CR	20	10	0	10	0	0	0	40
UTSW 19	AA	51	Acute	NSAA	4.47	NA	no	NA	Absent	No red flags	NA	not done	PR	0	10	-10	10	0	20	0	30
UTSW 20	AA	77	Acute	SAA/VSAA	0	NA	NA	NA	Absent	No red flags	NA	not done	PR	20	10	10	10	0	0	0	50
UTSW 21	AA	65	Acute	SAA/VSAA	0.55	NA	no	NA	Absent	No red flags	NA	not done	PR	20	10	10	10	0	20	0	70
UTSW 22	AA	29	Acute	SAA/VSAA	9.2	NA	no	NA	Absent	No red flags	NA	not done	PR	20	10	0	10	0	20	0	60
UTSW 23	AA	63	Acute	SAA/VSAA	0.5	NA	no	no	Absent	No red flags	NA	not done	Refractory	20	10	10	10	0	20	0	70
UTSW 24	AA	70	Acute	SAA/VSAA	0.19	NA	no	NA	Absent	No red flags	1to10	not done	PR	20	10	10	10	0	0	0	50
UTSW 25	AA	68	Chronic	NSAA	0	NA	no	NA	Absent	No red flags	NA	not done	NE (no IST)	0	-20	10	10	0	0	0	0
UTSW 26	AA	67	Acute	NSAA	0	NA	no	no	Present (6)	No red flags	NA	not done	CR	0	10	10	10	10	0	0	40
UTSW 27	AA	60	Chronic	NSAA	0	NA	no	no	Absent	No red flags	NA	not done	NE (no IST)	0	-20	10	10	0	0	0	0
UTSW 28	AA	67	Acute	SAA/VSAA	NA	NA	no	NA	Absent	No red flags	NA	not done	NR	20	10	10	10	0	0	0	50
UTSW 29	AA	53	Acute	NSAA	0.78	NA	no	NA	Absent	Red flag present (2)	NA	not done	PR	0	10	-10	-20	0	20	0	0

UTSW 30	AA	26	Acute	SAA/VSAA	0	NA	NA	NA	Absent	No red flags	NA	not done	Refractory	20	10	0	10	0	0	0	40
UTSW 31	AA	49	Acute	SAA/VSAA	NA	NA	NA	NA	Absent	No red flags	NA	not done	CR	20	10	0	10	0	0	0	40
UTSW 32	AA	72	Acute	SAA/VSAA	0	NA	no	NA	Absent	No red flags	NA	not done	Refractory	20	10	10	10	0	0	0	50
UTSW 33	AA	45	Acute	NSAA	6	NA	no	NA	Absent	No red flags	NA	not done	CR	0	10	-10	10	0	20	0	30
UTSW 34	AA	24	Acute	SAA/VSAA	NA	NA	no	NA	Absent	No red flags	NA	not done	PR	20	10	0	10	0	0	0	40
UTSW 35	AA	81	Acute	SAA/VSAA	0	NA	no	NA	Absent	No red flags	NA	not done	PR	20	10	10	10	0	0	0	50
UTSW 36	AA	69	Acute	SAA/VSAA	6.5	NA	NA	NA	Absent	Red flag present (2)	NA	not done	Refractory	20	10	10	-20	0	20	0	40
UTSW 37	AA	74	Acute	SAA/VSAA	NA	NA	NA	NA	Absent	No red flags	NA	not done	NE	20	10	10	10	0	0	0	50
UTSW 38	AA	33	Acute	SAA/VSAA	42	NA	no	NA	Absent	Red flag present (2)	NA	done (negative)	CR	20	10	0	-20	0	20	0	30
UTSW 39	AA	27	Acute	SAA/VSAA	NA	NA	no	NA	Absent	No red flags	NA	not done	CR	20	10	0	10	0	0	0	40
UTSW 40	AA	71	Chronic	NSAA	0.87	NA	no	NA	Absent	No red flags	NA	not done	CR	0	-20	10	10	0	20	0	20
UTSW 41	AA	30	Acute	NSAA	1.9	NA	NA	NA	Absent	No red flags	NA	not done	NE	0	10	-10	10	0	20	0	30
UTSW 42	AA	27	Acute	SAA/VSAA	4.99	NA	NA	no	Absent	No red flags	NA	not done	Refractory	20	10	0	10	0	20	0	60
UTSW 43	AA	17	Acute	NSAA	0	NA	no	NA	Absent	No red flags	NA	not done	CR	0	10	-10	10	0	0	0	10
UTSW 44	AA	23	Acute	NSAA	0.28	NA	yes	NA	Absent	No red flags	NA	not done	Refractory	0	10	-10	10	0	20	0	30
UTSW 45	AA	20	Acute	SAA/VSAA	78	NA	no	NA	Absent	No red flags	NA	not done	NE	20	10	0	10	0	20	0	60
UTSW 46	AA	22	Chronic	NSAA	1.8	NA	no	NA	Absent	No red flags	NA	done (negative)	CR	0	-20	-10	10	0	20	0	0
UTSW 47	AA	21	Acute	SAA/VSAA	6.69	NA	no	no	Absent	No red flags	NA	not done	NE	20	10	0	10	0	20	0	60
UTSW 48	AA	19	Acute	SAA/VSAA	0	NA	no	no	Absent	No red flags	NA	not done	NE	20	10	0	10	0	0	0	40
UTSW 49	AA	61	Acute	SAA/VSAA	NA	NA	NA	NA	Absent	No red flags	NA	not done	NE	20	10	10	10	0	0	0	50
UTSW 50	AA	63	Acute	SAA/VSAA	0	NA	no	no	Absent	No red flags	NA	done (negative)	Refractory	20	10	10	10	0	0	0	50
UTSW 51	AA	55	Acute	NSAA	0	NA	no	no	Absent	No red flags	NA	not done	NE (too sick to treat)	0	10	-10	10	0	0	0	10
UTSW 52	AA	54	Acute	SAA/VSAA	NA	NA	no	no	Absent	No red flags	NA	not done	NE (died 1 month after IST)	20	10	0	10	0	0	0	40
UTSW 53	AA	49	Acute	SAA/VSAA	3.5	NA	no	no	Absent	No red flags	NA	done (negative)	CR	20	10	0	10	0	20	0	60
UTSW 54	AA	52	Chronic	SAA/VSAA	96	NA	no	no	Absent	Red flag present (7)	NA	not done	Refractory	20	-10	0	-20	0	20	0	10
UTSW 55	AA	68	Acute	SAA/VSAA	0.24	NA	no	no	Absent	No red flags	NA	not done	NE (died 1 month after IST)	20	10	10	10	0	0	0	50
UTSW 56	AA	69	Acute	SAA/VSAA	NA	NA	no	no	Absent	No red flags	NA	not done	NE (started but didn't tolerate IST)	20	10	10	10	0	0	0	50
UTSW 57	AA	45	Acute	NSAA	0	NA	no	NA	Absent	Red flag present (7)	NA	not done	NE (no IST)	0	10	-10	-20	0	0	0	-20
UTSW 58	AA	45	Acute	NSAA	2	NA	no	NA	Absent	No red flags	NA	done (negative)	CR	0	10	-10	10	0	20	0	30
UTSW 59	AA	42	Acute	NSAA	0.14	NA	no	NA	Absent	No red flags	NA	not done	PR	0	10	-10	10	0	0	0	10
UTSW 60	AA	46	Acute	SAA/VSAA	0.12	NA	no	no	Absent	No red flags	NA	not done	NE	20	10	0	10	0	0	0	40
UTSW 61	AA	37	Acute	SAA/VSAA	1.15	NA	no	NA	Absent	No red flags	NA	done (negative)	PR	20	10	0	10	0	20	0	60

UTSW 62	AA	45	Acute	SAA/VSAA	0	NA	no	NA	Absent	No red flags	NA	done (negative)	PR	20	10	0	10	0	0	0	40
UTSW 63	AA	23	Acute	SAA/VSAA	NA	NA	NA	NA	lympho-proliferative disorder)	Red flag present (2)	NA	not done	CR	20	10	0	-20	10	0	0	20
UTSW 64	AA	31	Acute	NSAA	3.25	NA	no	NA	Absent	No red flags	Over10	done (negative)	CR	0	10	-10	10	0	20	0	30
UTSW 65	AA	71	Chronic	SAA/VSAA	4.3	NA	no	yes	Absent	No red flags	Over10	not done	PR	20	-10	10	10	0	20	0	50
UTSW 66	AA	62	Chronic	NSAA	0	NA	no	no	Absent	No red flags	NA	not done	PR	0	-20	10	10	0	0	0	0
UTSW 67	AA	23	Acute	NSAA	87	NA	no	yes	Absent	No red flags	NA	not done	NE	0	10	-10	10	0	20	0	30
UTSW 68	AA	73	Acute	SAA/VSAA	0	NA	no	no	Absent	No red flags	NA	not done	NE (no IST)	20	10	10	10	0	0	0	50
UTSW 69	AA	30	Acute	SAA/VSAA	0	NA	no	no	Absent	No red flags	NA	not done	NE	20	10	0	10	0	0	0	40
UTSW 70	AA	67	Acute	NSAA	0	NA	no	no	Absent	No red flags	NA	not done	NE	0	10	10	10	0	0	0	30
UTSW 71	AA	63	Acute	NSAA	0	NA	no	no	Absent	No red flags	NA	not done	NE (no IST)	0	10	10	10	0	0	0	30
UTSW 72	TBD	34	Acute	NSAA	0	NA	no	no	Absent	Red flag present (7)	Under 1	done (TERC)	NE (no IST)	0	10	-10	-20	0	0	-20	-40
UTSW 73	AA	28	Acute	SAA/VSAA	0	NA	no	no	Absent	No red flags	NA	done (negative)	NE	20	10	0	10	0	0	0	40
UTSW 74	TBD	62	Chronic	NSAA	0	NA	no	no	Present (3 - CVID)	No red flags	1to10	done (TERT)	NE (no IST)	0	-20	10	10	10	0	0	10
UTSW 75	TBD	31	Chronic	NSAA	0	NA	no	NA	Absent	Red flag present (2,7)	1to10	done (TERT x2)	NE	0	-20	-10	-20	0	0	0	-50
UTSW 76	TBD	65	Chronic	SAA/VSAA	0	NA	no	NA	Absent	Red flag present (2, 6, 7)	1to10	done (RTEL1)	NE (no IST)	20	-10	10	-20	0	0	0	0
UTSW 77	TBD	45	Chronic	NSAA	0	NA	no	no	Absent	Red flag present (2, 7)	Under 1	done (TERT)	NE (no IST)	0	-20	-10	-20	0	0	-20	-70
UTSW 78	TBD	37	Acute	NSAA	NA	NA	NA	NA	Absent	Red flag present (2)	Under 1	done (TERT)	CR	0	10	-10	-20	0	0	-20	-40

Supplemental Dataset S5. MD Anderson cohort validation individual-level data and scoring

Age at diagnosis (years) refers to the patient's age at the time of confirmed bone marrow failure. Acute vs chronic classification reflects the interval between the first abnormal complete blood count (CBC) and diagnosis, with <1 year considered acute and >1 year chronic. Cytopenia severity is defined according to standard criteria. PNH clone size is reported as a continuous percentage, with ≥0.5% considered positive. Somatic mutations (6pLOH, Del13q, and *BCOR/BCORL1*) are recorded individually; the presence of any of one or more PNH, 6pLOH, del(13)(q) as an isolated chromosomal change, or somatic mutations in *BCOR/BCORL1* count towards the somatic score. AA-associated conditions are defined in Supplemental Table S2, and IBMFS red flags are defined in Supplemental Table S1. Lymphocyte telomere length (TL) is considered abnormal if <1st percentile. IBMF genetic testing indicates whether germline testing was performed. Response to immunosuppressive therapy (IST) at 6 months is categorized as complete response (CR), partial response (PR), no response (NR), refractory, or not evaluable (NE). Columns for severity, acuity, age, IBMFS red flag, AA-associated condition, AA somatic, and TL <1st percentile represent the number of points assigned for each factor. PASS denotes the total score derived from summing individual points from each of the score components, used for final classification. One patient (MDA092) was excluded from the cohort due to a non-IBMF genetic condition (Turner's syndrome). The MDA cohort had no data for 6pLOH, and that component was assigned 0 points.

StudyID	Diagnosis	Clnical and laboratory data at diagnosis												Individual Score Components							PASS SCORE
		Age at diagnosis	Acute vs Chronic	Severity	PNH gran	6pLOH	Del13q	*BCOR/ BCORL1*	AA associated conditions	IBMF red flags	Lymphocyte TL	IBMF genetic testing	Response to IST at 6 months	1. Severity	2. Acuity	3. Age ≥60	4. IBMFS red flag	5. AA associated	6. AA somatic	7. TL <1st	
MDA001	AA	50	Acute	SAA/VSAA	NA	NA	NA	NA	Absent	No red flags	NA	not done	PR	20	10	0	10	0	0	0	40
MDA002	AA	25	Acute	SAA/VSAA	0	NA	NA	NA	Absent	No red flags	NA	not done	NE	20	10	0	10	0	0	0	40
MDA003	AA	24	Acute	SAA/VSAA	NA	NA	NA	NA	Absent	No red flags	NA	not done	Refractory	20	10	0	10	0	0	0	40
MDA004	AA	33	Acute	NSAA	NA	NA	NA	NA	Absent	No red flags	NA	not done	CR	0	10	-10	10	0	0	0	10
MDA005	AA	40	Acute	SAA/VSAA	12	NA	NA	NA	Absent	No red flags	NA	not done	NE	20	10	0	10	0	20	0	60
MDA006	AA	42	Acute	SAA/VSAA	0	NA	No	NA	Absent	No red flags	NA	not done	PR	20	10	0	10	0	0	0	40
MDA007	AA	35	Acute	SAA/VSAA	0	NA	No	NA	Absent	No red flags	NA	not done	PR	20	10	0	10	0	0	0	40
MDA008	AA	23	Acute	SAA/VSAA	0	NA	No	NA	Absent	No red flags	NA	done (negative)	PR	20	10	0	10	0	0	0	40
MDA009	AA	41	Acute	SAA/VSAA	NA	NA	No	NA	Absent	No red flags	NA	not done	PR	20	10	0	10	0	0	0	40
MDA010	AA	40	Acute	SAA/VSAA	0	NA	No	NA	Absent	No red flags	NA	not done	CR	20	10	0	10	0	0	0	40
MDA011	AA	24	Acute	SAA/VSAA	NA	NA	No	NA	Absent	No red flags	NA	not done	Refractory	20	10	0	10	0	0	0	40
MDA012	AA	48	Chronic	NSAA	0	NA	No	NA	Absent	No red flags	NA	not done	NE	0	-20	-10	10	0	0	0	-20
MDA013	AA	25	Acute	SAA/VSAA	0	NA	No	NA	Present (1)	No red flags	NA	not done	CR	20	10	0	10	10	0	0	50
MDA014	AA	22	Acute	NSAA	0	NA	Na	NA	Absent	No red flags	NA	not done	Refractory	0	10	-10	10	0	0	0	10
MDA015	AA	36	Acute	SAA/VSAA	0.06	NA	No	NA	Absent	No red flags	NA	not done	CR	20	10	0	10	0	0	0	40
MDA016	AA	53	Acute	SAA/VSAA	NA	NA	NA	NA	Absent	No red flags	NA	not done	Refractory	20	10	0	10	0	0	0	40
MDA017	AA	53	Acute	SAA/VSAA	0	NA	No	NA	Absent	No red flags	NA	not done	PR	20	10	0	10	0	0	0	40
MDA018	AA	21	Acute	SAA/VSAA	0	NA	No	NA	Absent	No red flags	NA	not done	CR	20	10	0	10	0	0	0	40
MDA019	AA	52	Chronic	NSAA	0.4	NA	No	NA	Absent	Red flag (7)	1to10	done (negative)	PR	0	-20	-10	-20	0	0	0	-50
MDA020	AA	58	Acute	SAA/VSAA	55	NA	No	NA	Absent	No red flags	NA	not done	Refractory	20	10	0	10	0	20	0	60
MDA021	AA	27	Acute	SAA/VSAA	0	NA	No	NA	Absent	No red flags	NA	not done	CR	20	10	0	10	0	0	0	40
MDA022	AA	27	Acute	SAA/VSAA	11.5	NA	No	NA	Absent	No red flags	NA	not done	PR	20	10	0	10	0	20	0	60
MDA023	AA	19	Acute	SAA/VSAA	27	NA	No	NA	Absent	No red flags	NA	not done	CR	20	10	0	10	0	20	0	60
MDA024	AA	57	Acute	SAA/VSAA	1.2	NA	No	NA	Absent	No red flags	NA	not done	CR	20	10	0	10	0	20	0	60
MDA025	AA	39	Acute	SAA/VSAA	1	NA	No	NA	Absent	No red flags	NA	not done	NE	20	10	0	10	0	20	0	60
MDA026	AA	22	Acute	SAA/VSAA	0	NA	No	NA	Absent	No red flags	NA	not done	CR	20	10	0	10	0	0	0	40
MDA027	AA	49	Acute	SAA/VSAA	0	NA	No	NA	Present (5, 7)	No red flags	NA	not done	PR	20	10	0	10	10	0	0	50
MDA028	AA	22	Chronic	SAA/VSAA	13	NA	No	NA	Absent	No red flags	NA	not done	PR	20	-10	0	10	0	20	0	40
MDA029	AA	50	Acute	SAA/VSAA	9.9	NA	No	NA	Absent	No red flags	NA	not done	PR	20	10	0	10	0	20	0	60
MDA030	AA	22	Acute	SAA/VSAA	7.5	NA	No	NA	Absent	No red flags	NA	not done	CR	20	10	0	10	0	20	0	60
MDA031	AA	30	Acute	SAA/VSAA	0.06	NA	No	NA	Absent	No red flags	NA	not done	CR	20	10	0	10	0	0	0	40

MDA032	AA	59	Acute	SAA/VSAA	0	NA	No	NA	Absent	No red flags	NA	not done	CR	20	10	0	10	0	0	0	40
MDA033	AA	21	Acute	SAA/VSAA	0	NA	No	NA	Absent	No red flags	NA	not done	PR	20	10	0	10	0	0	0	40
MDA034	AA	19	Acute	SAA/VSAA	1	NA	No	NA	Absent	No red flags	NA	not done	CR	20	10	0	10	0	20	0	60
MDA035	AA	29	Acute	NSAA	NA	NA	No	NA	Absent	No red flags	NA	not done	PR	0	10	-10	10	0	0	0	10
MDA036	AA	33	Acute	SAA/VSAA	3.2	NA	NA	NA	Absent	No red flags	NA	not done	CR	20	10	0	10	0	20	0	60
MDA037	AA	45	Acute	SAA/VSAA	0.9	NA	No	NA	Absent	No red flags	NA	not done	CR	20	10	0	10	0	20	0	60
MDA038	AA	44	Acute	NSAA	0.04	NA	No	NA	Absent	No red flags	NA	not done	CR	0	10	-10	10	0	0	0	10
MDA039	AA	54	Acute	SAA/VSAA	0	NA	No	NA	Absent	No red flags	NA	not done	CR	20	10	0	10	0	0	0	40
MDA040	AA	29	Acute	SAA/VSAA	0	NA	No	No	Absent	Red flag (4)	NA	done (negative)	CR	20	10	0	-20	0	0	0	10
MDA041	AA	46	Acute	NSAA	0.63	NA	No	NA	Absent	No red flags	NA	not done	CR	0	10	-10	10	0	20	0	30
MDA042	AA	20	Acute	SAA/VSAA	0	NA	No	NA	Absent	Red flag (7)	Over10	done (negative)	Refractory	20	10	0	-20	0	0	0	10
MDA043	AA	55	Acute	SAA/VSAA	0	NA	No	NA	Absent	No red flags	NA	not done	CR	20	10	0	10	0	0	0	40
MDA044	AA	24	Acute	NSAA	62.6	NA	No	Yes	Absent	No red flags	Over10	done (negative)	NE	0	10	-10	10	0	20	0	30
MDA045	AA	43	Acute	SAA/VSAA	0	NA	No	NA	Absent	No red flags	NA	not done	Refractory	20	10	0	10	0	0	0	40
MDA046	AA	19	Acute	SAA/VSAA	0	NA	No	No	Absent	No red flags	Over10	done (negative)	Refractory	20	10	0	10	0	0	0	40
MDA047	AA	49	Acute	SAA/VSAA	0	NA	No	NA	Present (5)	No red flags	NA	not done	Refractory	20	10	0	10	10	0	0	50
MDA048	AA	18	Chronic	SAA/VSAA	0.16	NA	No	No	Absent	No red flags	Over10	done (negative)	Refractory	20	-10	0	10	0	0	0	20
MDA049	AA	22	Acute	SAA/VSAA	0.22	NA	No	No	Absent	No red flags	Over10	done (negative)	CR	20	10	0	10	0	0	0	40
MDA050	AA	55	Acute	SAA/VSAA	0.23	NA	No	NA	Absent	No red flags	NA	not done	CR	20	10	0	10	0	0	0	40
MDA051	AA	28	Acute	SAA/VSAA	0	NA	No	NA	Absent	No red flags	NA	not done	PR	20	10	0	10	0	0	0	40
MDA052	AA	35	Acute	SAA/VSAA	11.8	NA	No	NA	Absent	No red flags	NA	not done	CR	20	10	0	10	0	20	0	60
MDA053	AA	19	Acute	SAA/VSAA	13.8	NA	No	No	Absent	No red flags	Over10	done (negative)	Refractory	20	10	0	10	0	20	0	60
MDA054	AA	24	Acute	SAA/VSAA	0.05	NA	No	No	Absent	No red flags	Over10	done (negative)	NE	20	10	0	10	0	0	0	40
MDA055	AA	25	Acute	SAA/VSAA	2.6	NA	No	No	Absent	No red flags	Over10	done (negative)	CR	20	10	0	10	0	20	0	60
MDA056	AA	23	Acute	SAA/VSAA	1.6	NA	No	NA	Absent	No red flags	Over10	done (negative)	PR	20	10	0	10	0	20	0	60
MDA057	AA	26	Acute	SAA/VSAA	6.8	NA	No	Yes	Absent	Red flag (7)	NA	done (negative)	CR	20	10	0	-20	0	20	0	30
MDA058	AA	25	Acute	SAA/VSAA	8.5	NA	No	NA	Absent	No red flags	NA	done (negative)	CR	20	10	0	10	0	20	0	60
MDA059	AA	19	Acute	NSAA	NA	NA	No	NA	Absent	No red flags	NA	not done	CR	0	10	-10	10	0	0	0	10
MDA060	AA	50	Acute	SAA/VSAA	0.51	NA	No	NA	Absent	No red flags	NA	not done	CR	20	10	0	10	0	20	0	60
MDA061	AA	30	Acute	SAA/VSAA	0	NA	No	NA	Absent	No red flags	NA	not done	PR	20	10	0	10	0	0	0	40
MDA062	AA	44	Chronic	NSAA	0	NA	No	NA	Absent	No red flags	1to10	done (negative)	PR	0	-20	-10	10	0	0	0	-20
MDA063	AA	56	Acute	SAA/VSAA	1.6	NA	No	Yes	Absent	No red flags	NA	not done	PR	20	10	0	10	0	20	0	60
MDA064	AA	26	Chronic	SAA/VSAA	0	NA	No	NA	Absent	No red flags	Over10	done (negative)	CR	20	-10	0	10	0	0	0	20

MDA065	AA	29	Acute	SAA/VSAA	0	NA	No	No	Absent	No red flags	NA	done (negative)	NE	20	10	0	10	0	0	0	40
MDA066	AA	32	Acute	SAA/VSAA	26	NA	No	No	Absent	No red flags	NA	done (negative)	CR	20	10	0	10	0	20	0	60
MDA067	AA	24	Acute	NSAA	1.38	NA	No	No	Present (1)	No red flags	Under 1	done (negative)	CR	0	10	-10	10	10	20	-20	20
MDA068	AA	32	Acute	SAA/VSAA	4.5	NA	No	No	Absent	No red flags	NA	done (negative)	CR	20	10	0	10	0	20	0	60
MDA069	AA	22	Acute	SAA/VSAA	14.6	NA	No	NA	Absent	No red flags	NA	not done	CR	20	10	0	10	0	20	0	60
MDA070	GATA2 deficiency	40	Acute	NSAA	0.44	NA	No	NA	Absent	No red flags	NA	done (GATA2)	NE	0	10	-10	10	0	0	0	10
MDA071	DBA	19	Chronic	NSAA	0	NA	No	NA	Absent	Red flag (1, 7)	NA	done (DBA)	NE	0	-20	-10	-20	0	0	0	-50
MDA072	GATA2 deficiency	26	Chronic	NSAA	0	NA	No	NA	Absent	No red flags	NA	done (GATA2)	NE	0	-20	-10	10	0	0	0	-20
MDA073	TBD	58	Acute	SAA/VSAA	0	NA	No	No	Absent	Red flag (2)	Under 1	done (TBD)	NE	20	10	0	-20	0	0	-20	-10
MDA074	TBD	5	Acute	SAA/VSAA	0	NA	No	No	Absent	No red flags	Under 1	done (TBD)	NE	20	10	0	10	0	0	-20	20
MDA075	TBD	65	Acute	NSAA	0	NA	No	No	Absent	Red flag (2)	Under 1	done (TBD)	NE	0	10	10	-20	0	0	-20	-20
MDA076	TBD	55	Acute	NSAA	0	NA	No	No	Absent	Red flag (2, 7)	Under 1	done (TBD)	NE	0	10	-10	-20	0	0	-20	-40
MDA077	TBD	78	Acute	NSAA	0	NA	No	No	Present (1)	Red flag (2)	Under 1	done (TBD)	NE	0	10	10	-20	10	0	-20	-10
MDA078	TBD	60	Chronic	NSAA	0	NA	No	No	Absent	Red flag (4)	Under 1	done (TBD)	NE	0	-20	10	-20	0	0	-20	-50
MDA079	SDS	10	Chronic	NSAA	0	NA	No	No	Absent	No red flags	NA	done (SBDS)	NE	0	-20	-10	10	0	0	0	-20
MDA080	SDS	43	Acute	NSAA	0	NA	No	No	Absent	Red flag (7)	NA	done (SBDS)	NE	0	10	-10	-20	0	0	0	-20
MDA081	Bloom	1	Chronic	NSAA	0	NA	No	No	Absent	Red flag (4)	NA	done (BLM)	NE	0	-20	-10	-20	0	0	0	-50
MDA082	DBA	1	Acute	SAA/VSAA	0	NA	No	NA	Absent	Red flag (7)	NA	done (DBA)	NE	20	10	0	-20	0	0	0	10
MDA083	DBA	13	Chronic	NSAA	NA	NA	No	NA	Absent	Red flag (1)	NA	done (RPL5)	NE	0	-20	-10	-20	0	0	0	-50
MDA084	DBA	44	Chronic	NSAA	NA	NA	No	NA	Absent	Red flag (1)	NA	done (RPS19)	NE	0	-20	-10	-20	0	0	0	-50
MDA085	FA	31	Chronic	NSAA	0	NA	No	NA	Absent	Red flag (4, 7)	NA	done (DEB)	NE	0	-20	-10	-20	0	0	0	-50
MDA086	FA	39	Chronic	NSAA	0	NA	No	NA	Absent	Red flag (1)	NA	done (DEB)	NE	0	-20	-10	-20	0	0	0	-50
MDA087	FA	7	Chronic	NSAA	NA	NA	No	NA	Absent	Red flag (1)	NA	done (DEB)	NE	0	-20	-10	-20	0	0	0	-50
MDA088	FA	28	Chronic	NSAA	0	NA	No	NA	Absent	Red flag (4)	NA	done (DEB)	NE	0	-20	-10	-20	0	0	0	-50
MDA089	FA	41	Chronic	NSAA	NA	NA	No	NA	Absent	Red flag (4)	NA	done (FANCA)	NE	0	-20	-10	-20	0	0	0	-50
MDA090	GATA2 deficiency	1	Acute	NSAA	NA	NA	NA	NA	Absent	Red flag (7)	NA	done (GATA2)	NE	0	10	-10	-20	0	0	0	-20
MDA091	GATA2 deficiency	55	Chronic	NSAA	NA	NA	No	NA	Absent	Red flag (2, 7)	NA	done (GATA2)	NE	0	-20	-10	-20	0	0	0	-50
MDA093	SDS	41	Chronic	NSAA	NA	NA	No	NA	Absent	Red flag (4)	NA	done (SBDS)	NE	0	-20	-10	-20	0	0	0	-50
MDA094	SDS	20	Chronic	NSAA	NA	NA	No	NA	Absent	No red flags	NA	done (SBDS)	NE	0	-20	-10	10	0	0	0	-20
MDA095	SDS	1	Chronic	NSAA	0	NA	No	NA	Absent	Red flag (1)	NA	done (SBDS)	NE	0	-20	-10	-20	0	0	0	-50
MDA096	TBD	30	Chronic	NSAA	NA	NA	No	NA	Absent	Red flag (3)	NA	done (TBD)	NE	0	-20	-10	-20	0	0	0	-50
MDA097	TBD	21	Acute	NSAA	0	NA	No	NA	Absent	No red flags	Under 1	done (TBD)	NE	0	10	-10	10	0	0	-20	-10
MDA098	TBD	22	Chronic	NSAA	NA	NA	No	NA	Present (3)	No red flags	Under 1	done (TBD)	NE	0	-20	-10	10	10	0	-20	-30

MDA099	TBD	41	Chronic	NSAA	0.1	NA	No	NA	Absent	No red flags	Under 1	done (TBD)	NE	0	-20	-10	10	0	0	-20	-40
MDA100	TBD	65	Chronic	NSAA	NA	NA	No	NA	Absent	Red flag (4)	Under 1	done (TBD)	NE	0	-20	10	-20	0	0	-20	-50
MDA101	TBD	76	Chronic	NSAA	NA	NA	No	NA	Absent	Red flag (7)	Under 1	done (TBD)	NE	0	-20	10	-20	0	0	-20	-50
MDA102	TBD	23	Chronic	NSAA	NA	NA	NA	Na	Absent	Red flag (7)	Under 1	done (TBD)	NE	0	-20	-10	-20	0	0	-20	-70
MDA103	TBD	30	Chronic	NSAA	NA	NA	No	NA	Absent	Red flag (7)	Under 1	done (TBD)	NE	0	-20	-10	-20	0	0	-20	-70
MDA104	TBD	53	Acute	NSAA	NA	NA	No	NA	Absent	Red flag (2)	Under 1	done (TBD)	NE	0	10	-10	-20	0	0	-20	-40
MDA105	TBD	49	Acute	NSAA	NA	NA	NA	NA	Absent	Red flag (6)	Under 1	done (TBD)	NE	0	10	-10	-20	0	0	-20	-40
MDA106	TBD	55	Chronic	NSAA	0	NA	No	NA	Absent	Red flag (7)	Under 1	done (TBD)	NE	0	-20	-10	-20	0	0	-20	-70
MDA107	TBD	46	Chronic	NSAA	NA	NA	No	NA	Absent	Red flag (7)	Under 1	done (TBD)	NE	0	-20	-10	-20	0	0	-20	-70
MDA108	TBD	40	Chronic	NSAA	NA	NA	No	NA	Absent	Red flag (1, 3)	Under 1	done (TBD)	NE	0	-20	-10	-20	0	0	-20	-70
MDA109	TBD	49	Chronic	NSAA	NA	NA	No	NA	Absent	Red flag (4)	Under 1	done (TBD)	NE	0	-20	-10	-20	0	0	-20	-70
MDA110	TBD	34	Chronic	NSAA	NA	NA	No	NA	Absent	Red flag (7)	Under 1	done (TBD)	NE	0	-20	-10	-20	0	0	-20	-70
MDA111	TBD	46	Acute	NSAA	NA	NA	No	NA	Absent	Red flag (4)	Under 1	done (TBD)	NE	0	10	-10	-20	0	0	-20	-40
MDA112	GATA2 deficiency	24	Acute	NSAA	0	NA	No	NA	Absent	Red flag (4, 7)	NA	done (GATA2)	NE	0	10	-10	-20	0	0	0	-20
MDA113	GATA2 deficiency	36	Acute	NSAA	NA	NA	No	NA	Absent	Red flag (7)	NA	done (GATA2)	NE	0	10	-10	-20	0	0	0	-20
MDA114	TBD	37	Acute	NSAA	0	NA	No	NA	Absent	Red flag (4)	Under 1	done (TBD)	NE	0	10	-10	-20	0	0	-20	-40
MDA115	LFS	37	Chronic	NSAA	NA	NA	No	Yes	Absent	Red flag (7)	NA	done (TP53)	NE	0	-20	-10	-20	0	20	0	-30
MDA116	TBD	71	Chronic	NSAA	NA	NA	No	No	Absent	Red flag (2)	Under 1	done (TBD)	NE	0	-20	10	-20	0	0	-20	-50
MDA117	TBD	41	Chronic	NSAA	0	NA	No	Yes	Absent	Red flag (2)	Under 1	done (TBD)	NE	0	-20	-10	-20	0	20	-20	-50
MDA118	TBD	35	Chronic	NSAA	0	NA	No	NA	Absent	Red flag (4)	Under 1	done (TBD)	NE	0	-20	-10	-20	0	0	-20	-70
MDA119	FA	0	Chronic	NSAA	0	NA	No	Yes	Absent	No red flags	NA	done (FANCA)	NE	0	-20	-10	10	0	20	0	0
MDA120	Germline ANKRD26	62	Acute	NSAA	NA	NA	No	NA	Absent	Red flag (4, 7)	NA	done (ANKRD26)	NE	0	10	10	-20	0	0	0	0
MDA121	Germline CBL	25	Chronic	NSAA	NA	NA	No	NA	Absent	No red flags	NA	done (CBL)	NE	0	-20	-10	10	0	0	0	-20
MDA122	GATA2 deficiency	36	Chronic	NSAA	NA	NA	No	No	Absent	Red flag (4)	NA	done (GATA2)	NE	0	-20	-10	-20	0	0	0	-50
